# A Major Role for the *Plasmodium falciparum* ApiAP2 Protein PfSIP2 in Chromosome End Biology

**DOI:** 10.1371/journal.ppat.1000784

**Published:** 2010-02-26

**Authors:** Christian Flueck, Richard Bartfai, Igor Niederwieser, Kathrin Witmer, Blaise T. F. Alako, Suzette Moes, Zbynek Bozdech, Paul Jenoe, Hendrik G. Stunnenberg, Till S. Voss

**Affiliations:** 1 Department of Medical Parasitology and Infection Biology, Swiss Tropical Institute, University of Basel, Basel, Switzerland; 2 Department of Molecular Biology, Nijmegen Center of Molecular Life Sciences, Radboud University, Nijmegen, The Netherlands; 3 Biozentrum, University of Basel, Basel, Switzerland; 4 School of Biological Sciences, Nanyang Technological University, Singapore; Albert Einstein College of Medicine, United States of America

## Abstract

The heterochromatic environment and physical clustering of chromosome ends at the nuclear periphery provide a functional and structural framework for antigenic variation and evolution of subtelomeric virulence gene families in the malaria parasite *Plasmodium falciparum*. While recent studies assigned important roles for reversible histone modifications, silent information regulator 2 and heterochromatin protein 1 (PfHP1) in epigenetic control of variegated expression, factors involved in the recruitment and organization of subtelomeric heterochromatin remain unknown. Here, we describe the purification and characterization of PfSIP2, a member of the ApiAP2 family of putative transcription factors, as the unknown nuclear factor interacting specifically with *cis*-acting SPE2 motif arrays in subtelomeric domains. Interestingly, SPE2 is not bound by the full-length protein but rather by a 60kDa N-terminal domain, PfSIP2-N, which is released during schizogony. Our experimental re-definition of the SPE2/PfSIP2-N interaction highlights the strict requirement of both adjacent AP2 domains and a conserved bipartite SPE2 consensus motif for high-affinity binding. Genome-wide *in silico* mapping identified 777 putative binding sites, 94% of which cluster in heterochromatic domains upstream of subtelomeric *var* genes and in telomere-associated repeat elements. Immunofluorescence and chromatin immunoprecipitation (ChIP) assays revealed co-localization of PfSIP2-N with PfHP1 at chromosome ends. Genome-wide ChIP demonstrated the exclusive binding of PfSIP2-N to subtelomeric SPE2 landmarks *in vivo* but not to single chromosome-internal sites. Consistent with this specialized distribution pattern, PfSIP2-N over-expression has no effect on global gene transcription. Hence, contrary to the previously proposed role for this factor in gene activation, our results provide strong evidence for the first time for the involvement of an ApiAP2 factor in heterochromatin formation and genome integrity. These findings are highly relevant for our understanding of chromosome end biology and variegated expression in *P. falciparum* and other eukaryotes, and for the future analysis of the role of ApiAP2-DNA interactions in parasite biology.

## Author Summary

*Plasmodium falciparum*, like many other unicellular pathogens, uses antigenic variation of surface proteins as a prime mechanism for immune evasion. Subtelomeric gene families, including the major virulence family *var*, encode these antigens, and recent studies highlighted the important contribution of the specific biology of chromosome ends in regulation of their expression. *P. falciparum* chromosome ends are enriched in epigenetic marks of eukaryotic heterochromatin; however, it remains unknown which proteins and regulatory DNA motifs are involved in the recruitment and organization of heterochromatin, and how they contribute to the role of subtelomere biology in this important pathogen. Here, we present the experimental identification and genome-wide characterization of PfSIP2, a member of the ApiAP2 family of transcription factors that are specific to *Plasmodium* and related apicomplexan parasites. PfSIP2 binds to a conserved recognition sequence exclusively in heterochromatic domains upstream of subtelomeric *var* genes and within telomere-associated repeat elements. Our results suggest important roles for this protein in several aspects of chromosome end biology, including *var* gene silencing. They are furthermore highly relevant for future efforts to dissect the specific biology of *P. falciparum* chromosome ends, and for our understanding of the role of ApiAP2 factors in parasite biology.

## Introduction

Throughout the eukaryotic kingdom, the overall structure of chromosome ends is conserved and characterized by the telomeric tract, composed of short G-rich repeats, and an extensive subtelomeric region consisting of various types and lengths of repeats, also known as telomere-associated sequences (TAS) [1]. This conservation underscores the functional importance of these domains in genome function and maintenance. Due to the heterochromatic nature of subtelomeric regions, genes located nearby are subject to epigenetic control and variegated expression [2]–[5]. Furthermore, subtelomeric domains promote frequent recombination events driving the evolution and diversity of gene families located close to chromosome ends [1],[3]. Pathogenic microorganisms exploit this system for antigenic variation of surface antigens to evade adaptive immune responses or to respond to other changes in environmental conditions [6].

The apicomplexan parasite *Plasmodium falciparum* causes the most severe form of malaria in humans with up to two million deaths annually [7]. Malaria symptoms are entirely associated with the erythrocytic phase of infection where repeated rounds of intra-erythrocytic parasite multiplication take place. Sequestration of infected red blood cell aggregates in the microvasculatory system, which is mediated by the binding of *P. falciparum* erythrocyte membrane protein 1 (PfEMP1) to a variety of endothelial receptors [8]–[11], represents one of the main contributors to severe disease, including cerebral and placental malaria [12]–[14]. PfEMP1 is encoded by the *var* gene family comprising approx. 60 mostly subtelomeric members [15]–[18]. Importantly, due to mutually exclusive transcription of *var* genes, only one PfEMP1 variant is exposed per parasite at any time and switches in *var* gene expression result in antigenic variation of PfEMP1 [17],[19] facilitating immune evasion and chronic infection. Recent studies highlighted the important contribution of the specific biology and dynamics of heterochromatic chromosome ends in the regulation of *var* genes and additional subtelomeric gene families coding for proteins involved in host-parasite interactions [20]–[27].

*P. falciparum* chromosome ends consist of a stretch of telomeric GGGTT(T/C)A repeats with an average size of 1.2 kb, followed by an extensive 20 to 40 kb TAS domain [28]. This region is composed of a conserved arrangement of so-called telomere-associated repeat elements (TAREs 1 to 6), each of which consists of distinct non-coding repeat arrays of varying length and sequence [29]. On all chromosome ends, the coding part of the genome directly downstream of TARE 6 is characterized by members of multiple antigen gene families including *var*, *rif*, *stevor* and *pfmc-2tm* [18]. Similar to other unicellular eukaryotes, *P. falciparum* chromosome ends associate into clusters that are anchored to the nuclear periphery [30]–[32]. This structurally conserved context facilitates meiotic recombination between *var* genes on heterologous chromosomes [30]. Interestingly, spontaneous chromosome breakage and telomere healing events create chromosome ends lacking the entire TARE region; while such chromosomes are still tethered to the nuclear periphery, they display a reduced association with other chromosome ends implicating a role for TARE in cluster formation [33].

Expression of *P. falciparum* subtelomeric gene families is clonally variant and restricted to only one member (or a few) in each family [19], [34]–[36]. Transgenes inserted into TARE 6 as well as endogenous *var* genes are reversibly silenced in a manner reminiscent to telomere-postion effect in other eukaryotes [25]. Recent genome-wide studies highlighted the striking and exclusive association of the repressive histone 3 lysine 9 tri-methylation mark (H3K9me3) and heterochromatin protein 1 (PfHP1) throughout the TAS region and adjacent gene families on all chromosomes [23],[27],[37]. These heterochromatic marks are also important in telomere-proximal gene silencing in *S. pombe* and higher eukaryotes [38],[39], indicating the existence of conserved epigenetic control strategies in highly divergent eukaryotes. The epigenetic changes underlying mutually exclusive *var* gene transcription and switching have been studied in some detail. Active *var* loci are enriched in acetylated H3K9 and H3K4me2/me3 [21], and the process of activation is linked to locus repositioning into an ill-defined transcriptionally active zone at the nuclear periphery [24],[31],[40]. Silenced *var* loci lack these activation marks and are enriched in H3K9me3 and PfHP1 instead [21],[22],[41]. Furthermore, silencing of *var* and a subset of *rif* genes is dependent on the two *P. falciparum* orthologs of silent information regulator 2 (PfSIR2) [20],[25],[26].

Overall, these results show that conserved epigenetic mechanisms that are also in place in other eukaryotes dictate heterochromatic silencing in *P. falciparum*. However, it remains completely unknown which proteins and *cis*-acting sequences are involved in the recruitment and organization of *P. falciparum* heterochromatin, and how they contribute to the important role of chromosome end biology in this pathogen. Our understanding of sequence-specific DNA-protein interactions in *P. falciparum* is negligible and mostly limited to the description of upstream sequence elements and their interaction with unknown nuclear proteins, and to *in silico* mapping of over-represented motifs in candidate promoter sequences [42]. This lack of knowledge is related to the extreme diversity of specific DNA-binding proteins in eukaryotes and the poor representation of such factors in the apicomplexan lineage [43]–[45]. Until recently, PfMYB1 was the only sequence-specific DNA-binding protein that had been investigated to some extent *in vivo* [46]. New impulses were given by the discovery of the lineage-specific expansion of the ApiAP2 family of putative transcription factors in apicomplexan parasites, characterized by the presence of plant-like AP2 DNA-binding domains [47]. The binding of three parasite AP2 domains to specific *cis*-acting elements upstream of *P. falciparum* genes *in vitro* has recently been demonstrated [48], and another study described an essential role for PbAP2-O in stage-specific transcription in *P. berghei* ookinetes [49].

Here, we identified a member of the ApiAP2 family as the unknown protein binding to SPE2 arrays upstream of subtelomeric *var* genes at the border of the non-coding and coding parts of *P. falciparum* chromosomes [50]. The SPE2-interacting protein, termed PfSIP2, contains two adjacent AP2 domains and is proteolytically processed *in vivo* to release a 60kDa functional N-terminal domain, PfSIP2-N. We show that both AP2 domains are strictly required for binding to the bipartite SPE2 motif. *In vivo*, PfSIP2-N is associated with over 700 SPE2 consensus sites that cluster upstream of subtelomeric *var* genes and in TARE2/3, but not to single chromosome internal sites. Consistent with this striking and exclusive binding of PfSIP2-N to heterochromatic regions, we found no effect of PfSIP2-N over-expression on global gene transcription. Instead, our results imply major roles for PfSIP2-N in *var* gene silencing and chromosome end biology.

## Results

### Purification and Identification of PfSIP2, a *P. falciparum* ApiAP2 Protein Interacting with SPE2 Motifs Upstream of Subtelomeric *var* Genes

**Figure 1:**
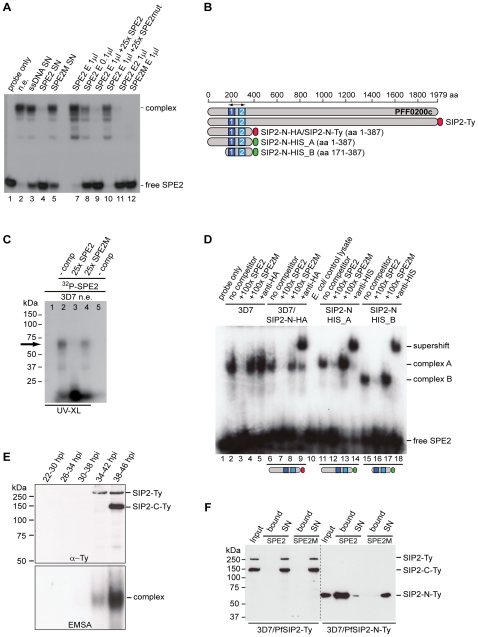
Identification of the ApiAP2 protein PfSIP2 as the SPE2-interacting protein. (A) Gel shift assay to test the course of affinity purification. Lane 1: probe only; lane 2: nuclear extract (input); lanes 3–5: supernatants after incubation of nuclear extract with beads carrying single-stranded DNA (3), SPE2 (4) or mutated SPE2M (5); lanes 7–10: proteins eluted from SPE2-loaded beads (SPE2 E). Lanes 11 and 12: A second eluate (SPE2 E2) and proteins bound to mutated SPE2M (SPE2mut E) contain no SPE2-binding activity. Faster migrating bands are probably due to degradation of PfSIP2-N during affinity purification. (B) Schematic representation of the ApiAP2 protein PfSIP2 encoded by PFF0200c and recombinant epitope-tagged PfSIP2 proteins expressed in either *P. falciparum* (SIP2-Ty; SIP2-N-HA; SIP2-N-Ty) or *E. coli* (SIP2-N-HIS_A; SIP2-N-HIS_B). Dark and light blue boxes indicated AP2 domains 1 and 2. (C) Size estimation of the endogenous SPE2-binding activity in parasite nuclear extracts by UV-crosslinking. The arrow highlights the size of the crosslinked DNA-protein complex (lanes 2 and 4). Complex formation is competed by a 25-fold molar excess of SPE2 (lane 3) but not by mutated SPE2M (lane 4). The DNA-protein complex is not observed in absence of radiolabeled SPE2 (lane 1) or without prior UV-crosslinking (lane 5). (D) Gel shift assay to confirm the identity of PFF0200c as the SPE2-binding protein. Lane 1: probe only; lanes 2–5: 3D7 nuclear extract; lanes 6–9: nuclear extract of 3D7/SIP2-N-HA over-expressing SIP2-N-HA (aa 1–387); lane 10: untransformed *E. coli* control lysate; lanes 11–14: lysate of *E. coli* expressing recombinant SIP2-N-HIS_A (aa 1–387); lanes 15–18: lysate of *E. coli* expressing recombinant SIP2-N-HIS_B (aa 171–387). Lanes 3, 7, 12 and 16: 100-fold molar excess of unlabeled SPE2 competitor. Lanes 4, 8, 13 and 17: 100-fold molar excess of mutated competitor SPE2M. Lanes 5, 9, 14 and 18: EMSA supershift in presence of anti-HA or anti-6×HIS antibodies. (E) Anti-Ty Western blot of 3D7/SIP2-Ty nuclear extracts prepared at five consecutive timepoints during the intra-erythrocytic developmental cycle (IDC). Expression of full-length PfSIP2-Ty and subsequent processing occur during schizogony. The bottom panel shows the presence of PfSIP2-N by EMSA in schizont extracts. Protein extracted from equal numbers of nuclei were used in each lane. hpi: hours post-invasion. (F) Pull-down of full-length PfSIP2-Ty and PfSIP2-N-Ty based on affinity to SPE2. PfSIP2-N-Ty binds efficiently to immobilized SPE2 whereas neither full-length PfSIP2-Ty nor the C-terminal processed fragment PfSIP2-C-Ty interact with SPE2. None of the proteins bound to mutated SPE2M DNA. SN: supernatant.

The bipartite SPE2 motif consists of two imperfect 6bp repeats separated by 4bp and its sequence-specific interaction with the unknown nuclear protein is only detectable after the onset of S-phase [50]. We used a high salt schizont stage nuclear extract, pre-cleared by incubation with single-stranded DNA, to purify the SPE2-binding activity based on its affinity to immobilized concatenated SPE2 elements. As control, mutated SPE2M motifs that are unable to interact with the protein were used [50]. EMSA monitoring showed that the SPE2-binding activity was efficiently depleted only after incubation with SPE2 but not SPE2M. Likewise, the activity was eluted only from beads carrying SPE2 but not SPE2M motifs (Figure 1A). Total protein eluted from both beads were precipitated separately, trypsinized and analyzed by LC-MS/MS. Peptide spectra were searched against a combined human and *P. falciparum* annotated protein database using TurboSequest software [51]. 89 and 82 *P. falciparum* proteins represented by two or more unique peptides were identified in the SPE2- and the SPE2M-bound fractions, respectively (Tables S1 and S2). Interestingly, of the 18 proteins exclusively detected in the SPE2 sample (Table 1), two proteins belong to the ApiAP2 family of transcription factors carrying putative sequence-specific AP2 DNA-binding domains, including the second-ranked protein encoded by PFF0200c. Furthermore, six co-purifying proteins have predicted roles in DNA and chromatin metabolism. In contrast, the 15 proteins detected exclusively in the control sample showed no such enrichment (Table S2). Due to the high peptide coverage and a temporal expression profile matching the presence of the SPE2-binding activity in stage-specific nuclear extracts [50],[52],[53], we considered PFF0200c the most likely candidate to encode the SPE2-binding activity.

**Table 1:** Putative SPE2-interacting protein candidates identified by LC-MS/MS.

PlasmoDB Accession^a^	Annotation	Protein probability	Protein score	MW kDa	Unique peptides
PF11_0099	heat shock protein DnaJ homologue Pfj2	1.15E-12	70.32	62.3	7
**PFF0200c**	**transcription factor with AP2 domains**	5.23E-07	70.24	229.5	7
PF08_0054	heat shock 70 kDa protein	5.65E-07	50.20	73.9	5
**PFB0840w**	**replication factor C, subunit 2**	4.29E-11	30.26	37.9	3
**PF13_0143**	**phosphoribosylpyrophosphate synthetase (PRS)**	1.01E-10	30.25	49.4	3
PF10_0323	early transcribed membrane protein 10.2	2.14E-06	30.19	38.9	3
**PFE0675c**	**deoxyribodipyrimidine photolyase, putative**	1.64E-04	30.13	129.1	3
PF13_0082	cop-coated vesicle membrane protein p24 precursor, putative	4.43E-08	20.27	24.4	2
**PFC0250c**	**AP endonuclease, putative**	2.06E-08	20.23	72.4	2
**PF10_0075**	**transcription factor with AP2 domains**	6.60E-08	20.22	182.6	2
MAL8P1.146	filament assembling protein, putative	5.61E-14	20.18	86.7	2
PFD0080c	PHIST domain protein	2.00E-05	20.17	60.2	2
MAL13P1.63	*P. falciparum* asparagine-rich protein	3.49E-06	20.16	139.0	2
PF11_0433	hypothetical protein	1.14E-06	20.16	324.6	2
**PFF1470c**	**DNA polymerase epsilon, subunit A**	3.62E-04	20.14	344.4	2
PF07_0040	lysophospholipase, putative	7.46E-04	20.13	42.3	2
PFB0915w	liver stage antigen 3	6.10E-04	20.12	175.6	2
**PF11_0053**	**PfSNF2L**	3.61E-11	16.24	167.4	2

a Accession numbers (www.plasmodb.org) of proteins exclusively detected in the SPE2-bound fraction (excluding seven annotated and putative ribosomal proteins). Proteins associated with DNA binding or metabolism are highlighted in bold.

PFF0200c encodes a large 230kDa protein containing two N-terminal AP2 domains as the only annotated features (Figure 1B). However, UV-crosslinking experiments revealed a 70kDa SPE2-protein complex, which is consistent with an estimated size of 50–60kDa of the SPE2-binding protein (Figure 1C). This discrepancy, and the fact that all seven PFF0200c-derived tryptic peptides mapped to the region containing both AP2 domains (Table S1), prompted us to consider possible proteolytic processing and release of a DNA-binding N-terminal fragment. We therefore expressed epitope-tagged N-terminal fragments of PFF0200c containing both AP2 domains as recombinant proteins in both *P. falciparum* and *E. coli* (Figure 1B). Nuclear extracts from 3D7/SIP2-N-HA parasites produced a SPE2-specific complex similar to the one observed in wild-type parasites (Figure 1D), and anti-HA antibodies specifically supershifted the complex obtained with the 3D7/SIP2-N-HA-derived extract only. Similarly, *E. coli* lysates containing the same fragment as 6×HIS tagged version (SIP2-N-HIS_A) produced a shift of similar size and specificity that was supershifted in presence of anti-6×HIS antibodies. Consistent with the smaller size of the SIP2-N-HIS_B protein, we observed a faster migrating complex of identical specificity. For completeness, we also expressed a 150kDa protein in *P. falciparum* containing all three AP2 domains of the second ApiAP2 protein PF10_0075 but were unable to detect binding to SPE2 (data not shown). Together, these results unambiguously identified PFF0200c as the *P. falciparum* SPE2-binding protein, which we termed PfSIP2 (SPE2-interacting protein).

### Proteolytic Processing of Inactive Full-Length PfSIP2 is Required to Release the SPE2-binding N-terminal Fragment PfSIP2-N

As indicated by gel shift and UV-crosslinking experiments, the sizes of the endogenous PfSIP2 activity and the N-terminal PfSIP2-N-HA protein were of similar size. To test if PfSIP2 is at all expressed as a full-length protein we generated a transgenic line expressing C-terminally tagged full-length PfSIP2 from the endogenous locus (3D7/SIP2-Ty) (Figure S1). Western analysis of nuclear extracts identified a 250kDa band, consistent with the predicted size of full-length PfSIP2-Ty, specifically in early and late schizonts (Figure 1E, top panel). An additional 150kDa C-terminal fragment (SIP2-C-Ty) appeared in late schizonts indicating that indeed a specific proteolytic event releases an N-terminal DNA-binding isoform. In line with this result, the same early and late schizont extracts produced a typical SPE2/PfSIP2-N complex in EMSA (Figure 1E, bottom panel). These results further suggested that full-length PfSIP2 is unable to interact with SPE2 *in vitro*. To test this, we performed SPE2 pull-down experiments and confirmed that only N-terminal PfSIP2-N-Ty bound to SPE2 beads, but not full-length PfSIP2-Ty nor the C-terminal fragment PfSIP2-C-Ty (Figure 1F). Together, these results substantiate the existence of a specific proteolytic event to activate the release of the functional DNA-binding protein PfSIP2-N during schizogony.

### PfSIP2-N Co-localizes with PfHP1 to Chromosome End Clusters and Interacts with SPE2 Arrays Upstream of *var* Genes *in vivo*

**Figure 2:**
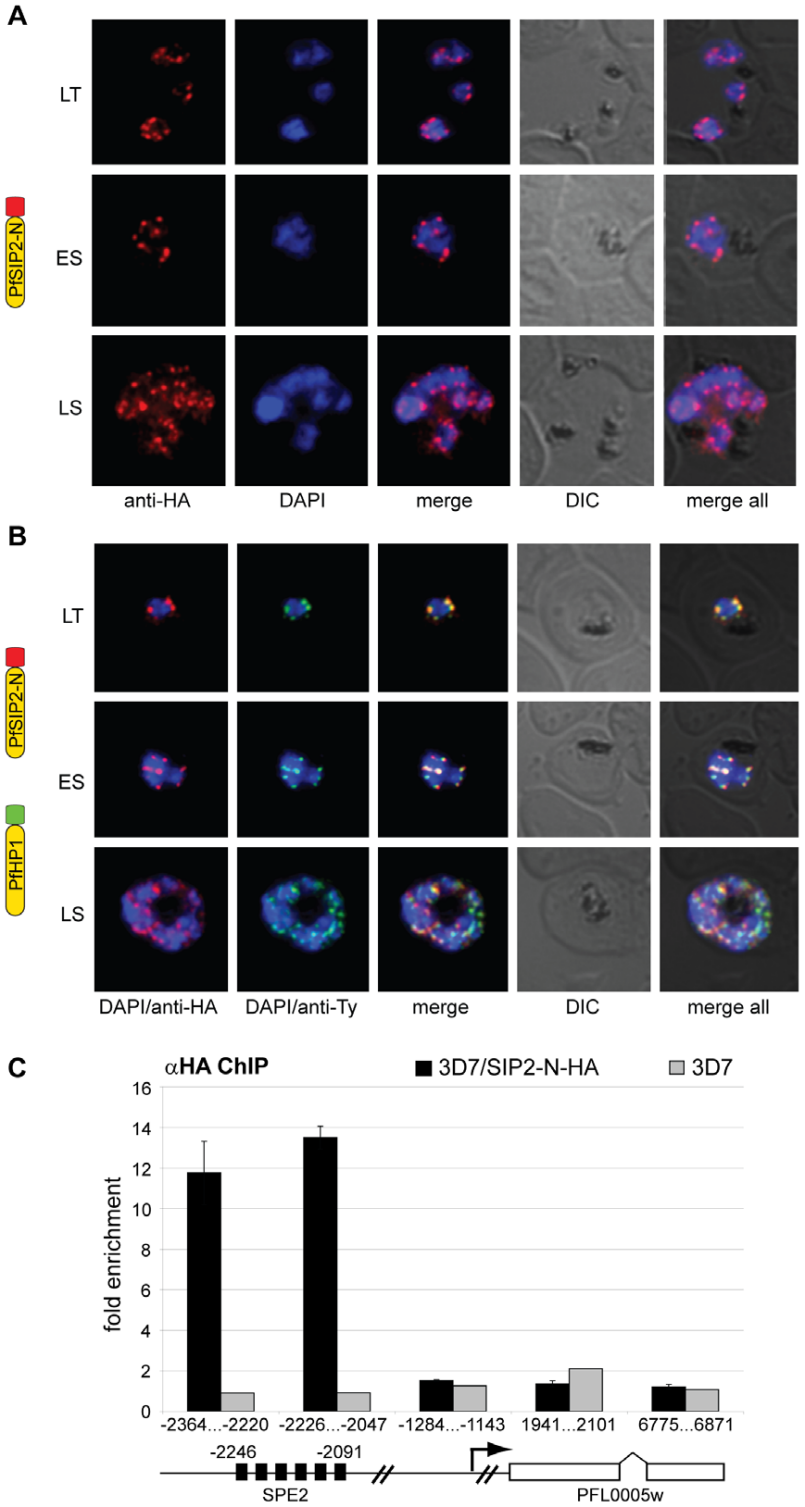
PfSIP2-N localizes to *P. falciparum* chromosome end clusters. (A) IFA detects discrete perinuclear PfSIP2-N-HA signals in late trophozoites (LT) and early (ES) and late (LS) schizont stage 3D7/SIP2-N-HA parasites. The expression cassette is schematically depicted on the left. (B) Co-localisation of PfSIP2-N with PfHP1-containing subtelomeric heterochromatin in late trophozoites (LT) and early (ES) and late (LS) schizonts in the double transgenic parasite line 3D7/SIP2-N-HA/HP1-Ty (over-expressing both proteins as epitope-tagged versions simultaneously). Expression cassettes are schematically depicted on the left. (C) Targeted ChIP-qPCR analysis demonstrates the specific binding of PfSIP2-N-HA to SPE2 upstream of upsB *var* gene PFL0005w in 3D7/SIP2-N-HA. Fold enrichment of cross-linked PfSIP2-N-HA-associated chromatin was determined for five regions across the PFL0005w locus. Values represent the mean of three independent experiments (error bars indicate the standard deviation). The location of the SPE2 repeat array and the positions of qPCR primers are indicated by nucleotide positions with respect to the ATG start codon. 3D7 wild-type parasites were used as negative control.

Since SPE2 arrays occur in conserved positions upstream of subtelomeric upsB *var* genes we expected PfSIP2-N to mark chromosome end clusters. Consistent with this assumption, indirect immunofluorescence (IFA) microscopy identified discrete PfSIP2-N-HA foci at the nuclear periphery with increasing numbers of foci in replicating stages (Figure 2A). To confirm that these signals indeed represented chromosome end clusters we compared the PfSIP2-N-HA signals with those of the heterochromatic marker PfHP1 [27] in a double transgenic line co-expressing PfSIP2-N-HA and PfHP1-Ty simultaneously. As expected, double-labeling IFAs revealed that both proteins co-localized at the nuclear periphery (Figure 2B). Next, we tested if PfSIP2-N binds to SPE2 elements *in vivo* by targeted chromatin immunoprecipitation (ChIP-qPCR). We observed specific enrichment of PfSIP2-N-HA at the SPE2 array upstream of the upsB *var* gene PFL0005w while three regions further downstream showed no association (Figure 2C). Together, these findings demonstrate that PfSIP2-N binds specifically to SPE2 arrays upstream of upsB *var* genes *in vivo*.

We recently demonstrated that *var* gene promoters driving expression of the drug-selectable marker h*dhfr* are silenced by default. Challenge with the antifolate WR99210 allowed selection for activated promoters, which displayed a ring stage-specific temporal activity profile similar to the endogenous promoters [24],[54]. To explore if PfSIP2 participates in the regulation of upsB *var* gene promoters, we used quantitative reverse transcriptase-PCR (qRT-PCR) to compare the activities of an episomal upsB promoter and a truncated version lacking a 500bp region containing the entire SPE2 array in transfected parasites lines 3D7/upsBR [54] and 3D7/upsBRΔSPE2, respectively, in a time course experiment (Figure S2). As expected, the default state of the wild-type upsB promoter in 3D7/upsBR was silenced. Interestingly, deletion of the region including the SPE2 array resulted in a ten-fold increase in default activity in all three ring stage samples, which is in line with previous results obtained by transient transfection [50]. In their activated states, however, both promoters displayed strong and almost identical activities and temporal profiles (Figure S2). These findings indicate that PfSIP2 may contribute to, but is not the only determinant of, upsB promoter-mediated silencing, and has no role in stage-specific *var* promoter activity.

### The Interaction of PfSIP2-N with SPE2 is Highly Sequence-Specific and Dependent on the Bipartite Nature of Both Interacting Partners

**Figure 3:**
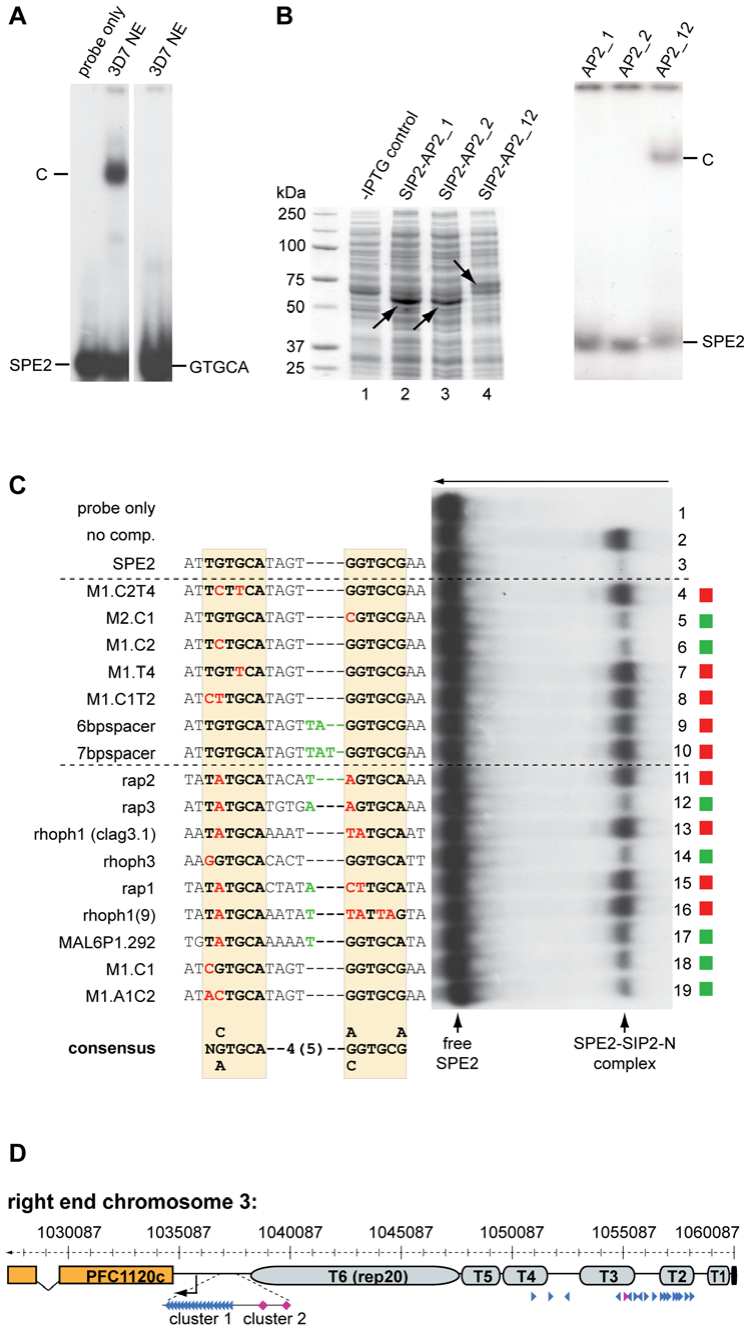
Binding of PfSIP2-N to SPE2 is highly sequence-specific and requires a bipartite motif and both adjacent AP2 domains. (A) SIP2-N in 3D7 nuclear extracts binds only to the bipartite SPE2 motif but not to a probe of identical length containing the SPE2 half site GTGCA only (GATACAT**GTGCA**AACATGAA). C: SPE2/PfSIP2-N complex. (B) Both AP2 domains are required for binding of PfSIP2-N to SPE2. Left panel: Coomassie-stained SDS-PAGE gel showing recombinantly expressed PfSIP2-GST fusions in *E. coli* lysates. Lane 1: non-induced control lysate; lane 2: SIP2-AP2_1 consisting of the first AP2 domain only (aa 174–252); lane 3: SIP2-AP2_2 carries the second AP2 domain only (aa 231–311); lane 4: SIP2-AP2_12 contains both adjacent AP2 domains (aa 174–311). Right panel: EMSA showing that single isolated AP2 domains are unable to bind to SPE2 and that both AP2 domains are required for binding. C: SPE2/PfSIP2-GST complex. (C) Competition EMSA using recombinant SIP2-N-HIS_B to determine the minimal sequence requirements for binding of PfSIP2-N to a SPE2 consensus site. The gel was rotated by 90° clockwise for simpler display (the arrow on top indicates the direction of electrophoretic separation). Lane 1: radiolabeled 28bp SPE2 probe only; lane 2: SPE2/PfSIP2-N-HIS_B interaction in absence of competitor; lanes 3–19: SPE2/PfSIP2-N-HIS_B interaction in presence of a 50-fold molar excess of specific competitors. The names and sequences of all competitors (all 28bp) are indicated to the left. The dashed lines group competitors into artificially mutated SPE2 motifs or into naturally occurring SPE2-like elements upstream of *P. falciparum* invasion genes [55] or upsB promoters (M1.C1, M1.A1C2). Altered nucleotides in the left or right half site of the original SPE2 sequence are highlighted in red. Additional nucleotides in the 4bp spacer are indicated in green. Red squares identify competitors unable to interact with PfSIP2-N-HIS_B, green squares highlight competitors that were able to compete with the SPE2/PfSIP2-N-HIS_B interaction. The experimentally determined SPE2 consensus site is shown at the bottom. (D) SPE2 consensus motifs define subtelomeric landmarks at *P. falciparum* chromosome ends. The position of all SPE2 motifs (arrowheads) at the right end of chromosome 3 is shown. Blue and purple arrowheads represent SPE2 motifs with four and five bp spacing between the half sites, respectively. Arrowhead orientation indicates the presence of SPE2 motifs on the sense or antisense strand. T1-T6: TARE 1 to 6. The telomere is depicted as a black box. The upsB *var* gene PFC1120c represents the first coding sequence downstream of TARE6. See also Tables S3 and S4 for more information. Chromosomal coordinates are according to PlasmoDB version5.5 annotation (www.plasmodb.org).

As an important step towards understanding the role of PfSIP2-N in parasite biology, we were interested in scrutinizing the specificity of the PfSIP2-N/SPE2 interaction. Our earlier work demonstrated that two point mutations in either half of the bipartite SPE2 sequence abrogated binding [50]. Intriguingly, a recent study identified GTGCA (which is identical to the first half of the bipartite SPE2 sequence) as consensus motif for PfSIP2-N [48]. These authors also argued that the first AP2 domain alone was sufficient for binding. To clarify these conflicting results, we compared the ability of PfSIP2-N to bind to a *bona fide* SPE2 motif and to a DNA sequence of identical length carrying the GTGCA motif only. Figure 3A shows that under identical conditions PfSIP2-N binds only to SPE2 but not GTGCA. We next speculated that the bipartite nature of both the SPE2 element and PfSIP2-N with two adjacent AP2 domains reflects the strict requirement of both intact modules for successful interaction. We expressed both AP2 domains separately or in combination as GST-fusions in *E. coli* and used gel shift assays to confirm that indeed both adjacent AP2 domains are required for binding (Figure 3B, right panel).

Next, we used gel shift competition assays to pinpoint as accurately as possible the minimal sequence requirements for a functional SPE2 consensus motif (Figure 3C and S3). A first set of 16 competitors incorporated single or multiple base deviations within the first and/or second half, and tested the importance of spacing between the half sites. The only changes tolerated were G to C at position one or two in the first or second repeat, respectively; all other changes completely averted binding. The spacing between the half sites was also critical with only four base pairs tolerated. A second set of competitors included eleven SPE2-like motifs naturally occurring upstream of genes coding for invasion-related proteins [55], and two untested SPE2 versions naturally present in SPE2 arrays upstream of *var* genes (M1.C1, M1.A1C2). Only three motifs competed efficiently (rap3, rhoph3, M1.A1C2) and two competed moderately (MAL6P1.292, M1.C1). These motifs are most closely related to the original SPE2 sequence. Furthermore, in two instances a 5bp spacer was tolerated. Interestingly, the rap2 (non-competing) and rap3 (competing) sequences are identical except for the fifth base in the spacer, indicating that these positions can also contribute to specificity. The competition EMSAs were repeated several times with independent batches of competitors and input protein (both nuclear extracts and *E. coli* lysates) and yielded identical results (Figure S3 and data not shown). This high degree of sequence-specificity of the PfSIP2-N/SPE2 interaction allowed us to deduce a functional SPE2 consensus motif (Figure 3C). Genome-wide *in silico* prediction using the consensus motif as query revealed a striking distribution of 777 putative PfSIP2-N binding sites throughout the genome (Tables S3 and S4). 330 sites (42.5%) are associated with the full set of 24 upsB *var* genes encoded in the genome. The majority of these (262) occur in sense-oriented tandem arrays approx. 2.2kb upstream of 23 subtelomeric upsB loci with an average of eleven motifs spaced by 12bp per locus, and the only chromosome-internal upsB *var* gene contains two upstream SPE2 sites. 66 sites define a second highly conserved cluster of upsB-associated SPE2 sites approx. 2.7 kb upstream of every subtelomeric locus. Interestingly, 393 sites (51.7%) are located in TAS concentrated in the TARE2/3 region on every chromosome end, with conserved position and orientation and an average of 18 motifs per chromosome end, most of which are spaced by 120bp. Figure 3D shows the right end of chromosome three as a representative example for the conserved arrangement of subtelomeric SPE2 sites on all chromosome ends. Of the remaining 45 sites (5.8%), 30 are located as single motifs in sense orientation upstream of mainly centrally located single copy genes coding for hypothetical proteins, and 15 sites map to coding regions or introns. In summary, this analysis identified a surprising pattern of putative PfSIP2-N-binding sites throughout the genome, with 94% of all predicted motifs confined to two major landmark regions in the subtelomeric domains of *P. falciparum* chromosomes.

### *In vivo* Binding of PfSIP2-N is Restricted to Subtelomeric SPE2 Landmarks in TAS and Upstream of UpsB-type *var* Genes

**Figure 4:**
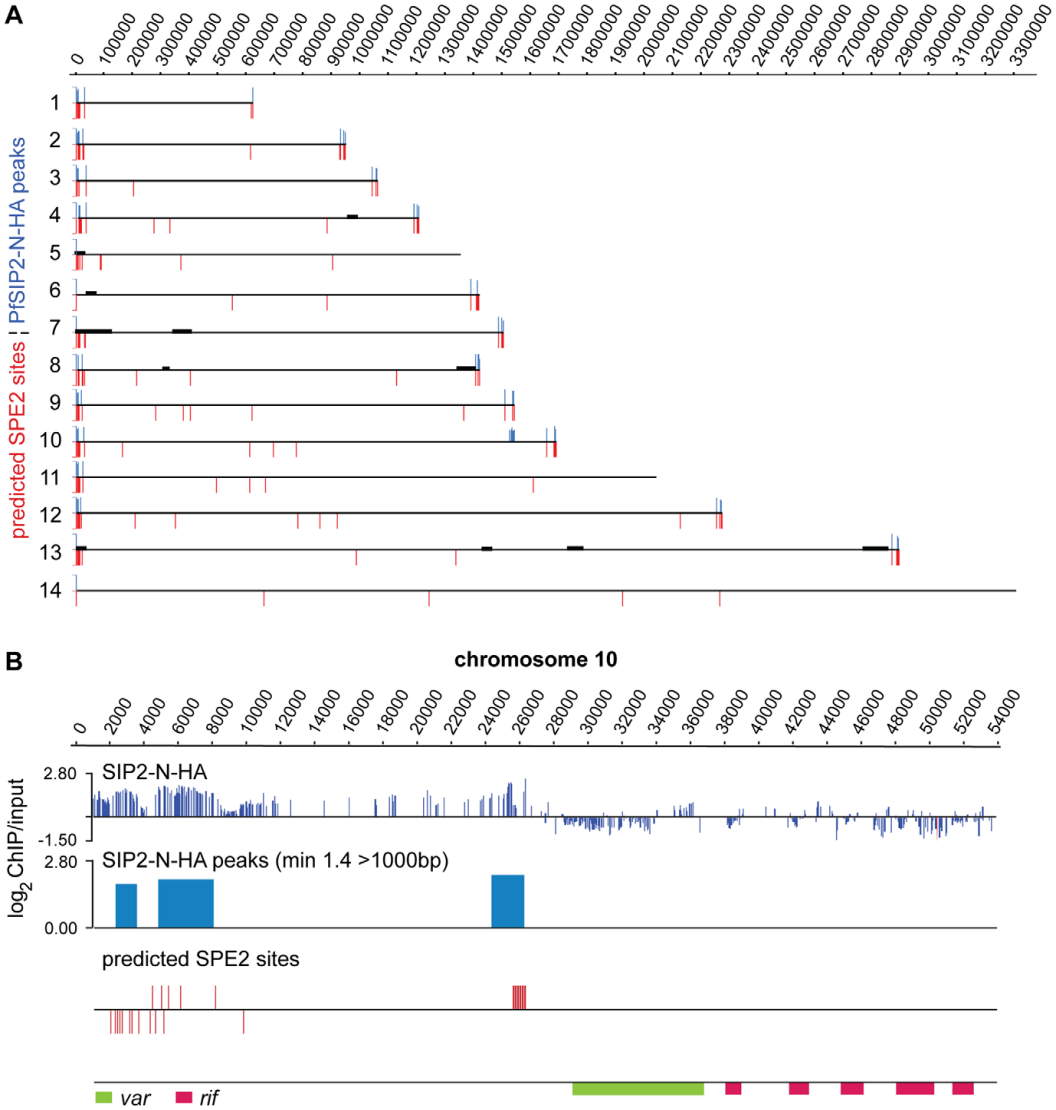
PfSIP2-N binds to SPE2 landmarks in subtelomeric regions of *P. falciparum* chromosomes *in vivo*. (A) Genome-wide PfSIP2-N-HA occupancy as determined by high-density ChIP-on-chip. Schematic display (SignalMap) and localization of genomic regions bound by PfSIP2-N-HA in 3D7/SIP2-N-HA schizont stage parasites (blue lines) and the position of predicted SPE2 consensus sites (red lines) on all 14 parasite chromosomes. Genomic regions were considered occupied by PfSIP2-N-HA if the average of log2 ratios (ChIP over input) for all probes in a 1000bp window was higher than 1.4. Chromosome numbers are indicated on the left, chromosomal positions on top. Solid black lines indicate regions not represented on the microarray (see also Table S3). (B) Regional zoom-in of the PfSIP2-N-HA ChIP-on-chip profile and predicted SPE2 motifs at the left arm of chromosome 10. PfSIP2-N-HA-occupied regions (peaks) have been identified by the built-in algorithm of SignalMap as explained above. The locations of the upsB *var* gene and downstream *rif* genes are indicated below. Chromosomal coordinates are according to PlasmoDB version5.5 annotation (www.plasmodb.org).

To test our *in silico* prediction and to identify potential additional PfSIP2-N target sites we performed genome-wide ChIP (ChIP-on-chip) on a high-density whole genome tiling array (NimbleGen Systems Inc.) [27],[37]. Comparison of the genome-wide PfSIP2-N-HA occupancy pattern with the *in silico* prediction revealed a high degree of overlap (Figure 4 and Table S3). Strikingly, the *in vivo* association of PfSIP2-N-HA was restricted to SPE2 sites in the predicted landmarks in TARE2/3 and upstream of subtelomeric *var* genes, both of which are located within H3K9me3/PfHP1-enriched heterochromatin [23],[27],[37]. In contrast, none of the chromosome-internal sites was bound by PfSIP2-N-HA and we observed no enrichment of PfSIP2-N-HA at non-SPE2 loci.

**Figure 5:**
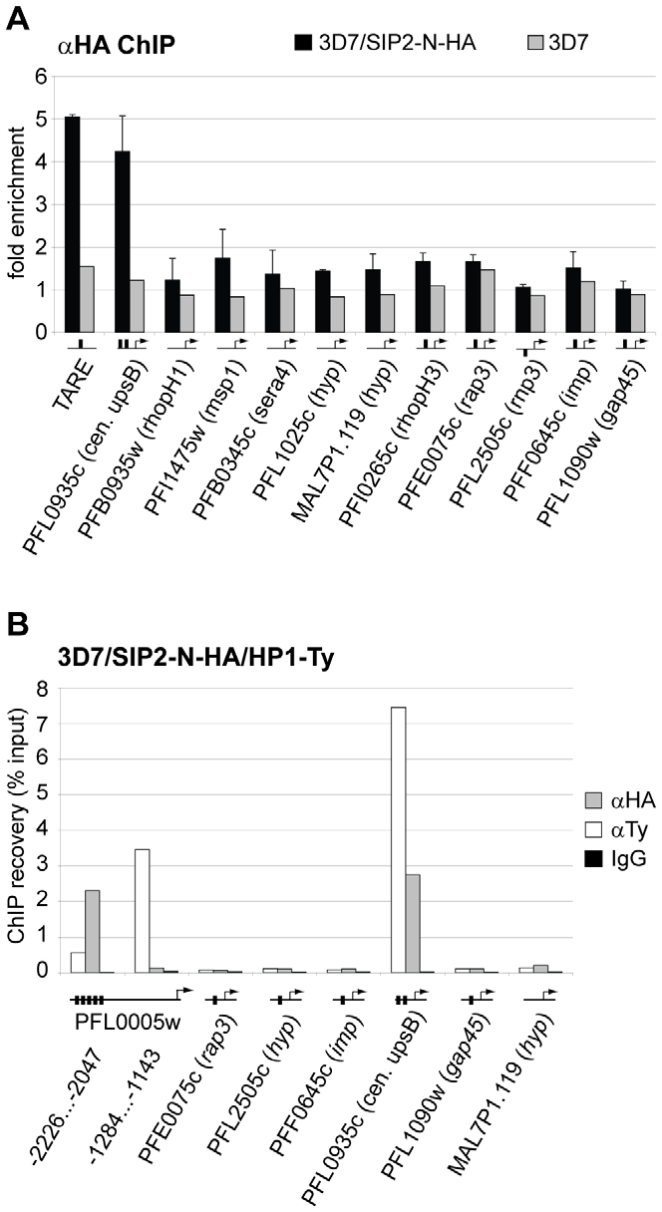
PfSIP2-N-HA binds exclusively to SPE2 elements located in heterochromatic TARE2/3 and upsB *var* gene domains. (A) PfSIP2-N-HA binds exclusively to SPE2 sites in TARE2/3 and upstream of upsB *var* genes. A region located between TARE2 and TARE3, the regions upstream of the only internal upsB *var* gene PFL0935c, promoters of five loci carrying predicted SPE2 consensus sites, and promoters of five genes lacking a SPE2 consensus site were tested by ChIP-qPCR for *in vivo* binding of PfSIP2-N-HA. qPCR primers were directed against upstream regions (represented by horizontal lines with arrows). Fold enrichment values represent the mean of three independent experiments on 3D7/SIP2-N-HA schizont stage samples (error bars indicate the standard deviation). 3D7 wild-type parasites were used as negative control. Gene names and accession numbers are according to PlasmoDB version5.5 (www.plasmodb.org). SPE2 elements are indicated by thick vertical black lines. (B) PfSIP2-N/SPE2 is embedded in PfHP1-containing heterochromatin. Parallel ChIP-qPCR was performed on 3D7/SIP2-N-HA/HP1-Ty schizont stage parasites using anti-HA and anti-Ty antibodies to test for occupancy by PfSIP2-N-HA and PfHP1-Ty, respectively. Normal rabbit IgG was used as negative control for ChIP. qPCR primers were directed against upstream regions (represented by horizontal lines with arrows). Vertical thick lines indicate the presence of SPE2 consensus sites. PfSIP2-N-HA and PfHP1-Ty co-occupancy at these loci was confirmed by ChIP-re-ChIP in independent experiments (Figure S4).

To validate the ChIP-on-chip data we performed ChIP-qPCR on selected loci in 3D7/SIP2-N-HA schizont stage parasites. Figure 5A shows that in addition to SPE2 upstream of upsB *var* genes (see Figure 2C), PfSIP2-N-HA was also bound to TARE2/3, and interestingly also to the only internal upsB *var* locus PFL0935c containing two juxtaposed SPE2 motifs. However, we found no specific enrichment at five promoters of internal genes carrying a single SPE2 site. As negative control, we tested the promoters of five genes that are not associated with SPE2. To confirm these results, and to test the anticipated co-occupancy of PfSIP2-N with PfHP1, which was previously shown to be enriched at upsB loci [27], we performed parallel ChIP using chromatin isolated from 3D7/SIP2-N-HA/HP1-Ty schizonts. Indeed, PfHP1 and PfSIP2-N were both enriched at a subtelomeric and the internal upsB locus (Figure 5B), corroborating the findings presented in Figures 2C and 5A. In independent experiments, we used ChIP-re-ChIP to directly confirm the co-occupancy of PfSIP2-N and PfHP1 on the same chromatin fragments in 3D7/SIP2-N-HA/HP1-Ty parasites (Figure S4). Interestingly, in both instances PfHP1 showed a marked reduction directly over the SPE2 array at the PFL0005w locus indicating that *in vivo* occupancy by PfSIP2-N might be incompatible with local nucleosome formation.

In summary, our combination of *in vitro*, *in silico* and *in vivo* characterisation uncovers a highly specific association of PfSIP2-N with SPE2 consensus motifs on a genome-wide level. The exclusive local restriction of this interaction to telomere-proximal non-coding regions implies important structural and functional roles for PfSIP2-N in subtelomeric heterochromatin formation and chromosome end biology.

### Over-expression of PfSIP2-N has no Effect on Transcriptional Regulation of Putative Target Genes

**Figure 6:**
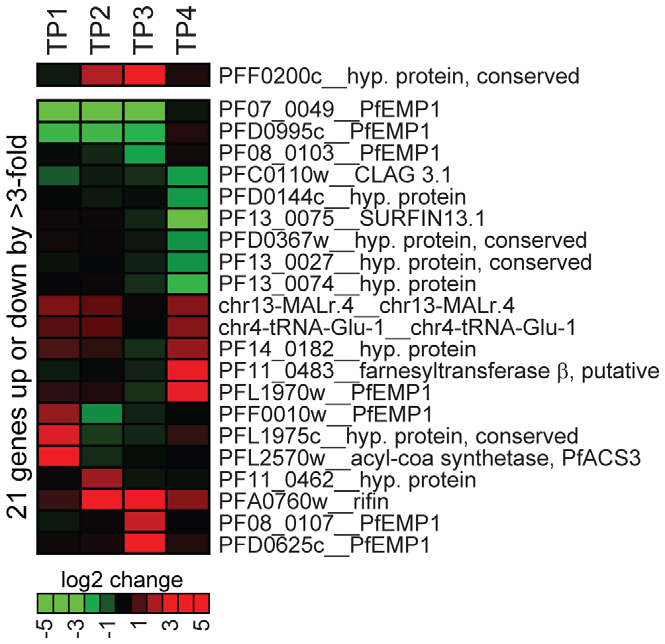
Overexpression of PfSIP2-N-HA has no effect on global gene transcription. Global transcript profiles in 3D7/SIP2-N-HA parasites were compared to the control line 3D7/camHG [27] at four timepoints across the IDC. TP1: ring stages 4–14 hours post-invasion (hpi); TP2: late ring stages 14–24 hpi; TP3: trophozoites 24–34 hpi; TP4: schizonts 32–42 hpi. All genes transcribed at greater 3-fold difference in 3D7/SIP2-N-HA compared to the control in at least one timepoint are listed. Over-expression of *pfsip2-n* is shown on top. The heat map indicates fold differences in transcript abundance on a gradual scale (green: down-regulated; red: up-regulated). Gene annotations are listed to the right and are according to PlasmoDB version5.5 (www.plasmodb.org).

To test a possible role for PfSIP2-N in transcriptional regulation by alternative means we compared the global transcript levels in 3D7/SIP2-N-HA parasites to a mock-transfected line at four stages during the intra-erythrocytic developmental cycle (IDC). Over-expression of PfSIP2-N-HA was evident by up to eight-fold higher levels of *pfsip2* transcripts in 3D7/SIP2-N-HA compared to the control (Figure 6). The overall effect of PfSIP2-N-HA over-expression was surprisingly minor with only 21 genes up- or down-regulated by more than three-fold in at least one time point. None of the de-regulated genes is associated with an upstream SPE2 motif and none of the putative PfSIP2-N target genes identified here or by others [48],[55] was affected, arguing strongly against a role of PfSIP2-N in transcriptional activation. Most of the affected genes, including seven non-upsB *var* genes, are located in heterochromatic regions, which can be explained by stochastic variation in the expression of heterochromatic genes between different isogenic cell lines.

## Discussion

Sequence-specific DNA-protein interactions serve to target specific activities to specific sites in the genome and are instrumental in genome organization and gene regulation. Here, we identified PfSIP2, a member of the ApiAP2 family of putative transcription factors, as the unknown nuclear protein binding to SPE2 tandem arrays upstream of subtelomeric *var* genes. Our comprehensive analysis reveals important novel insights into the nature and specificity of the PfSIP2/SPE2 interaction and provides compelling evidence for a major role of PfSIP2 in chromosome end biology.

Surprisingly, we found that the SPE2-binding activity is not exerted by the full-length protein but rather by a processed N-terminal fragment, PfSIP2-N. The inability of full-length PfSIP2 to interact with SPE2 *in vitro* indicates that the release of PfSIP2-N does not occur accidentally during extraction but reflects a true proteolytic cleavage event required to activate the DNA-binding activity. Our thorough re-definition of the specificity of the PfSIP2-N/SPE2 interaction in gel shift competition assays allowed us to determine a highly specific SPE2 consensus motif. Both 6bp half sites of the bipartite SPE2 element are strictly required for binding, which is in agreement with our earlier findings [50]. We also show that both adjacent AP2 domains are necessary for binding, probably through reinforcing interactions of each domain with each half of the bipartite motif. Such a scenario is consistent with the binding of single AP2 domains to single 5–6bp motifs in *Plasmodium* [48],[49] and plants [56],[57], and the specific interaction of the *Arabidopsis* tandem AP2-domain protein AINTEGUMENTA to a 16bp sequence, which also requires both AP2 domains for binding [58]. These findings challenge the previously proposed role of PfSIP2 in transcriptional activation of genes carrying an upstream GTGCA motif [48]. We clearly show that PfSIP2-N does not bind to GTGCA neither *in vitro* nor *in vivo* and, for that matter, consider PfSIP2-N unlikely to act as transcriptional activator of GTGCA-associated genes. We explain these conflicting results by fact that the protein binding microarray technology used in the former study was limited to the screening of random 10mers only, which precluded identification of the 16bp SPE2 element as high affinity binding motif. Our results are highly relevant for our understanding of the role of PfSIP2 in parasite biology, and provide important information for the future investigation of specific ApiAP2-DNA interactions.

Our genome-wide *in silico* prediction identified 777 PfSIP2-N target sites, 94% of which are located in two distinct landmark regions within subtelomeric heterochromatin on all chromosomes. One cluster corresponds to the previously described tandem arrays upstream of subtelomeric *var* genes [50], and a second newly identified cluster lies within TARE2/3. In addition, we detected 30 internal SPE2 sites located upstream of single copy genes. Importantly, genome-wide and targeted ChIP analysis of PfSIP2-N occupancy correlated strongly with the *in silico* prediction of subtelomeric target sites, showing that both subtelomeric landmark regions were bound by PfSIP2-N *in vivo*. In contrast, PfSIP2-N was absent at internal sites, except for the only internal upsB locus with two upstream SPE2 motifs. Therefore, binding of PfSIP2-N is restricted to heterochromatic regions including the entire subset of upsB *var* genes. The co-localization of PfSIP2-N with PfHP1 at perinuclear chromosome end clusters and upstream of upsB *var* genes corroborates this exclusive association. The observed increase in default upsB promoter activity upon deletion of the SPE2 array suggests a role for PfSIP2-N in *var* gene silencing, possibly through direct or indirect recruitment of effector proteins. However, the overall distribution pattern of PfSIP2-N implies important roles in structural and functional organization and maintenance of *P. falciparum* chromosome ends that go beyond regulating *var* gene expression.

In contrast to the well-established and conserved roles of telomere repeat-binding proteins ScRAP1, SpTAZ1, HsTRF1/2 in telomere position effect, telomere length regulation and heterochromatin formation in yeasts and humans, respectively [2], [59]–[64], specific DNA-protein interactions in TAS are hardly known and have only been investigated in detail in *S. cerevisiae*. One such factor is ABF1, which is involved in silencing, initiation of DNA replication, alteration of chromatin structure and nucleotide excision repair [65]–[70]. Our results are in agreement with similar roles of PfSIP2-N in *P. falciparum*. First, multiple attempts to generate a PfSIP2 knockout line failed due to refractoriness of the *pfsip2* locus to disruption (data not shown). Since *pfsip2* was readily accessible for 3′ replacement, we believe PfSIP2 is essential for parasite survival. Second, given the close connection between DNA replication and heterochromatin formation, the specific co-purification of several DNA replication/repair and chromatin remodeling factors (RFC, DNA pol ε, SNF2L, PRS) with PfSIP2-N (Table 1) supports a role in chromosome end maintenance. DNA pol ε and RFC, as well as members of the ATP-dependent chromatin remodeling complexes SWI/SNF, are central players in chromosome end replication and repair [71]–[75] and have important roles in silencing [76],[77]. Interestingly, these factors are also associated with DNA repair at stalled replication forks [78],[79], which can be induced by tight DNA-protein interactions and are crucially involved in recombination at rDNA repeats and mating type switching in *S. cerevisiae* and *S. pombe*, respectively [80]. Third, PfSIP2 expression and subsequent release of PfSIP2-N correlate with the DNA replication and nuclear division cycles during schizogony, and it is tempting to speculate that proteolytic activation of PfSIP2-N may occur in a cell cycle-dependent manner. A similar process has been described in activation of the CDP/Cut transcription factor by S phase-specific cleavage [81], and proteolytic processing of various targets, including cyclins, DNA replication factors and cohesin is an important regulatory strategy in cell cycle progression [82]. In summary, these results and observations are consistent with a potential multifunctional role of PfSIP2-N in chromosomal replication and/or segregation and in the nucleation of subtelomeric heterochromatin on newly replicated chromosomes.

ApiAP2 factors have been proposed to act as regulators of stage-specific expression [47],[48], and this was experimentally demonstrated for AP2-O in *P. berghei* ookinetes [49]. We mapped 45 SPE2 consensus sites in internal regions, mostly located upstream of single copy genes that are transcribed late during the IDC (Table S5). Another study identified SPE2-like motifs upstream of eleven genes coding for invasion-related proteins with similar expression profiles [55]. Interestingly, our ChIP experiments failed to reveal binding of PfSIP2-N to any of these sites. Although we cannot exclude that this lack of association is due to insufficient ChIP sensitivity or physical masking of the HA epitope at internal loci, we believe this finding reflects the true absence of this protein since PfSIP2-N was undoubtedly bound to the only chromosome-central upsB *var* locus carrying two SPE2 motifs. In addition, over-expression of PfSIP2-N had no effect on transcription of any of these genes. These observations are clearly inconsistent with a role for PfSIP2-N in transcriptional activation. However, we do not rule out a possible function of full-length PfSIP2 in regulation of target loci. First, given the overlap in expression of PfSIP2 with that of SPE2-asscociated genes, a role for PfSIP2 in their activation is conceivable. Although full-length PfSIP2 did not bind to SPE2 *in vitro*, it is still possible that PfSIP2 binds to and regulates target sites *in vivo*, possibly in association with other factors. Second, deletion of the SPE2 motif from the *rap3* promoter resulted in reduced activity [55] and, conversely, introduction of SPE2 into a heterologous promoter activated transcription in late-stage parasites [54]. Third, PfSIP2 orthologs exist in a subset of other apicomplexan parasites including all sequenced *Plasmodium* species, yet subtelomeric SPE2 arrays are unique to *P. falciparum*. In the *P. vivax* and *P. knowlesi* genomes, for instance, we predicted 120 and 80 SPE2 consensus sites, respectively, all of which occur as single sites mostly in chromosome-internal regions (data not shown). Hence, if SIP2 has the same binding specificity in other species (which is likely due to the remarkable sequence identity in their DNA-binding domains) then SIP2 must have a function other than being involved in subtelomere biology. It will be interesting to test if processing of PfSIP2 reflects a specific gain-of-function process during evolution of the *P. falciparum* lineage in order to cope with the massive expansion and control of subtelomeric virulence gene families.

In conclusion, we have identified the first sequence-specific component of subtelomeric regions in *P. falciparum*. To the best of our knowledge, PfSIP2-N/SPE2 represents a novel type of sequence-specific interaction at chromosome ends that has not been reported in any other eukaryote. Our results are highly relevant in the dissection of the specific biology of *P. falciparum* chromosome ends, which is key to the evolution and variable expression of subtelomeric virulence gene families, and will help to understand similar processes in other systems. Efforts to analyze a loss-of-function phenotype and to identify PfSIP2 interaction partners will be important future steps into this direction.

## Materials and Methods

### Parasite culture and transfection

*P. falciparum* 3D7 parasites were cultured as described previously [83]. Growth synchronisation was achieved by repeated sorbitol lysis [84]. Transfection constructs are described in Protocol S2. Transfections were performed as described [24] and selected on either 5µg/ml blasticidin-S-HCl or 4nM WR99210, or both. To obtain C-terminally tagged endogenous PfSIP2 parasites transfected with pSIP2-2×Ty_3′RP were subject to 3 cycles of growth in presence and absence of WR99210. Plasmid integration was verified by Southern analysis.

### Nuclear extracts and EMSA

High salt nuclear extracts and EMSAs were prepared and carried out as described [50] (see also Protocol S1). *E. coli* lysates were diluted to avoid excess input of recombinant protein. Competition EMSAs were performed in presence of non-specific competitor DNA (1µg sheared salmon sperm DNA and 200fmol random 30 base ss oligonucleotide per reaction) and a 50- to 100-fold molar excess of specific ds competitors.

### UV-crosslinking

Protein samples were incubated for 20min in EMSA buffer with 20fmol ^32^P-labeled SPE2 probe in presence or absence of a 25-fold molar excess of SPE2 or SPE2M competitors. DNA-protein interactions were UV-crosslinked for 60min (10^7^ Joule) in a Stratalinker 1800 (Stratagene) and separated by SDS-PAGE. Gels were directly exposed to X-ray film.

### Affinity purification and identification of the SPE2-binding protein

The complete protocol is explained in detail in Protocol S1. Briefly, the SPE2-binding activity was purified by incubation of schizont stage nuclear extracts with streptavidin magnetic beads carrying immobilized biotinylated SPE2 or SPE2M elements (see Protocol S4 for oligonucleotide sequences). Bound proteins were eluted with 2M KCl, precipitated with 10% TCA and dissolved in 50µl 100 mM Tris-HCl, pH 8.0. Proteins were digested with trypsin and analysed by capillary liquid chromatography tandem mass spectrometry (LC-MS/MS) using an Orbitrap FT hybrid instrument (Thermo Finnigan, San Jose, CA, USA). MS/MS spectra were searched against a combined *P. falciparum*/human annotated protein database.

### Southern blot analysis

To verify successful 3′ replacement at the PFF0200c locus, gDNA from 3D7 wild-type parasites and drug-cycled 3D7/SIP2-Ty parasites was digested with *BamH*I and *Hind*III and analysed by Southern blot. The blot was probed with a ^32^P-dATP-labeled 702bp fragment derived from the 3′ end of PFF0200c.

### Recombinant protein expression

Plasmids are described in Protocol S2. Recombinant proteins were expressed in *E. coli* Tuner (DE3) cells (Novagen) replicating pMICO [85]. After 4h induction using 1mM IPTG at 30°C, bacteria were pelleted and resuspended in 50mM Tris-HCl (pH7.5) containing protease inhibitors (Roche Diagnostics). The suspension was frozen, thawed and sonicated. NaCl, Triton X-100, β-ME and glycerol were added to final concentrations of 0.3M, 0.5%, 10mM and 5%, respectively, followed by centrifugation for 15min at 15,000g and 4°C. Supernatants were used in gel shift assays without further purification.

### Western blot analysis and protein pulldown

Primary antibody dilutions were: anti-HA 3F10 (Roche Diagnostics) 1∶2,000; anti-Ty BB2 (kind gift of K. Gull) 1∶10,000; anti-6×HIS (R&D Systems) 1∶5,000. High salt nuclear extracts from 3D7/SIP2-Ty and 3D7/SIP2-N-Ty schizonts were incubated for 1hr with streptavidin agarose beads carrying immobilized SPE2 or mutated SPE2M motifs in EMSA buffer supplemented with non-specific competitor DNA. After centrifugation at 2000rpm the supernatant was saved and beads washed three times in binding buffer. Bound proteins were eluted with 2M KCl.

### Immunofluorescence assays

Methanol-fixed cells were analysed using rat anti-HA 3F10 (1∶100) or mouse anti-Ty BB2 (1∶1,000) antibodies. Alexa-Fluor® 568-conjugated anti-rat IgG (Molecular Probes) 1∶500; FITC-conjugated anti-mouse IgG (Kirkegaard Perry Laboratories) 1∶300; TexasRed-conjugated anti-mouse IgG (Molecular Probes) 1∶500. Images were taken on a Leica DM 5000B microscope with a Leica DFC 300 FX camera and acquired via the Leica IM 1000 software and processed and overlayed using Adobe Photoshop CS2.

### Genome-wide SPE2 prediction

To identify the full complement of SPE2 consensus elements in *P. falciparum*, *P. vivax* and *P. knowlesi*, genome sequences available at PlasmoDB (version5.5) were searched using the PfSIP2-N-binding consensus sequence determined in competition gel shift assays (Figure 3C and Figure S3). A regular expression search engine (DREG) from the EMBOSS software package was used.

### ChIP and ChIP-on-CHIP

ChIP and ChIP-on-chip using formaldehyde-crosslinked chromatin was performed as described in detail elsewhere [27]. PfSIP2-N-HA enrichment at selected loci was tested by ChIP-qPCR using three independent chromatin preparations isolated from 3D7/SIP2-N-HA parasites, and by ChIP- and ChIP-re-ChIP-qPCR using two independent chromatin preparations isolated from the double-transfectant 3D7/SIP2-N-HA/HP1-Ty (qPCR primers are listed in Protocol S4). Enrichment values were calculated by dividing the recovery values obtained by ChIP with anti-HA 3F10 (Roche Diagnostics)/anti-Ty BB2 antibodies with that of the non-specific control IgG antibody. For ChIP-re-ChIP, chromatin fragments immuno-precipitated with anti-Ty BB antibodies were eluted with 50µl elution buffer (1% SDS and 0.1M NaHCO_3_). After incubation at 65°C for 10min to inactivate the BB2 antibody, the eluate was diluted 6× in incubation buffer lacking SDS (resulting in SDS concentration comparable to that of the first ChIP). Re-ChIP reactions were carried out in the presence of 0.2mg/ml Ty peptide to avoid immuno-precipitation by the BB2 antibody carried over from the first ChIP. Recovery values were defined in the percentage of the input material of the first ChIP. For genome-wide analysis, immunoprecipitated DNA was amplified by a modified T7 linear amplification method and analyzed on a tiling array (based on the May 2005 NCBI sequence of the *P. falciparum* genome; 385,000 probes with a median spacing of 48bp, Roche NimbleGen) [37].

### Quantitative reverse transcriptase PCR

qPCR was performed on reverse transcribed total RNA and gDNA isolated from synchronous parasite cultures at six timepoints across the IDC. A detailed protocol, relative transcript calculation and primer sequences are provided in Protocols S3 and S4.

### Transcriptional profiling and data analysis

Growth of 3D7/SIP2-N-HA parasites was tightly synchronized in parallel three times by sorbitol treatment to achieve a ten-hour growth window. Total RNA was isolated at four timepoints across the IDC at early ring stages (4–14 hours post-invasion (hpi)), late ring stages (14–24 hpi), trophozoites (24–34 hpi) and schizonts (32–42 hpi) by lysis of pelleted RBCs in TriReagent (Sigma). Transcript levels in 3D7/SIP2-N-HA were compared to those of the control line 3D7/camHG [27]. RNA samples were analyzed using a *P. falciparum* microarray as previously described [86]. RNA from each time point was hybridized against a RNA pool assembled from equal amounts of total RNA collected from the 3D7 strain at every eight hours across the IDC.

### Accession numbers

PlasmoDB (www.plasmoDB.org) accession numbers for genes and proteins discussed in this publication are: PfSIP2 (PFF0200c); PfHP1 (PFL1005c); upsB *var* genes (PFL0005w, PFL0935c); *rhoph1/clag2* (PFB0935w); *rhoph1/clag3.1* (PFC0120w); *rhoph1/clag9* (PFI1730w); *rhoph2* (PFI1445w); *rhoph3* (PFI0265c); *rap1* (PF14_0102); *rap2* (PFE0080c); *rap3* (PFE0075c); *rama* (MAL7P1.208); *rnp3* (PFL2505c); *imp*, putative (PFF0645c/MAL6P1.292); *gap45* (PFL1090w); *asp* (PFD0295c); *msp1* (PFI1475w); *sera4* (PFB0345c); conserved *Plasmodium* proteins (PFL1025c, MAL7P1.119); RFC, subunit 2 (PFB0840w); PfSNF2L (PF11_0053); DNA pol ε, subunit A (PFF1470c); transcription factor with AP2 domains (PF10_0075).

## Supporting Information

Figure S1 C-terminal tagging of endogenous PfSIP2. (A) Schematic display of the genomic context of PFF0200c encoding PfSIP2, the C-terminal tagging plasmid pSIP2-2xTy_3′RP, and the integration event. 702bp of the very 3′ end of PFF0200c fused in frame to a sequence coding for the 2xTy tag was used for homologous recombination into the endogenous locus. *BamH*I and *Hind*III restriction sites were used to digest gDNA from 3D7 and transfected parasites. Lengths of the resulting DNA fragments in bp are indicated. (B) Southern blot of *BamH*I/*Hind*III-digested genomic DNA from 3D7 and 3D7/SIP2-Ty parasites demonstrates the successful 3′ end replacement of the endogenous PFF0200c locus. Lane 1; pSIP2-2xTy_3′RP plasmid control; lane 2: 3D7 wild-type gDNA; lane 3: 3D7/SIP2-Ty gDNA after 2 cycles off and on WR99210 selection pressure; lane 4: 3D7/SIP2-Ty gDNA after three cycles off and on drug selection pressure. The membrane was probed with a 702bp encoding the 3′end of PFF0200c.
(0.01 MB PDF)

Figure S2 Evidence for an involvement of the PfSIP2/SPE2 interaction in *var* gene silencing. Reporter constructs testing the effect of a 500bp deletion including the entire SPE2 array on upsB promoter activity are depicted on top. Stage-specific promoter activity was determined by qRT-PCR in absence of WR selection (default; first graph) and after WR selection (activated; second graph). X-axis: synchronized cultures were harvested at six timepoints across the IDC. hpi: hours post-invasion. Y-axis: values represent relative transcript levels. Absolute *hdhfr*, *msp8* and *cam* transcript numbers were divided by those obtained for the constitutively expressed gene PF13_0170 (www.plasmoDB.org). *hdhfr* transcript levels were additionally normalized for differences in plasmid copy number as determined by qPCR (Protocol S3). Transcript profiles of *msp8* (ring stage-specific marker; third graph) and calmodulin (expressed in trophozoites and schizonts; last graph) show comparable stage composition for each parasite line in each timepoint.
(0.02 MB PDF)

Figure S3 Competition EMSA to determine a functional SPE2 consensus site. Competition EMSA using recombinant PfSIP2-N-HIS_A to determine the minimal sequence requirements for binding of PfSIP2-N to a SPE2 consensus site. The gel was rotated by 90° clockwise for simpler display. Lane 1: radiolabeled 28bp SPE2 probe only; lane 2: SPE2/PfSIP2-N-HIS_A interaction in absence of competitor; lanes 3–30: SPE2/PfSIP2-N-HIS_A interaction in presence of a 100-fold molar excess of specific competitors. The names of all competitors and the original SPE2 sequence and those of all competitors are indicated to the left. The dashed lines group competitors into artificially mutated SPE2 motifs and in naturally occurring SPE2-like elements upstream of *P. falciparum* invasion genes. Altered nucleotides in the left or right half site of the original SPE2 sequence are highlighted in red. Additional nucleotides in the 4bp spacer are indicated in green. The arrow on top indicates the direction of electrophoretic separation. Red squares identify competitors unable to interact with PfSIP2-N_HIS_A, green squares highlight competitors that were able to compete with the SPE2/PfSIP2-N-HIS_A interaction. The experimentally determined SPE2 consensus site is shown at the bottom.
(0.03 MB PDF)

Figure S4 ChIP-re-ChIP shows that PfSIP2-N-HA and PfHP1-Ty co-localise in subtelomeric heterochromatin. ChIP-re-ChIP was performed on 3D7/SIP2-N-HA/HP1-Ty schizont stage parasites. Top panel: Recovery of PfSIP2-N-HA- and PfHP1-Ty-associated chromatin after the first ChIP using anti-HA and anti-Ty antibodies, respectively, compared to the negative control (rabbit IgG). Second panel: Chromatin fragments immuno-precipitated with anti-Ty antibodies in the first ChIP were used as input and were re-ChIPped using anti-HA, anti-Ty and rabbit IgG antibodies in presence of competing Ty peptide. Efficient and specific peptide competition is evident from the failure to precipitate chromatin with anti-Ty antibodies in presence, but not in absence, of Ty peptide. Re-ChIP using anti-HA antibodies shows that PfSIP2-N-HA is present on PfHP1-Ty-enriched chromatin but only at loci associated with SPE2 (indicated by thick vertical lines). Bottom panel: Results from second panel displayed as fold enrichment values for PfSIP2-N-HA on PfHP1-Ty-enriched chromatin fragments. qPCR primers were directed against upstream regions (represented by horizontal lines with arrows). This experiment was repeated using independently isolated chromatin and yielded similar results.
(0.02 MB PDF)

Table S1 SEQUEST search results of peptide tandem mass spectra for proteins eluted with SPE2.
(0.08 MB PDF)

Table S2 SEQUEST search results of peptide tandem mass spectra for proteins eluted with SPE2M.
(0.08 MB PDF)

Table S3 Genome-wide prediction of SPE2 consensus motifs. The position of 777 SPE2 consensus motifs experimentally determined by competition EMSA are listed. Column 1: Chromosome ID. Column 2: nucleotide position with respect to the left telomere end of the first bp of the SPE2 motif. Column 3: nucleotide position of the last bp of the SPE2 motif with respect to the left telomere on each chromosome. Column 4: “+” denominates positive association of the corresponding SPE2 motif in the ChIP-on-chip experiment (threshold: min 1,000bp, >1.4 log2 ChIP over input). Column 5: SPE2 orientation on sense (“1”) or antisense (“−1”) strand. Column 6: “tel” indicates SPE2 location in TAREs, “var” indicates SPE2 location upstream of upsB var genes. SPE2 elements associated with genes that do not fall into the first to classes are identified by PlasmoDB annotation of the corresponding gene. Column 7: distance in bp to previous SPE2 motif. Column 8: blank cells represent canonical 4bp spacing between the two half sites of the bipartite SPE2 motif. “5” represents 5bp spacing. Column 9: PlasmoDB accession numbers of SPE2-associated genes. Column 10: SPE2 positions other than in TARE or upstream of coding sequences are indicated. Column 11: orientation of SPE2-associated genes on the sense (“1”) or antisense (“−1”) strand. Column 12: nucleotide position of the ATG start codon of SPE2-associated genes with respect to the left telomere on each chromosome. Values/information in individual columns was retrieved from PlasmoDB version 5.5 (www.plasmodb.org).
(0.03 MB PDF)

Table S4 Summary of the genome-wide location of SPE2 elements and their characteristics. Column 1: Classification of SPE2-associated loci into three groups (*var* genes, other genes, TARE). Column 2: The groups classified in column 1 are further subgrouped according to various criteria (see also Figure 3D). Column 3: total number of SPE2 sites in each subgroup. Column 4: number of SPE2-associated genes in each subgroup. Column 5: number of SPE2 sites in each subgroup that are located in either subtelomeric or chromosome-central positions. Column 6: number of SPE2 sites in each subgroup in either sense or antisense orientation with respect to the orientation of the SPE2-associated gene. Column 7: average distance between, and average number of, SPE2 sites in SPE2-defined subtelomeric landmarks. Column 8: number of SPE2 sites in each subgroup with either 4bp or 5bp spacing between half sites. Column 9: relative position of the most upstream (“first”) and downstream (“last”) SPE2 sites upstream of SPE2-associated genes.
(0.01 MB PDF)

Table S5 Single SPE2 sites upstream of internal genes. Column 1: Chromosome ID. Column 2: nucleotide position of the first bp of the SPE2 motif with respect to the left telomere end. Column 3: nucleotide position of the last bp of the SPE2 motif with respect to the left telomere on each chromosome. Column 4: SPE2 orientation on the sense (“1”) or antisense (“−1”) strand. Column 5: PlasmoDB gene annotation of associated genes. Column 6: PlasmoDB accession number of associated genes. Column 7: Location of SPE2 element with respect to the coding sequence. Column 8: blank cells represent canonical 4bp spacing between the two half sites of the bipartite SPE2 motif. “5” represents 5bp spacing. Column 9: orientation of SPE2-associated genes on the sense (“1”) or antisense (“−1”) strand. Column 10: nucleotide position of the ATG start codon of SPE2-associated genes with respect to the left telomere on each chromosome. Column 11: distance of SPE2 element in bp upstream of associated genes. Column 12: Peak transcription. Values/information in individual columns was retrieved from PlasmoDB version 5.5 (www.plasmodb.org).
(0.02 MB PDF)

Protocol S1 Affinity purification and mass spectrometry-based identification of the SPE2-interacting protein PfSIP2.
(0.03 MB DOC)

Protocol S2 Plasmid constructs.
(0.03 MB DOC)

Protocol S3 Quantitative reverse transcriptase PCR.
(0.03 MB DOC)

Protocol S4 Oligonucleotide sequences used in this study.
(0.04 MB DOC)
