# Supplementary figures and images for: A Major Role for the Plasmodium falciparum ApiAP2 Protein PfSIP2 in Chromosome End Biology
Source: PLoS Pathog. 2010 Feb 26;6(2):e1000784. doi: 10.1371/journal.ppat.1000784 (PMC2829057; doi:10.1371/journal.ppat.1000784)

Figure S1

A

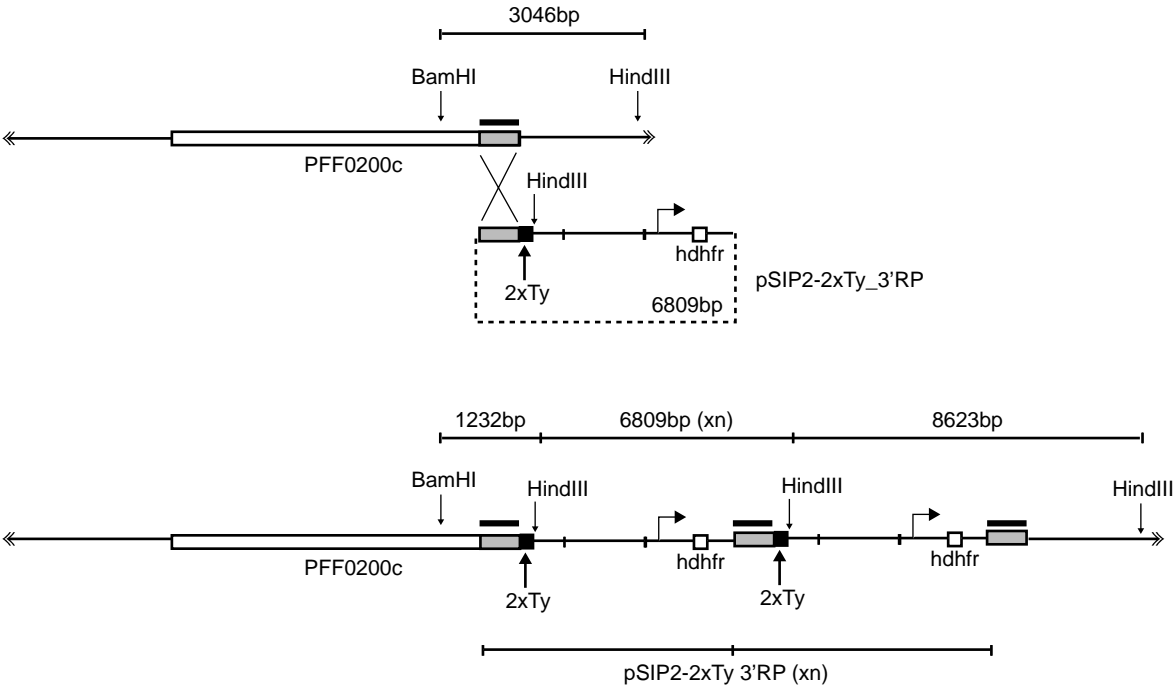

B

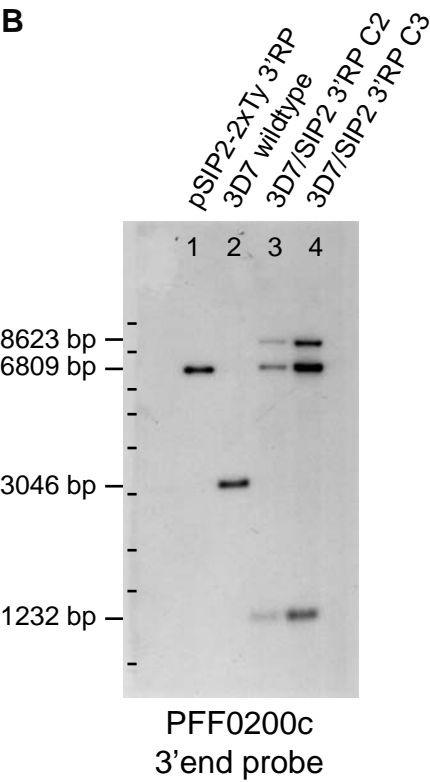

Supplement: Figure S1 — C-terminal tagging of endogenous PfSIP2. (A) Schematic display of the genomic context of PFF0200c encoding PfSIP2, the C-terminal tagging plasmid pSIP2-2xTy_3′RP, and the integration event. 702bp of the very 3′ end of PFF0200c fused in frame to a sequence coding for the 2xTy tag was used for homologous recombination into the endogenous locus. BamHI and HindIII restriction sites were used to digest gDNA from 3D7 and transfected parasites. Lengths of the resulting DNA fragments in bp are indicated. (B) Southern blot of BamHI/HindIII-digested genomic DNA from 3D7 and 3D7/SIP2-Ty parasites demonstrates the successful 3′ end replacement of the endogenous PFF0200c locus. Lane 1; pSIP2-2xTy_3′RP plasmid control; lane 2: 3D7 wild-type gDNA; lane 3: 3D7/SIP2-Ty gDNA after 2 cycles off and on WR99210 selection pressure; lane 4: 3D7/SIP2-Ty gDNA after three cycles off and on drug selection pressure. The membrane was probed with a 702bp encoding the 3′end of PFF0200c. (0.01 MB PDF) [file ppat.1000784.s001.pdf]

Figure S2

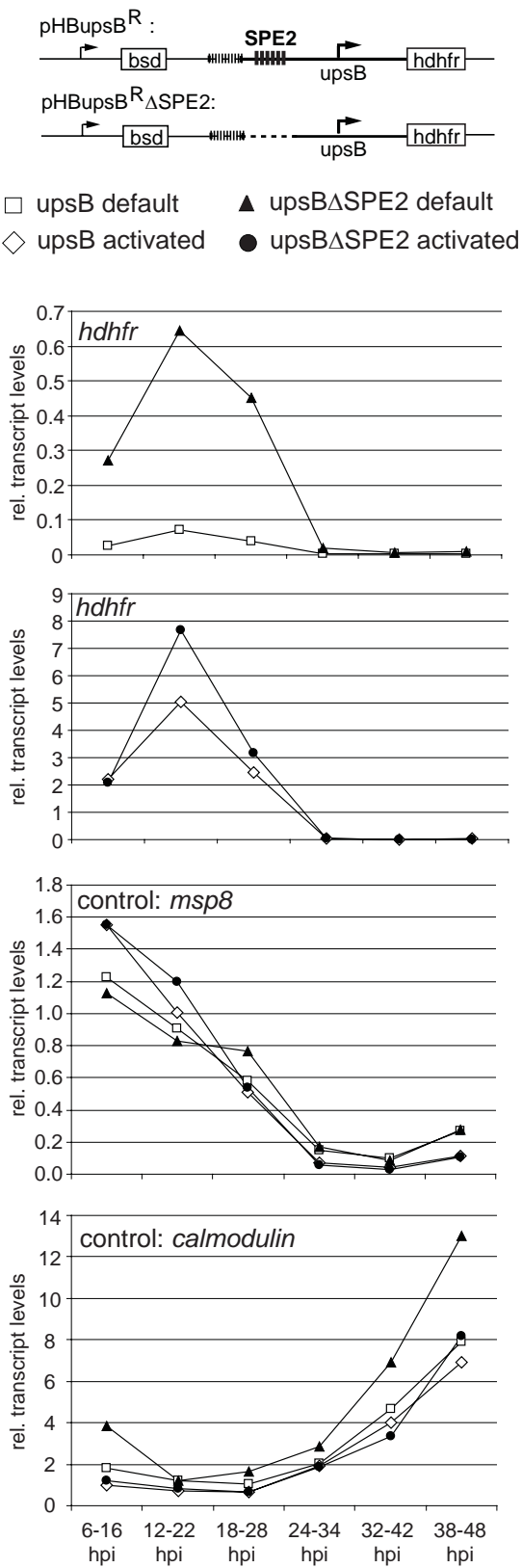

Supplement: Figure S2 — Evidence for an involvement of the PfSIP2/SPE2 interaction in var gene silencing. Reporter constructs testing the effect of a 500bp deletion including the entire SPE2 array on upsB promoter activity are depicted on top. Stage-specific promoter activity was determined by qRT-PCR in absence of WR selection (default; first graph) and after WR selection (activated; second graph). X-axis: synchronized cultures were harvested at six timepoints across the IDC. hpi: hours post-invasion. Y-axis: values represent relative transcript levels. Absolute hdhfr, msp8 and cam transcript numbers were divided by those obtained for the constitutively expressed gene PF13_0170 (www.plasmoDB.org). hdhfr transcript levels were additionally normalized for differences in plasmid copy number as determined by qPCR (Protocol S3). Transcript profiles of msp8 (ring stage-specific marker; third graph) and calmodulin (expressed in trophozoites and schizonts; last graph) show comparable stage composition for each parasite line in each timepoint. (0.02 MB PDF) [file ppat.1000784.s002.pdf]

Figure S3

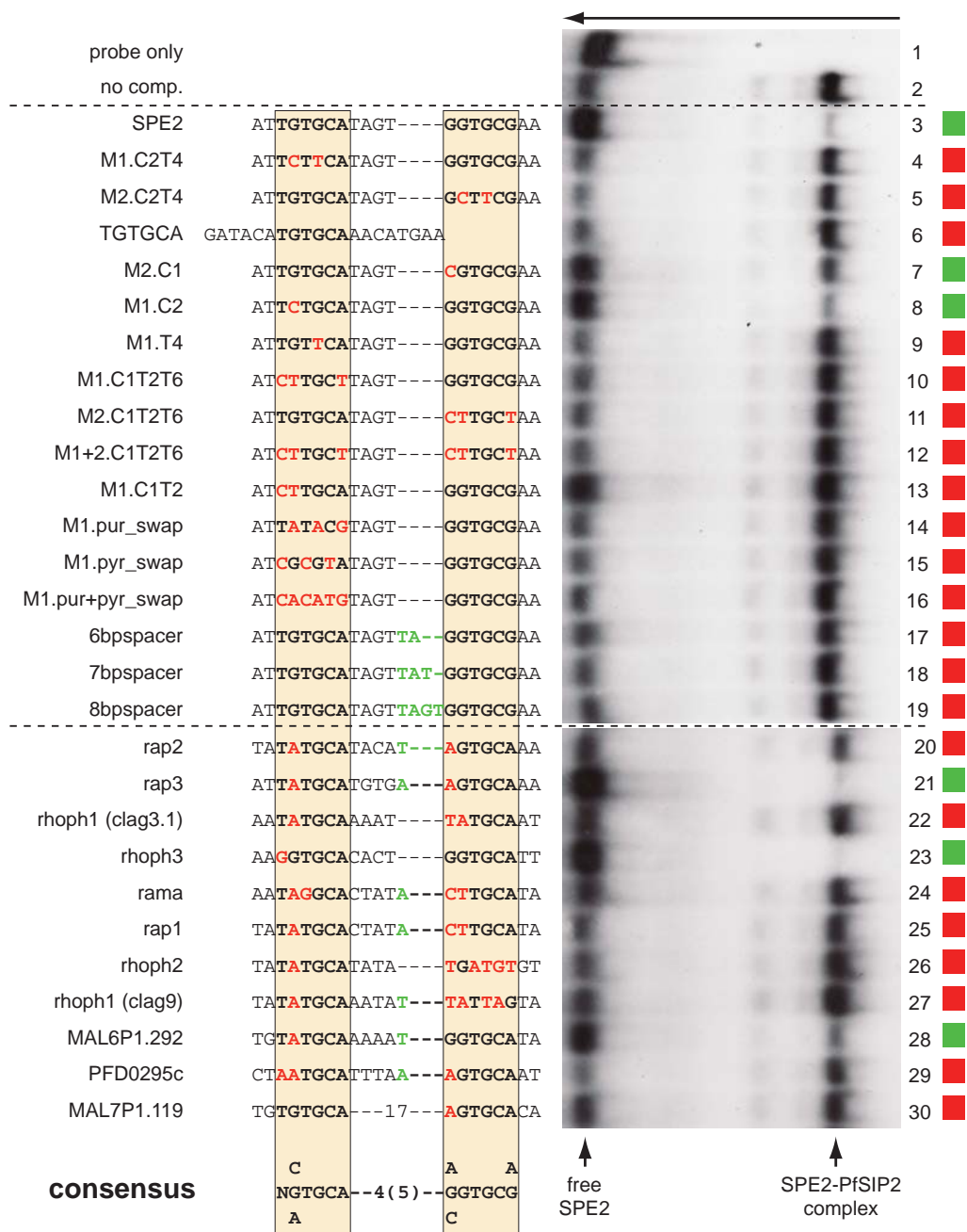

Supplement: Figure S3 — Competition EMSA to determine a functional SPE2 consensus site. Competition EMSA using recombinant PfSIP2-N-HIS_A to determine the minimal sequence requirements for binding of PfSIP2-N to a SPE2 consensus site. The gel was rotated by 90° clockwise for simpler display. Lane 1: radiolabeled 28bp SPE2 probe only; lane 2: SPE2/PfSIP2-N-HIS_A interaction in absence of competitor; lanes 3–30: SPE2/PfSIP2-N-HIS_A interaction in presence of a 100-fold molar excess of specific competitors. The names of all competitors and the original SPE2 sequence and those of all competitors are indicated to the left. The dashed lines group competitors into artificially mutated SPE2 motifs and in naturally occurring SPE2-like elements upstream of P. falciparum invasion genes. Altered nucleotides in the left or right half site of the original SPE2 sequence are highlighted in red. Additional nucleotides in the 4bp spacer are indicated in green. The arrow on top indicates the direction of electrophoretic separation. Red squares identify competitors unable to interact with PfSIP2-N_HIS_A, green squares highlight competitors that were able to compete with the SPE2/PfSIP2-N-HIS_A interaction. The experimentally determined SPE2 consensus site is shown at the bottom. (0.03 MB PDF) [file ppat.1000784.s003.pdf]

Figure S4

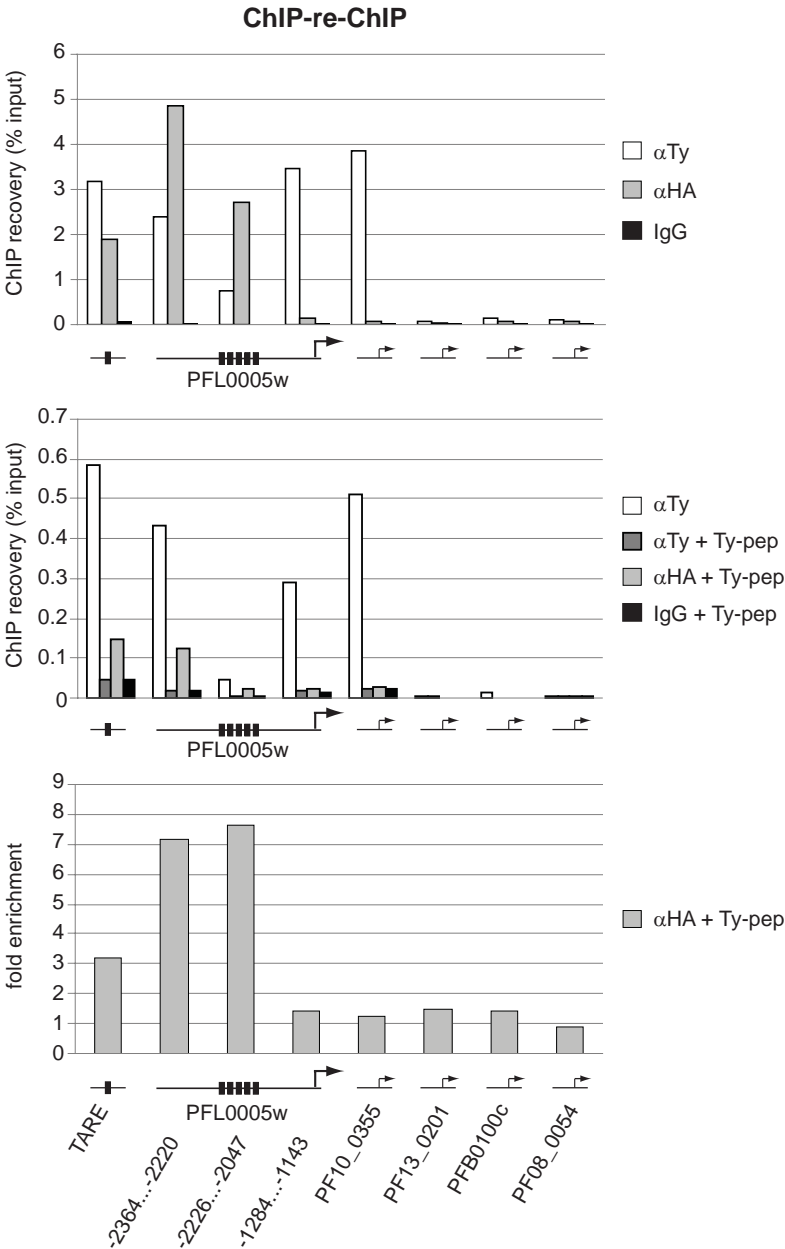

Supplement: Figure S4 — ChIP-re-ChIP shows that PfSIP2-N-HA and PfHP1-Ty co-localise in subtelomeric heterochromatin. ChIP-re-ChIP was performed on 3D7/SIP2-N-HA/HP1-Ty schizont stage parasites. Top panel: Recovery of PfSIP2-N-HA- and PfHP1-Ty-associated chromatin after the first ChIP using anti-HA and anti-Ty antibodies, respectively, compared to the negative control (rabbit IgG). Second panel: Chromatin fragments immuno-precipitated with anti-Ty antibodies in the first ChIP were used as input and were re-ChIPped using anti-HA, anti-Ty and rabbit IgG antibodies in presence of competing Ty peptide. Efficient and specific peptide competition is evident from the failure to precipitate chromatin with anti-Ty antibodies in presence, but not in absence, of Ty peptide. Re-ChIP using anti-HA antibodies shows that PfSIP2-N-HA is present on PfHP1-Ty-enriched chromatin but only at loci associated with SPE2 (indicated by thick vertical lines). Bottom panel: Results from second panel displayed as fold enrichment values for PfSIP2-N-HA on PfHP1-Ty-enriched chromatin fragments. qPCR primers were directed against upstream regions (represented by horizontal lines with arrows). This experiment was repeated using independently isolated chromatin and yielded similar results. (0.02 MB PDF) [file ppat.1000784.s004.pdf]
