# Supplementary material for: A Major Role for the Plasmodium falciparum ApiAP2 Protein PfSIP2 in Chromosome End Biology
Source: PLoS Pathog. 2010 Feb 26;6(2):e1000784. doi: 10.1371/journal.ppat.1000784 (PMC2829057; doi:10.1371/journal.ppat.1000784)
Supplement: Table S1 — SEQUEST search results of peptide tandem mass spectra for proteins eluted with SPE2. (0.08 MB PDF) [file ppat.1000784.s005.pdf]

Table S1: SEQUEST search results

SPE2 affinity purification; 2 unique peptides; orange: detected exclusively in the SPE2 sample; grey: ribosomal proteins

	Reference					P (pro)	Score	Coverage	MW	Peptide (Hits)
	Scan(s)	Peptide	MH+	DeltaM	z	P (pep)	XC	DeltaCn	Sp	Ions
1	psu|PFI1445w | organism=Plasmodium_falciparum_3D7 | product=High molecular weight rhoptry protein-2 | location=MAL9					7.67E-12	280.31		162560.6	28 (28 0 0 0 0)
	3785	K.HLINADDVSPSYINLYR.S	1990.00281	0.00127	2	7.67E-12	6.20	0.65	2897.6	28/32
	4807	R.LFVTEGTLEYLLLDK.I	1753.96228	0.00017	2	1.60E-07	5.61	0.54	2691.4	25/28
	2852	K.SLYGNNNNNNAGSESDVTLK.S	2110.96362	-0.00019	2	2.06E-10	5.55	0.54	906.3	22/38
	2591	K.GSQNTEGESEVPSDDEINK.T	2034.87341	0.00139	2	6.43E-09	5.48	0.48	1099.9	26/36
	3755	K.NNDQLTFLETQVAK.I	1620.82275	0.00017	2	1.62E-05	5.07	0.48	1680.3	21/26
	1497	K.YGAEQGVGSADSNTK.L	1483.66589	-0.00459	2	3.53E-08	4.78	0.62	1666.7	24/28
	3875	K.DFTQTNALTNLPNLDNK.K	1918.95044	0.00188	2	2.35E-10	4.41	0.52	1637.3	23/32
	3704	K.NIVSDALTSEEIK.R	1418.73730	-0.00031	2	1.88E-05	4.13	0.63	1237.1	19/24
	4607	K.SPDSLDFSIPMNFFNHK.N	1995.92688	0.00052	3	1.58E-08	4.04	0.43	1293.1	28/64
	4118	K.LFEQIVDQIK.Y	1232.68848	-0.00032	2	7.97E-04	3.80	0.17	1490.0	15/18
	4132 - 4134	R.SNNIVALYILK.T	1247.73584	-0.00068	2	5.35E-04	3.69	0.40	950.2	17/20
	3291	K.IDDINNLNIYEHK.E	1600.79651	-0.00174	3	6.70E-04	3.48	0.31	1494.2	31/48
	3968	K.YFDEYFFASGGK.I	1430.62634	0.00103	2	4.44E-05	3.40	0.57	840.9	16/22
	3896	K.YMTIYINEAYIK.N	1521.76575	0.00200	2	2.58E-04	3.37	0.51	1220.3	19/22
	3737	K.YLYMDEYLSEGDK.A	1625.70398	0.00017	2	2.99E-07	3.28	0.62	1254.6	19/24
	3980	K.YIISDDLYYVNMK.V	1636.79272	0.00017	2	9.79E-07	3.20	0.55	1044.9	18/24
	3063	K.NSQNYSLTHTEEMIK.I	1794.83264	-0.00192	3	6.45E-03	3.09	0.35	555.1	22/56
	5822 - 5824	R.FVYASYILGLVFFIESHIDIAR.L	2573.38037	-0.00030	3	1.13E-09	3.02	0.59	435.4	25/84
	4083	K.ELETILNNSPFSEEQTMK.L	2110.00098	0.00029	2	2.92E-04	2.97	0.52	319.2	15/34
	2835	K.YLLDNSNDIKK.V	1322.69507	-0.00227	2	2.91E-04	2.94	0.41	1667.1	17/20
	4065	K.VFNGIPAFLDK.N	1220.66736	-0.00191	2	5.16E-03	2.54	0.26	237.4	10/20
	3945 - 3947	K.YNFLDIYK.Q	1075.54590	-0.00087	2	3.44E-02	2.53	0.19	452.0	10/14
	3373 - 3375	K.ADSDITYFVK.Q	1158.56775	-0.00209	2	1.59E-01	2.52	0.41	681.6	13/18
	2996	R.IHPNDFLNNK.V	1211.61670	-0.00337	2	3.08E-01	2.49	0.28	123.5	9/18
	3211	K.LMDMELK.H	879.43146	-0.00118	2	8.15E-02	2.37	0.16	428.0	11/12
	3240	K.LPLEDYYK.L	1040.52991	-0.00032	2	4.75E-03	2.28	0.46	705.5	12/14
	4570	K.EGNEFLMSILHMK.S	1548.75488	0.00127	2	2.66E-05	2.11	0.56	190.7	11/24
	3312	K.LSLAPDMVK.T	973.53870	-0.00405	2	4.65E-01	2.05	0.19	234.3	8/16
2	psu|PF10_0115 | organism=Plasmodium_falciparum_3D7 | product=QF122 antigen | location=MAL10:455397-458816(+) | l					1.00E-30	190.36		131545.0	19 (19 0 0 0 0)
	2088	R.SKEEMSNNNNNNNSSSNGGAFNNSYHESR.Y	3203.30640	0.00436	3	1.00E-30	6.70	0.69	1968.0	44/112
	3432	K.LSLTNNNNMMNNNNNNNNNNNNNNNTFINSYDNK.T	3944.66553	0.00412	3	6.66E-15	6.34	0.64	1330.1	40/132
	4949	K.NLEEIEEANTYINYLEEQLSITK.N	2756.35132	0.00249	2	1.48E-12	6.03	0.74	1791.3	26/44
	4674	K.IEIENFISTINSTISR.F	1836.97021	0.00115	2	5.70E-08	5.73	0.57	1539.5	22/30
	4842	K.DDIEQFISYLQNVNFNSENK.N	2417.12549	0.00144	3	2.61E-10	5.70	0.51	1678.2	37/76
	1608	K.NNNNNNNIISNNNNNNNNNSDVKK.D	2714.22217	-0.00241	3	2.09E-05	5.13	0.53	826.8	28/92
	2812	K.TSHVAQIYGHEDNIHLAK.D	2033.01990	-0.00584	4	9.67E-11	5.07	0.63	1608.2	42/102
	3691	K.DVLENLVQSEGK.E	1330.68494	0.00041	2	1.25E-06	4.37	0.58	2061.1	19/22

	1960	K.NNNNNNNIISNNNNNNNNNSDVK.K	2586.12720	0.00498	3	7.84E-08	4.05	0.43	1418.5	36/88
	3451	K.QNVDITEADIIR.K	1386.72229	-0.00044	2	3.48E-04	4.01	0.48	1283.9	20/22
	2543	R.FNGQTTRPINMK.L	1406.72095	-0.00142	2	1.24E-03	3.94	0.37	1172.8	16/22
	3807	K.NLDSEFVPFEEK.E	1453.68457	0.00151	2	5.98E-06	3.56	0.60	1122.8	19/22
	2967	K.EIETNTNTLIR.M	1303.68518	-0.00300	2	1.39E-04	3.12	0.47	626.0	15/20
	3859	K.LTEEEAFLFNSNYR.G	1732.81775	-0.00320	3	6.40E-03	2.94	0.33	457.8	16/52
	1756	K.NSDIEEAEEK.L	1163.50623	0.00029	2	5.79E-05	2.71	0.44	684.4	15/18
	3516	K.ALEILEYSK.N	1065.58264	-0.00264	2	3.32E-03	2.68	0.40	724.1	12/16
	3003	K.YFVYVDNNK.L	1161.55750	-0.00399	2	9.82E-02	2.61	0.45	651.0	13/16
	3728	K.VIALLLSSK.A	943.61865	-0.00129	2	1.30E-03	2.47	0.30	598.3	13/16
	1899	K.LSSADMENK.C	994.45099	-0.00142	2	5.37E-03	2.21	0.12	575.2	12/16
3	psu|PFD0685c | organism=Plasmodium_falciparum_3D7 | product=chromosome associated protein, putative | location=MAI					6.84E-09	140.29		141136.4	14 (14 0 0 0 0)
	5066 - 5068	R.ENILGFLIDNINVDK.T	1716.91663	0.00108	2	1.11E-07	5.67	0.45	2746.2	24/28
	2217	K.TQDEDNHLNLSNNNK.S	1755.78918	-0.00105	2	6.84E-09	5.15	0.48	1868.2	23/28
	3157	R.IIDEEVYIDK.I	1236.63586	-0.00202	2	3.80E-04	3.83	0.37	782.8	16/18
	4222	R.LNILMNDFNELK.K	1463.75623	0.00187	2	5.23E-05	3.71	0.49	1504.7	19/22
	2803	K.ISEGTTNLNEIK.C	1318.68494	-0.00289	2	3.63E-04	3.57	0.40	1213.5	16/22
	3809	K.LSNMKDEEILNYLK.S	1709.87781	0.00021	3	3.77E-04	3.48	0.31	1067.4	25/52
	2668	K.YGSEINELNNK.S	1280.61169	-0.00288	2	7.63E-04	3.48	0.41	1247.8	18/20
	4124	K.VHLDYFLNEINYK.N	1667.84277	-0.00246	3	5.93E-03	3.24	0.29	983.7	20/48
	3917	K.NINQINEFILR.V	1373.75354	-0.00020	2	4.19E-05	3.07	0.36	919.8	15/20
	4140	K.VLLPQGIQELEEYR.T	1686.90613	0.00175	2	4.80E-06	2.97	0.54	398.8	16/26
	2444	K.KKDEALEATYVK.I	1394.75256	-0.00161	3	7.56E-03	2.73	0.40	976.0	21/44
	4455	K.LTIVPLLNIK.K	1123.74487	0.00249	2	2.85E-04	2.71	0.31	644.9	16/18
	2531	K.IRNEYDDFK.N	1199.56909	-0.00006	3	1.64E-03	2.65	0.39	987.7	19/32
	3539	K.NNEINMLLSK.N	1175.60889	0.00188	2	4.17E-02	2.56	0.38	764.0	13/18
4	psu|PFC0120w | organism=Plasmodium_falciparum_3D7 | product=Cytoadherence linked asexual protein, 3.2 | location=M/					1.14E-06	136.22		167134.0	14 (12 2 0 0 0)
	3573 - 3575	K.YGYLGEVIAAR.L	1211.64185	0.00175	2	8.08E-06	4.39	0.52	1416.1	18/20
	3165 - 3167	K.EQSIEIYNSDISDK.I	1640.76501	0.00054	2	6.39E-02	3.87	0.52	1033.5	19/26
	1422 - 1424	K.EQTQHVQEQTYER.K	1675.76709	0.00133	2	3.04E-06	3.80	0.57	738.4	17/24
	3979	K.SALEELNNVFTNK.E	1478.74854	-0.00032	2	1.14E-06	3.57	0.49	1646.9	18/24
	3468	K.HIPNNLVDELEK.L	1420.74304	0.00017	2	1.46E-05	3.32	0.45	320.6	15/22
	3784	K.TVVTNYWYPSPIK.K	1567.81555	0.00127	2	6.12E-06	3.18	0.54	834.6	17/24
	2577	K.YHNIYNINNK.L	1292.63818	-0.00056	2	4.38E-06	3.18	0.44	1018.4	16/18
	3171	K.QGTEQLIDISK.F	1231.65283	-0.00154	2	7.91E-03	2.79	0.34	384.2	15/20
	3492	K.ILEFSMSDR.F	1097.52954	-0.00215	2	1.53E-04	2.73	0.49	635.0	13/16
	3367	K.DSNLLNNITK.C	1131.60046	-0.00190	2	4.40E-04	2.66	0.40	859.5	14/18
	3773 - 3775	R.SYPLSLVLEHK.F	1285.71509	-0.00203	2	4.85E-03	2.55	0.53	160.1	11/20
	2905	R.RYDMENAFK.N	1173.53577	-0.00215	2	9.83E-05	2.52	0.42	1118.3	14/16
	2641	R.EIEIHNSMASR.Y	1286.61572	-0.00056	2	4.35E-03	2.13	0.30	406.8	16/20
	3352	K.TTQDTDFDLHGMMEHK.Y	1905.81055	-0.00279	4	4.22E-01	2.05	0.13	337.0	22/90
5	gi|81175178|sp|P35527|K1C9_HUMAN Keratin, type I cytoskeletal 9 (Cytokeratin-9) (CK-9) (Keratin-9) (K9)					1.71E-13	130.34		62091.8	13 (13 0 0 0 0)

	1322	R.GSRGGSGGSYGGGGSGGGYGGGSGSR.G	2091.88232	0.00443	3	9.47E-10	6.87	0.56	3083.2	45/100
	1349	R.GGSGGSYGGGGSGGGYGGGSGSR.G	1791.72766	0.00444	2	1.71E-13	5.80	0.77	2133.3	28/44
	3767	R.GGGGSFGYSYGGGSGGGFSASSLGGGFGGGSR.G	2705.16113	-0.00051	3	1.07E-07	5.76	0.67	1399.3	39/124
	2916	K.VQALEEANNDLENK.I	1586.76562	0.00030	2	6.99E-06	5.23	0.39	2830.0	23/26
	1373	R.GGSGGSYGRGSRGGSGGSYGGGGSGGGYGGGSGSR.G	2870.21802	0.00283	4	4.53E-06	4.70	0.56	2138.9	55/204
	3837	K.TLNDMRQEYEQLIAK.N	1851.92688	0.00359	2	1.63E-05	3.92	0.36	1632.7	20/28
	4661	K.SDLEMQYETLQEELMALK.K	2171.02466	-0.00192	3	1.14E-06	3.64	0.45	808.8	24/68
	1891	R.SGGGGGGGLGSGGSIR.S	1232.59778	-0.00117	2	4.41E-06	3.48	0.48	926.5	18/30
	3851	R.LASYLDKVQALEEANNDLENK.I	2377.18823	0.00394	3	8.37E-02	3.42	0.37	710.9	26/80
	1525	R.FSSSSGYGGGSSR.V	1235.52869	-0.00289	2	3.37E-07	3.03	0.64	1461.0	19/24
	2357	K.IGLGGRGGSGGSYGR.G	1350.68726	0.00187	2	1.59E-02	2.95	0.38	585.6	17/28
	3177	R.IKFEMEQNLR.Q	1307.67761	-0.00118	3	7.56E-05	2.71	0.25	812.5	19/36
	2980	R.QGVDADINGLR.Q	1157.59094	-0.00398	2	1.91E-03	2.56	0.35	438.4	13/20
6	psu|PF11_0317 | organism=Plasmodium_falciparum_3D7 | product=structural maintenance of chromosome protein, putative					4.12E-09	120.28		214541.8	12 (12 0 0 0 0)
	2303	K.NMENDKDINNEKENMEK.E	2094.90674	0.01169	3	4.12E-09	5.62	0.43	2097.6	34/64
	2723	K.MNSHIDQQDQNESNVHLNNENIK.K	2721.22803	-0.00181	4	3.54E-06	5.12	0.41	1777.6	45/132
	3713	K.EESLQFEQDILNQNK.K	1834.88171	0.00017	2	5.19E-06	4.34	0.52	1557.7	19/28
	3608	K.NLNNLNQELNQLR.D	1582.82959	0.00212	2	3.89E-05	3.96	0.59	1777.6	20/24
	3697	K.NNNLIIDITNR.Y	1299.70154	0.00127	2	8.90E-04	3.60	0.34	1314.5	17/20
	2772	K.LAYDQLNER.I	1121.55859	-0.00276	2	5.22E-05	3.14	0.42	1031.5	15/16
	3481	K.KFELLDTYEK.E	1285.66748	0.00005	2	1.01E-04	2.97	0.39	1277.3	16/18
	4495 - 4497	R.MDFIPYENFVSNLK.K	1716.83020	0.00194	2	2.85E-05	2.94	0.50	465.4	18/26
	2181	K.ESNEQIDNIQK.I	1317.62805	-0.00251	2	1.90E-03	2.76	0.32	899.4	15/20
	3793	K.FMENLNLQIK.E	1249.66089	-0.00093	2	9.01E-04	2.61	0.35	763.2	15/18
	1725	K.VMENYEQK.I	1040.47168	-0.00374	2	1.64E-03	2.26	0.30	469.9	12/14
	2413	K.NEESINEENIK.K	1318.61206	-0.00215	2	5.66E-03	2.17	0.14	1157.1	17/20
7	gi|11935049|gb|AAG41947.1|AF304164_1 keratin 1 [Homo sapiens]					3.31E-13	110.33		66027.0	11 (11 0 0 0 0)
	3349	K.SKAEAESLYQSKYEELQITAGR.H	2501.25171	-0.00229	3	7.99E-09	6.68	0.60	2262.4	40/84
	1828	R.GGGGGGYGSGGSSYGSGGGSYGSGGGGGGGR.G	2383.95190	0.00493	2	3.31E-13	6.17	0.75	1402.6	27/60
	5042	R.SLDLDSIIAEVKAQYEDIAQK.S	2349.21851	0.00151	2	1.14E-10	5.50	0.63	1863.6	25/40
	3439	K.LNDLEDALQQAK.E	1357.69580	-0.00081	2	8.28E-07	5.11	0.38	2311.5	20/22
	2048	K.SKAEAESLYQSK.Y	1340.66919	-0.00166	2	6.31E-07	4.76	0.46	1425.7	19/22
	2789	R.TNAENEFVTIKK.D	1393.73218	-0.00313	2	1.60E-05	3.75	0.56	1614.2	18/22
	4434	R.SLDLDSIIAEVK.A	1302.71509	0.00127	2	4.21E-04	3.47	0.28	1138.3	16/22
	3169	K.NMQDMVEDYR.N	1300.52966	-0.00202	2	3.94E-03	3.29	0.57	1118.3	14/18
	2211	K.AQYEDIAQK.S	1065.52112	0.01384	2	8.03E-03	3.22	0.35	500.5	13/16
	1713	R.LRSEIDNVKK.Q	1201.68994	-0.00384	3	2.99E-01	3.08	0.19	1179.9	22/36
	2948	R.DYQELMNTK.L	1141.51941	-0.00313	2	8.04E-03	2.07	0.46	723.9	13/16
8	psu|PFI0265c | organism=Plasmodium_falciparum_3D7 | product=RhopH3 | location=MAL9:270738-274787(-) | length=897					9.64E-10	110.31		104789.1	11 (11 0 0 0 0)
	3216	R.KQDVLYETDKPQTMDEASYEETVDEDAHHVNEK.Q	3893.72949	0.00202	5	9.64E-10	5.96	0.65	2196.3	57/256
	3281 - 3283	K.TKDQDLEIELYK.Y	1494.76868	0.00157	2	2.39E-06	4.61	0.47	1521.4	19/22
	4021	K.NYLDSVQNLDTECFK.K	1788.81091	0.00127	2	4.38E-08	4.28	0.65	1274.2	21/28

	2361	K.EDNSEIQCQNVR.K	1434.62781	0.00334	2	1.84E-04	3.62	0.49	1177.7	18/22
	3597 - 3599	K.ELSHNITDFSFK.E	1437.70093	0.00291	2	1.24E-04	3.54	0.50	682.9	17/22
	4308 - 4310	R.TMYLDFESSDIFSR.E	1710.76794	0.00298	2	1.28E-08	3.20	0.63	770.2	18/26
	2143	K.YQLNKEEYK.V	1214.60522	-0.00019	2	4.01E-04	3.08	0.33	979.8	14/16
	4239	R.LFFTYNFGDVEPQGK.Y	1761.84827	0.00261	2	1.32E-07	2.93	0.41	584.6	15/28
	3408	K.ASLHVPSVLYR.R	1241.70007	-0.00121	3	1.20E-01	2.62	0.34	713.6	19/40
	4138	R.SWVSEFLK.E	995.51965	-0.00093	2	3.92E-02	2.61	0.12	337.5	10/14
	2652	R.YTSLYIHK.F	1024.54626	-0.00361	2	1.49E-02	2.47	0.42	466.2	11/14
9	psu|PFF0835w | organism=Plasmodium_falciparum_3D7 | product=hypothetical protein, conserved | location=MAL6:717047					7.16E-08	110.30		52947.8	11 (11 0 0 0 0)
	3335	K.IENEASKYDDETIVPLK.S	1963.98584	-0.00265	3	7.16E-08	6.03	0.57	1690.0	33/64
	5198	R.EELALIENMLAYLK.K	1649.88184	0.00078	2	9.21E-07	5.02	0.45	2433.9	23/26
	3719	K.LLINQIQALNK.N	1267.77319	-0.00044	2	2.28E-05	4.62	0.44	1651.9	18/20
	2289 - 2291	K.LKELQNSYQEK.N	1379.71655	0.01128	2	1.09E-04	3.69	0.36	1081.4	16/20
	3609	K.KLEDIDVLPYR.E	1360.74707	0.00139	2	8.68E-05	3.63	0.46	1687.6	16/20
	3079	K.QIGFENEEDIEK.K	1450.66968	-0.00069	2	4.11E-03	3.62	0.49	1159.4	17/22
	3152	K.LGSISQSIDEIKK.K	1417.78967	0.00078	2	3.70E-07	3.62	0.36	819.9	17/24
	3363	K.LGSISQSIDEIK.K	1289.69470	-0.00325	2	1.45E-02	3.38	0.48	1192.0	19/22
	2911	K.IEKPDFDAYNEK.L	1468.69543	-0.00105	2	6.65E-05	2.89	0.31	429.9	15/22
	3313	K.YDDETIVPLK.S	1192.60962	-0.00264	2	6.48E-04	2.82	0.52	1033.9	14/18
	2568	R.RSITVEMEEK.K	1221.61438	-0.00056	2	6.29E-04	2.77	0.34	897.8	16/18
10	psu|PF14_0316 | organism=Plasmodium_falciparum_3D7 | product=DNA topoisomerase II, putative | location=MAL14:1332					1.79E-08	110.24		169121.8	11 (11 0 0 0 0)
	1948	K.NNNNNNNNNDEDEIVK.I	1873.79065	0.00554	2	1.79E-08	4.83	0.39	1231.2	24/30
	4779	K.SPILSNILLWAQAK.A	1553.90503	0.00261	2	6.05E-07	4.02	0.43	1657.3	18/26
	3995	K.STLTTTNMTLFDPNLK.L	1796.90991	0.00066	2	3.72E-07	3.61	0.63	1500.5	20/30
	4444 - 4446	K.GFLSEFVTPIVK.V	1336.75110	-0.00301	2	5.07E-05	3.09	0.58	1015.7	16/22
	3399	R.ISVYNDGEGIPVDIHK.E	1755.89124	-0.00229	3	1.58E-02	3.04	0.41	463.4	23/60
	4288 - 4290	K.EFLEELLTDEK.H	1365.67834	-0.00037	2	8.84E-04	2.86	0.37	741.4	12/20
	3632	R.YGSLMIMTDQDYDGSHIK.G	2073.92554	-0.00052	3	4.03E-02	2.78	0.42	482.8	21/68
	3663	K.DLSYYDFVNK.E	1263.58923	-0.00080	2	7.09E-04	2.75	0.48	971.4	15/18
	4157	K.IMFLWTGDR.D	1138.57141	-0.00081	2	2.66E-04	2.61	0.37	741.0	13/16
	2756	K.VTFKPDLNK.F	1061.59900	-0.00337	2	3.68E-02	2.59	0.28	332.9	10/16
	3551	R.IMGLDITDK.N	1005.52850	0.00274	2	3.36E-03	2.34	0.29	473.8	14/16
11	psu|PFF1185w | organism=Plasmodium_falciparum_3D7 | product=iswi protein homologue | location=MAL6:999525-100768					6.26E-12	100.37		315421.1	10 (10 0 0 0 0)
	3916	R.RPLVDNVQLKPHQEDGVEWLLK.S	2613.41479	0.00112	4	6.26E-12	7.31	0.51	4347.0	53/126
	4392	K.IEALENSPLFMEIYNK.G	1910.95679	0.00358	2	1.80E-08	5.39	0.62	2069.4	23/30
	2045	K.GIEYNKNDDEDYNNNESK.D	2160.89526	0.04404	3	3.57E-07	4.66	0.51	1870.5	32/68
	3252	R.IDALDQQYEDQLR.K	1606.77075	0.00054	2	2.84E-06	4.49	0.52	2093.3	19/24
	2341	K.INEDSTVGVTDK.G	1277.62195	0.00053	2	3.26E-06	4.31	0.52	1814.0	20/22
	4804	K.AAIQNMDVESTNNFPLEFFIER.G	2585.23413	0.00229	3	1.68E-09	4.22	0.54	950.9	33/84
	1717	K.NISGEEHIESSSK.L	1416.66016	-0.00313	2	1.17E-07	4.01	0.60	1256.5	20/24
	3360	K.GVGTTFLSDGSMR.F	1327.63110	-0.00202	2	2.41E-05	3.94	0.54	1603.7	19/24
	3580	K.VNVNIGNIDLK.S	1198.67896	0.00139	2	1.09E-02	2.99	0.35	879.0	15/20

	3689	K.AYENVFPNNFVK.E	1441.71106	-0.00142	2	2.31E-02	2.28	0.31	269.9	12/22
12	psu|PF13_0305 | organism=Plasmodium_falciparum_3D7 | product=elongation factor 1 alpha | location=MAL13:2265234-22					1.03E-12	100.32		48927.9	10 (10 0 0 0 0)
	4348 - 4350	K.VGYQADKVDFIPISGFEGDNLIEK.S	2654.33472	0.00131	3	1.03E-12	6.40	0.51	1691.9	37/92
	3401	K.EVLEEARPGDNIGFNVK.N	1886.96069	0.00005	2	3.10E-07	5.55	0.55	961.9	19/32
	4852	K.NMITGTSQADVALLVVPAEVGGFEGAFSK.E	2908.47607	0.00138	3	4.01E-11	5.06	0.64	1420.8	39/112
	4418	K.VDFIPISGFEGDNLIEK.S	1892.96399	-0.00020	2	4.38E-07	5.03	0.52	1089.0	25/32
	3649	K.FTAQVIILNHPGEIK.N	1679.94788	0.00017	2	7.22E-09	4.95	0.49	2321.3	23/28
	3791	K.KPMVVETFTEYPPLGR.F	1863.96729	0.00065	2	6.41E-09	4.24	0.66	1622.7	22/30
	3739	R.YFFTVIDAPGHK.D	1394.71033	-0.00085	3	4.85E-06	3.84	0.46	879.9	26/44
	3227	K.SGDSALVSLEPK.K	1202.62634	0.00127	2	2.42E-04	3.23	0.50	1627.2	18/22
	1896	K.YSEDRYEEIKK.E	1459.70630	0.00957	2	1.95E-02	2.44	0.17	463.0	12/20
	3015	K.FLNIDSK.I	836.45123	-0.00025	2	1.69E-01	2.40	0.14	510.1	10/12
13	psu|MAL7P1.229 | organism=Plasmodium_falciparum_3D7 | product=Cytoadherence linked asexual protein | location=MAL					4.81E-09	100.21		162677.5	10 (10 0 0 0 0)
	3692	R.NILNVVDEVNK.S	1256.68445	0.00005	2	1.02E-04	3.59	0.50	1227.1	17/20
	5239	K.NVDDILFFADIINIR.K	1777.94836	0.00103	2	1.33E-06	3.48	0.47	1904.8	19/28
	3295	R.AHPLSLILEHK.F	1257.73132	-0.00112	3	2.86E-03	3.40	0.19	618.4	23/40
	4464	K.HLPNNLLDELLR.Q	1446.80640	-0.00020	2	3.69E-07	3.11	0.57	329.2	15/22
	3001	K.GEAIILGDTK.I	1016.56226	-0.00276	2	9.92E-03	3.03	0.38	1207.2	14/18
	4645	K.SINDADDDIYIVPTIQSSFYDIAK.Y	2703.30347	0.00174	3	3.46E-04	3.01	0.39	365.2	22/92
	3883	K.EQSIESYNANISDLIK.K	1823.90210	0.00078	2	4.81E-09	2.99	0.63	919.2	20/30
	3032	K.IALEELNK.V	929.53021	-0.00196	2	8.41E-03	2.73	0.20	530.9	12/14
	3256	R.GYLDEAMEAR.I	1154.51465	0.00005	2	2.83E-03	2.68	0.47	832.9	13/18
	2545	R.LLNSHSETFR.N	1203.61169	-0.00251	2	2.09E-05	2.46	0.41	907.7	15/18
14	gi|40354192|ref|NP_000412.2| keratin 10 [Homo sapiens]					1.88E-10	90.27		58791.5	9 (9 0 0 0 0)
	3463	K.GSLGGGFSSGGFSGGSFSR.G	1707.77209	0.00164	2	1.88E-10	5.42	0.66	1511.5	23/36
	2799	R.ALEESNYELEGK.I	1381.64819	-0.00312	2	3.59E-06	4.20	0.61	2051.2	19/22
	2648	R.LENEIQTYR.S	1165.58484	-0.00471	2	7.77E-04	4.17	0.13	1226.0	15/16
	2257	R.SQYEQLAEQNRK.D	1493.73425	0.03045	2	8.68E-04	3.87	0.42	1228.2	16/22
	4694	R.NVSTGDVNVEMNAAPGVDLTQLLNNMR.S	2872.39282	0.00333	3	5.96E-07	3.66	0.48	888.2	30/104
	2475	R.SLLEGEGSSGGGGR.G	1262.59717	-0.00179	2	2.14E-06	3.60	0.44	1331.2	21/26
	2476	R.SQYEQLAEQNR.K	1365.63928	-0.00129	2	1.83E-04	3.41	0.49	1423.6	16/20
	3105	K.QSLEASLAETEGR.Y	1390.68091	-0.00081	2	1.45E-06	3.08	0.58	576.6	17/24
	3577	R.SQYEQLAEQNRKDAEAWFNEK.S	2584.20630	0.00429	4	2.03E-03	1.96	0.37	254.6	27/120
15	psu|PF14_0359 | organism=Plasmodium_falciparum_3D7 | product=hypothetical protein, conserved | location=MAL14:1533					6.66E-15	80.39		48438.4	8 (8 0 0 0 0)
	5056	R.KLYDEYGEEGLENGEQPADATDLFDFILNAGK.G	3561.65430	0.01108	3	6.66E-15	7.88	0.75	2438.7	44/124
	2483	K.QQQQQQQEAYDDEDHQPEMEGGR.V	2774.13428	-0.08151	3	9.04E-08	4.70	0.67	456.4	21/88
	5135	K.LYDEYGEEGLENGEQPADATDLFDFILNAGK.G	3433.55933	0.01713	2	6.37E-13	4.31	0.76	598.9	19/60
	3587	K.VTLEQLYNGATK.K	1336.71069	0.00188	2	1.18E-04	3.83	0.43	829.0	15/22
	2440	K.KRGEDIVSEVK.V	1259.69543	-0.00042	3	2.40E-06	3.58	0.37	1778.5	26/40
	3119	R.EVLDEGMPTYK.D	1281.60315	-0.00142	2	5.61E-02	2.86	0.43	399.5	11/20
	2645	K.RGEDIVSEVK.V	1131.60046	-0.00178	2	3.14E-03	2.77	0.29	674.9	14/18
	3284	R.KIIEVYIPK.G	1102.68701	-0.00215	2	7.58E-03	2.32	0.23	596.1	12/16

16	gi|14277739|pdb|1HYN|P Chain P, Crystal Structure Of The Cytoplasmic Domain Of Human Erythrocyte Band-3 Protein					1.48E-08	80.29		42509.3	8 (8 0 0 0 0)
	3351	K.HSHAGELEALGGVKPAVLTR.S	2042.11414	-0.00255	4	2.81E-08	5.70	0.52	2442.2	51/114
	2612	R.YQSSPAKPDSSFYK.G	1604.75916	0.00309	2	1.94E-02	4.31	0.56	1745.5	21/26
	3684	K.IPPDSEATLVLVGR.A	1466.82129	-0.00081	2	1.48E-08	3.97	0.56	745.2	22/26
	4922	K.GTVLLDLQETSLAGVANQLLDR.F	2326.26123	0.00200	2	4.31E-06	3.44	0.59	597.7	18/42
	4278	R.LQEAAELEAVELPVPIR.F	1877.03784	0.00298	2	3.27E-06	3.17	0.52	691.7	16/32
	4163	K.VYVELQELVMDEK.N	1594.80334	0.00298	2	7.26E-08	3.06	0.48	1198.5	19/24
	4382	R.ADFLEQPVLGFVR.L	1490.80017	-0.00007	2	2.90E-06	2.92	0.51	793.8	15/24
	2485	R.IDAYMAQSR.G	1054.49866	-0.00252	2	1.31E-03	2.51	0.44	1126.7	14/16
17	psu|PF14_0368 | organism=Plasmodium_falciparum_3D7 | product=2-Cys peroxiredoxin | location=MAL14:1575927-15765					1.31E-08	80.28		21793.3	8 (8 0 0 0 0)
	3841	K.GGIGNIQHTLISDITK.S	1666.91223	0.00103	2	1.31E-08	5.59	0.57	2239.7	23/30
	4370	K.AEAVFADNTFGEVNLHDFIGK.K	2294.10889	0.00082	3	5.40E-04	3.64	0.50	694.3	31/80
	3963	R.SYNVLFGDSVSLR.A	1456.74304	0.00200	2	5.95E-06	3.32	0.43	953.4	17/24
	3832	K.QGVVQHLLVNNLAIGR.S	1731.00244	0.00237	2	1.20E-05	3.09	0.46	650.0	18/30
	3329	R.NVELIGCSVDSK.Y	1263.62488	-0.00240	2	4.50E-05	3.08	0.36	1000.4	17/22
	3135	K.VAMKPSEEGVSEYLSK.L	1753.86768	-0.00443	3	3.69E-02	3.00	0.31	981.9	26/60
	3311	R.AFVLIDK.Q	805.48181	-0.00087	2	4.04E-02	2.37	0.27	428.6	10/12
	2703	R.SVEEVLR.I	831.45703	-0.00013	2	2.86E-01	2.29	0.15	364.2	10/12
18	psu|PF14_0567 | organism=Plasmodium_falciparum_3D7 | product=hypothetical protein | location=MAL14:2433297-243431					5.71E-08	80.21		40043.0	8 (8 0 0 0 0)
	2992	K.KPKPISVALLNNK.K	1421.88391	-0.00323	3	9.35E-05	4.16	0.28	1999.5	29/48
	4213	K.NIQYNYINDLFVTNK.Y	1858.93335	0.00285	2	5.71E-08	3.93	0.64	1403.8	19/28
	1471	K.EKEDDHSSSNVTNEVK.K	1817.81482	-0.00044	2	9.06E-03	3.59	0.53	535.4	17/30
	4197	K.YFNISPLIIDNK.R	1436.77844	-0.00103	3	1.10E-03	3.47	0.42	890.5	22/44
	2599	K.NTSIHPVINASR.S	1308.70190	-0.00117	2	3.06E-04	3.21	0.42	630.4	17/22
	2351	K.YIDVSNTEK.L	1068.52075	0.00042	2	2.94E-04	2.74	0.36	777.6	14/16
	1622	K.EDDHSSSNVTNEVK.K	1560.67725	-0.00130	2	2.70E-05	2.27	0.37	226.6	14/26
	2561 - 2563	K.LSEPPQIK.Y	911.51965	-0.00102	2	7.84E-02	2.18	0.29	450.4	11/14
19	psu|PF11_0099 | organism=Plasmodium_falciparum_3D7 | product=heat shock protein DnaJ homologue Pfj2 | location=MA					1.15E-12	70.32		62337.2	7 (7 0 0 0 0)
	5049	K.TPSLLLVDDIDSLSGDLTQLK.N	2243.20166	0.00322	2	1.15E-12	6.41	0.69	2891.1	28/40
	3685	K.FVDNVVSGGIPINQNIK.R	1813.98071	-0.00044	2	9.77E-07	3.70	0.43	624.8	21/32
	3633 - 3635	R.TDENLTYFIK.N	1243.62048	-0.00025	2	1.58E-03	3.50	0.43	1437.0	16/18
	3379	K.FVHVEQYDDEL.-	1393.62708	-0.00081	2	1.35E-04	2.92	0.48	764.8	17/20
	2740	K.TYETFYGNR.T	1150.51636	-0.00069	2	3.27E-02	2.21	0.20	445.9	11/16
	4447	K.NFDFNILSLK.L	1210.64661	-0.00325	2	2.88E-02	2.15	0.38	487.4	12/18
	2288	K.SFDEDIKK.V	981.48877	0.00853	2	4.07E-01	2.10	0.21	492.8	11/14
20	psu|PF14_0102 | organism=Plasmodium_falciparum_3D7 | product=rhoptry-associated protein 1, RAP1 | location=MAL14:4					7.79E-07	70.24		89996.4	7 (7 0 0 0 0)
	3076	K.TDMLSLQNEESK.I	1394.64673	-0.00056	2	3.70E-03	4.57	0.39	1357.1	19/22
	3977	K.YSLNNMEENINILK.N	1694.84180	0.00065	2	7.79E-07	4.15	0.33	1458.9	20/26
	3901	K.FHPNIDYLTLADGYK.L	1766.87476	-0.00131	3	6.07E-03	3.81	0.36	1212.7	27/56
	3823	K.TFSGIGFNLTEK.E	1313.67358	0.00115	2	1.55E-05	3.33	0.45	761.5	16/22
	4298	R.TMVTIINDYFEAK.K	1544.76648	-0.00069	2	7.22E-06	3.28	0.55	1078.2	18/24
	3073	K.LHVITSGLSYK.A	1217.68884	-0.00239	2	2.25E-05	3.28	0.41	980.6	17/20

	3155	R.ESMISTTFEQQK.E	1428.66748	-0.00007	2	1.28E-04	3.26	0.31	959.0	17/22
21	psu|PFF0200c | organism=Plasmodium_falciparum_3D7 | product=hypothetical protein, conserved | location=MAL6:171298					5.23E-07	70.24		229481.9	7 (7 0 0 0 0)
	3913	K.SGITFEQLYPDKGPPIVR.V	2017.07532	0.00041	2	7.00E-07	4.73	0.60	1154.7	21/34
	3327	R.EHIGSQEPVILIDK.I	1577.85339	-0.00068	2	5.23E-07	4.42	0.55	1863.9	21/26
	4025 - 4027	R.VFENVGVYNVSLIR.D	1608.87439	0.00170	2	2.02E-06	3.77	0.51	1591.8	21/26
	2972	K.RADTFAQSMIK.G	1267.64636	-0.00178	2	1.73E-05	3.71	0.34	1238.9	17/20
	3811	K.GIFNPILLHK.A	1151.69348	0.00029	2	2.84E-04	3.22	0.48	846.6	15/18
	3127	R.ADTFAQSMIK.G	1111.54517	-0.00472	2	2.50E-02	3.10	0.35	776.1	14/18
	3687	K.SGITFEQLYPDK.G	1397.69470	0.00017	2	1.89E-04	2.84	0.28	1497.4	18/22
22	gi|112798|sp|P16452|EPB42_HUMAN Erythrocyte membrane protein band 4.2 (Erythrocyte protein 4.2) (P4.2)					3.42E-12	70.23		76924.7	7 (7 0 0 0 0)
	3259	R.VLPTPQTQATQEGALLNK.R	1909.03894	0.00029	2	3.42E-12	4.44	0.59	709.8	23/34
	2368	K.VALTAQTGEQPSK.I	1329.70093	0.00078	2	5.93E-06	3.74	0.52	1708.7	18/24
	2659	R.VVTTFASAQGTGGR.L	1351.69641	-0.00227	2	1.02E-04	3.02	0.31	775.1	20/26
	3496	K.WWSAVVEER.D	1161.56873	-0.00045	2	2.81E-03	2.71	0.42	894.6	14/16
	2603	K.WSQPVHVAR.V	1079.57446	-0.00068	2	4.16E-04	2.65	0.28	643.7	15/16
	2876	R.NPPENTFLR.L	1087.55310	-0.00337	2	5.96E-04	2.47	0.31	566.4	12/16
	2024	K.YPEGSLQEK.E	1050.51025	-0.00154	2	8.64E-02	2.05	0.23	320.3	11/16
23	psu|PF08_0032 | organism=Plasmodium_falciparum_3D7 | product=DnaJ protein, putative | location=MAL8:1044885-10468					1.19E-07	70.22		76633.7	7 (7 0 0 0 0)
	1626	R.KTYQNINSSYNK.F	1459.71753	0.00273	2	4.97E-06	4.00	0.49	1214.2	16/22
	3111	K.YNEIDNISNESIK.K	1538.73328	0.00126	2	1.19E-07	3.84	0.55	2008.1	22/24
	1999	K.TYQNINSSYNK.F	1331.62268	-0.00483	2	2.10E-05	3.54	0.32	1545.1	17/20
	4303	K.NGSEETEEQLFIQPVYVLK.K	2223.11792	0.00071	3	2.16E-03	3.35	0.30	617.1	22/72
	4008	K.ENQNIDYFILVNNNQVK.I	2065.03491	-0.00190	2	3.82E-04	2.63	0.55	377.1	12/32
	2960	K.MLNDALYK.M	967.49170	-0.00270	2	7.06E-04	2.52	0.20	379.2	11/14
	3888	K.DIEILNFQK.I	1119.60449	-0.00227	2	4.18E-03	2.44	0.18	945.3	12/16
24	psu|PFB0895c | organism=Plasmodium_falciparum_3D7 | product=replication factor C subunit 1, putative | location=MAL2:7					1.20E-08	70.21		104119.9	7 (7 0 0 0 0)
	4336 - 4338	K.NIQSLANPFEITLK.L	1587.87402	0.00078	2	1.89E-06	4.16	0.54	1213.2	20/26
	3353	K.NLNELVGNNQNVIK.L	1568.83911	0.00042	2	6.52E-08	3.54	0.58	1548.7	21/26
	2580	K.AFELQQQNK.S	1105.56360	-0.00191	2	4.90E-03	3.24	0.33	1386.1	14/16
	2383	K.LLPQTDQTQENDK.T	1529.74414	-0.00105	2	6.29E-08	3.01	0.48	428.5	20/24
	4928	K.WNVFSQIAHDLSLADK.I	1843.93372	-0.00089	3	6.48E-05	2.78	0.47	382.1	20/60
	2684	R.LPNQENLYDK.L	1233.61096	-0.00166	2	7.07E-03	2.75	0.29	575.8	15/18
	3133	K.TNYLVHGEYLEDGR.L	1665.78674	0.00029	2	1.20E-08	2.17	0.29	788.1	19/26
25	gi|27574029|pdb|1N11|A Chain A, D34 Region Of Human Ankyrin-R And Linker [MASS=4					2.98E-09	60.31		46366.4	6 (6 0 0 0 0)
	3611	K.LLLENNANPNLATTAGHTPLHIAAR.E	2622.41113	0.00210	4	2.98E-09	6.21	0.56	3300.1	53/144
	4291	K.SGLTPLHLVAQEGHVPVADVLIK.H	2393.35522	0.00242	3	2.47E-08	5.66	0.62	1169.9	35/88
	3149 - 3151	K.NGASPNEVSSDGTTPLAIAK.R	1928.95593	0.00097	2	3.48E-09	4.53	0.64	1184.8	24/38
	4059	R.LGYISVTDVLK.V	1207.69324	-0.00118	2	1.82E-04	3.54	0.21	956.6	17/20
	2247	K.HGVMVDATTR.M	1086.53601	0.02397	2	4.17E-06	3.20	0.57	1507.3	17/18
	3083	R.MGYTPLHVASHYGNIK.L	1787.88977	-0.00205	4	3.42E-03	2.77	0.50	937.0	37/90
26	psu|MAL8P1.105 | organism=Plasmodium_falciparum_3D7 | product=hypothetical protein, conserved | location=MAL8:5618					2.43E-10	60.27		133674.9	6 (6 0 0 0 0)
	3925	K.TYSINLNNENVELFSK.S	1884.93384	0.00127	2	2.43E-10	4.52	0.62	1935.5	23/30

	4975	K.VVNLFDEENIVINETNYIIDHNVK.S	2844.44141	-0.00133	4	4.41E-08	4.27	0.31	863.1	39/138
	3984	K.SNFEFMNSLVK.N	1315.63513	0.00127	2	2.80E-03	2.76	0.50	1091.0	16/20
	4955 - 4957	K.DSHILIFDVLNGDIIYEK.K	2104.09619	0.00132	3	4.59E-04	2.65	0.39	801.3	22/68
	3643	K.IAYLPFSNDK.N	1167.60449	-0.00178	2	1.06E-03	2.32	0.40	728.2	14/18
	3203	K.NQVIGYYLNK.E	1211.64185	-0.00362	2	2.83E-01	2.20	0.32	605.6	14/18
27	psu|PFI0165c | organism=Plasmodium_falciparum_3D7 | product=DEAD/DEAH box helicase, putative | location=MAL9:151					2.64E-09	60.27		300733.3	6 (6 0 0 0 0)
	4551	R.LVDVYNFNNITNLIK.S	1779.96399	0.00212	2	2.64E-09	5.44	0.50	2403.5	23/28
	5150	K.ELEDMTINLINELMK.R	1805.90234	0.00030	2	2.12E-07	3.60	0.59	1269.9	20/28
	1563	K.INDNNNNNINCDNTK.N	1719.73511	0.00127	2	5.00E-06	3.16	0.42	623.3	17/28
	4443	R.SPFLVSLFK.G	1037.60303	-0.00154	2	1.79E-04	2.91	0.49	942.3	14/16
	4411	R.ENVVDIFNSYMK.L	1458.69336	0.00066	2	4.01E-03	2.46	0.55	1250.9	17/22
	3285	K.LFQELYK.R	940.51385	-0.03602	2	4.06E-01	2.08	0.12	405.3	11/12
28	gi|547754|sp|P35908.1|K22E_HUMAN Keratin, type II cytoskeletal 2 epidermal (Cytokeratin-2e) (CK 2e) (K2e) (keratin-2)					5.90E-11	60.23		65825.4	6 (6 0 0 0 0)
	1389	R.GSSSGGGYSSGSSSYGSGGR.Q	1740.70557	0.00407	2	5.90E-11	4.69	0.68	1991.3	27/38
	1546	R.GGSGGGGSISGGGYGSGGGSGGR.Y	1741.74841	-0.00386	2	1.26E-09	4.45	0.73	902.6	23/44
	1944	R.SKEEAEALYHSK.Y	1391.68018	0.00037	3	4.20E-05	3.32	0.42	1243.5	24/44
	2517	R.GFSSGSAVVSGGSR.R	1254.60730	-0.00252	2	1.56E-05	3.09	0.52	2002.0	22/26
	1551	R.YGSGGGSKGGSISGGGYGSGGGK.H	1890.85767	-0.00571	3	9.82E-03	2.94	0.54	399.0	22/88
	3024	R.YLDGLTAER.T	1037.52625	0.00078	2	1.85E-01	2.28	0.41	711.3	14/16
29	psu|PFE0060w | organism=Plasmodium_falciparum_3D7 | product=hypothetical protein | location=MAL5:64065-65489(+) | l					4.83E-10	54.25		48690.3	6 (5 0 0 1 0)
	1459	K.NHEESNANMNNHNSFNDK.S	2115.85327	-0.00405	3	4.83E-10	5.09	0.64	1542.3	37/68
	4371	R.TLILGSFPQAGEILR.E	1614.92139	0.00114	2	1.05E-03	4.63	0.50	1224.5	23/28
	3753	K.FSYALDPNDYASIEDK.L	1847.83337	0.00139	2	9.86E-10	4.33	0.62	1180.9	22/30
	2949	K.HLLQQINTYK.H	1257.69495	-0.00362	2	1.79E-05	3.69	0.53	957.9	16/18
	1640	K.HEENHKPQMVGQAPPEK.E	1955.93921	-0.00608	4	1.53E-02	3.10	0.42	445.6	23/96
	2415	K.NEQDTNNTHTPNHDEYSHNLPK.N	2605.12988	-0.06292	5	1.20E-01	1.36	0.22	338.1	27/168
30	gi|42716291|ref|NP_976217.1| erythrocyte membrane protein band 4.1 (elliptocytosis 1, RH-linked) isoform 2 [Homo sapien					2.57E-08	50.23		71911.1	5 (5 0 0 0 0)
	3060	K.KLSMYGVDLHK.A	1290.68750	-0.00052	3	6.24E-03	4.69	0.39	1637.9	27/40
	3233	K.LSMYGVDLHK.A	1162.59253	0.00224	2	5.45E-05	2.87	0.48	1210.3	16/18
	4017	R.SMTPAQADLEFLENAK.K	1764.84729	-0.00032	2	2.57E-08	2.30	0.50	423.1	16/30
	2439	R.LTSTDTIPK.S	975.53571	-0.01119	2	9.73E-02	2.27	0.26	439.7	12/16
	2892	K.TWLDSAK.E	820.41992	0.00042	2	1.72E-01	2.11	0.25	270.0	9/12
31	psu|PF14_0186 | organism=Plasmodium_falciparum_3D7 | product=hypothetical protein | location=MAL14:797708-799465(					9.40E-05	50.21		69935.1	5 (5 0 0 0 0)
	2611	K.IINDNSNLSAPEK.V	1414.71729	0.00175	2	1.15E-02	4.17	0.45	1366.2	19/24
	2373	K.NENNMLDTTNQK.D	1421.63257	-0.00191	2	2.30E-03	3.73	0.47	830.5	17/22
	4056	K.INFYFLPNQEK.K	1412.72083	0.00187	2	6.82E-03	3.18	0.46	692.3	13/20
	4781	K.YNIMNIPTMVLLDR.N	1692.88110	-0.00239	2	9.40E-05	2.77	0.50	662.6	16/26
	2829	K.HINNFTYIK.H	1149.60510	-0.00398	2	4.83E-02	2.41	0.26	520.8	12/16
32	psu|PF08_0054 | organism=Plasmodium_falciparum_3D7 | product=heat shock 70 kDa protein | location=MAL8:861481-86					5.65E-07	50.20		73868.6	5 (5 0 0 0 0)
	2832	R.NENVDIIANDQGNR.T	1571.74084	-0.00178	2	5.65E-07	4.10	0.51	1435.5	21/26
	3173	K.SGVDEKPMIEVTYQGEK.K	1909.92114	-0.00112	3	1.08E-04	3.62	0.42	533.2	24/64
	2620	K.FTESSVQSDMK.H	1258.56201	-0.00007	2	2.74E-06	3.50	0.47	1545.4	18/20

	4864	K.TITTILEWLEK.N	1346.75659	-0.00019	2	6.22E-05	2.78	0.43	809.8	14/20
	2027	R.LSQDEIDR.M	975.47418	-0.00135	2	2.67E-03	2.57	0.20	992.8	13/14
33	psu|PF10_0130 | organism=Plasmodium_falciparum_3D7 | product=hypothetical protein | location=MAL10:515720-517606(					2.60E-07	50.18		75420.2	5 (5 0 0 0 0)
	4471	K.TLGIDSFLDIEDDTK.I	1681.81665	-0.00044	2	2.60E-07	3.57	0.55	1441.3	21/28
	4535 - 4537	K.NNLINLLYIYK.D	1380.78857	0.00005	2	3.79E-04	3.45	0.40	1112.0	16/20
	3803	K.FAYFDLTR.N	1032.51489	-0.00117	2	1.38E-03	2.56	0.40	633.4	13/14
	3489	K.LQQSIYFIK.N	1139.64587	-0.00141	2	2.28E-02	2.08	0.32	425.1	12/16
	3160	K.QQDNQTFLLTK.Q	1335.69031	-0.00118	2	1.02E-03	2.01	0.54	131.5	10/20
34	gi|57864582|ref|NP_001009931.1| hornerin [Homo sapiens]					5.55E-08	40.34		282225.7	4 (4 0 0 0 0)
	1398	R.SSSRGPYESGSGHSSGLGHQESR.S	2346.04541	0.00185	4	5.55E-08	6.75	0.63	4415.4	55/132
	1359	R.HGSGSGHSSSYGQHGSGSGWSSSSGR.H	2477.02100	0.00478	4	1.65E-07	3.89	0.59	905.6	40/150
	1440	R.GPYESGSGHSSGLGHQESR.S	1928.84814	-0.00106	3	7.17E-03	2.81	0.45	592.3	25/72
	1310	R.SEQHGSSSGLSSSYGQHGSGSHQSSGHGR.Q	2869.22290	-0.00005	5	2.09E-01	2.52	0.24	765.8	44/224
35	psu|PFD0090c | organism=Plasmodium_falciparum_3D7 | product=hypothetical protein, conserved in P.falciparum | location					6.66E-08	40.31		49712.6	4 (4 0 0 0 0)
	3612	R.FGSYEQVLISQPHEFNER.L	2180.04077	0.00321	3	6.66E-08	6.21	0.57	2338.4	33/68
	1451	K.KLSQDYNDVNKK.F	1451.74890	-0.00308	3	9.41E-04	3.73	0.37	993.2	24/44
	1653	K.KLSQDYNDVNK.K	1323.65393	-0.00349	2	2.44E-05	3.32	0.48	1437.4	16/20
	1498	K.LSQDYNDVNKK.F	1323.65393	-0.00459	2	5.11E-04	2.69	0.28	814.4	14/20
36	psu|PF14_0593 | organism=Plasmodium_falciparum_3D7 | product=hypothetical protein | location=MAL14:2524856-252892					1.76E-08	40.27		161116.5	4 (4 0 0 0 0)
	1688	K.KNDTNNQTNDLNSNKDETSGSQVTNR.F	2894.31079	-0.00474	4	1.62E-04	5.38	0.44	1524.7	44/150
	4466	K.ILDTLNNNLENNSLILESIASK.N	2428.29297	0.00192	3	1.76E-08	4.96	0.63	2271.8	36/84
	4697	K.SYEYEKDEYNEEIIDLLNLYK.T	2683.26611	0.00022	3	4.10E-04	3.42	0.28	463.0	20/80
	3848	K.LPPLLNEIK.K	1036.64014	-0.00020	2	2.58E-03	2.15	0.27	361.2	13/16
37	gi|4504301|ref|NP_003529.1| histone cluster 1, H4a [Homo sapiens]					7.38E-05	40.21		11360.4	4 (4 0 0 0 0)
	2868	R.DNIQGITKPAIR.R	1325.75354	-0.00276	2	1.72E-04	3.85	0.20	928.1	17/22
	3143	R.ISGLIYEETR.G	1180.62085	-0.00410	2	1.21E-04	3.52	0.50	1067.1	16/18
	3664	K.VFLENVIR.D	989.57782	-0.00215	2	7.93E-03	3.22	0.34	479.7	12/14
	1666 - 1668	R.DAVTYTEHAK.R	1134.54260	-0.00483	2	7.38E-05	2.08	0.43	494.3	13/18
38	psu|PFI0605c | organism=Plasmodium_falciparum_3D7 | product=hypothetical protein, conserved | location=MAL9:551417-					1.29E-06	40.21		52671.4	4 (4 0 0 0 0)
	3303	K.VNNNLLNSIEK.R	1257.67969	-0.00227	2	1.92E-03	4.14	0.29	1410.1	18/20
	2749	K.IDGIYDDHDYNK.N	1467.63867	0.00005	2	1.29E-06	3.25	0.49	1208.1	18/22
	4151 - 4153	K.TSVNDSDGVEILVANQVFNNK.N	2263.12012	0.00403	3	8.19E-06	2.91	0.54	566.9	23/80
	2764	K.LYIDPDNEK.N	1106.53638	-0.00215	2	1.07E-03	2.01	0.14	1040.7	14/16
39	gi|4503595|ref|NP_000493.1| eosinophil peroxidase [Homo sapiens]					5.76E-06	40.20		80989.3	4 (4 0 0 0 0)
	2767	R.RPLLGASNQALAR.W	1366.79138	-0.00082	3	1.24E-04	4.00	0.43	1779.5	27/48
	3699	R.IVYEGGIDPILR.G	1344.75220	-0.00020	2	5.76E-06	3.87	0.57	1859.8	19/22
	4174 - 4176	R.ASAPNSHVPLSSAFFASWR.I	2032.00354	-0.00070	3	2.84E-03	3.63	0.52	663.5	25/72
	4100	R.TNYLGLLAINQR.F	1375.76917	-0.00095	3	1.90E-01	3.46	0.18	959.5	21/44
40	psu|PF14_0036 | organism=Plasmodium_falciparum_3D7 | product=acid phosphatase, putative | location=MAL14:141410-1					3.58E-04	40.18		35800.9	4 (4 0 0 0 0)
	4069	K.FPLDPIQTNQITDLK.N	1742.93237	0.00103	2	3.58E-04	3.61	0.42	1668.6	24/28
	4302	K.LKPPFVVALGDLTNK.F	1611.94690	-0.00100	3	3.78E-02	2.56	0.25	700.4	25/56
	3232	K.QITISAVGMQAK.D	1246.68237	-0.00300	2	7.78E-04	2.54	0.47	383.3	12/22

<table>
<tr><td></td><td>3857</td><td>K.SGIFIVQVTEDK.V</td><td>1335.71545</td><td>-0.00105</td><td>2</td><td>1.34E-03</td><td>2.32</td><td>0.51</td><td>746.0</td><td>14/22</td></tr>
<tr><td>41</td><td colspan="5">psu|PFA0195w | organism=Plasmodium_falciparum_3D7 | product=hypothetical protein, conserved | location=MAL1:173099</td><td>3.14E-07</td><td>40.16</td><td></td><td>67759.6</td><td>4 (4 0 0 0 0)</td></tr>
<tr><td></td><td>4247</td><td>K.NTINQTIDVLNSITK.L</td><td>1673.90686</td><td>0.00054</td><td>2</td><td>3.14E-07</td><td>3.15</td><td>0.64</td><td>692.2</td><td>16/28</td></tr>
<tr><td></td><td>1901</td><td>K.INYDQNYNK.D</td><td>1171.53784</td><td>-0.00081</td><td>2</td><td>3.90E-04</td><td>2.75</td><td>0.36</td><td>720.8</td><td>13/16</td></tr>
<tr><td></td><td>1917</td><td>K.FMNHQINSEK.K</td><td>1247.58374</td><td>-0.00447</td><td>2</td><td>3.76E-03</td><td>2.21</td><td>0.43</td><td>520.3</td><td>14/18</td></tr>
<tr><td></td><td>1580</td><td>K.NLTNQTEEPK.N</td><td>1173.57458</td><td>-0.00373</td><td>2</td><td>2.78E-02</td><td>2.14</td><td>0.27</td><td>509.7</td><td>12/18</td></tr>
<tr><td>42</td><td colspan="5">psu|MAL7P1.5 | organism=Plasmodium_falciparum_3D7 | product=Plasmodium falciparum Maurer's Cleft 2 transmembrane</td><td>8.05E-11</td><td>30.33</td><td></td><td>27539.4</td><td>3 (3 0 0 0 0)</td></tr>
<tr><td></td><td>4573</td><td>K.KKNPDDQISDLVSLVDNMNITQEK.K</td><td>2744.37720</td><td>0.00186</td><td>3</td><td>8.05E-11</td><td>6.59</td><td>0.30</td><td>2372.3</td><td>37/92</td></tr>
<tr><td></td><td>4693</td><td>K.KNPDDQISDLVSLVDNMNITQEK.K</td><td>2616.28223</td><td>0.00247</td><td>3</td><td>3.95E-04</td><td>4.52</td><td>0.10</td><td>786.7</td><td>26/88</td></tr>
<tr><td></td><td>4799</td><td>K.NPDDQISDLVSLVDNMNITQEK.K</td><td>2488.18726</td><td>0.00180</td><td>3</td><td>1.88E-05</td><td>4.29</td><td>0.43</td><td>1177.8</td><td>32/84</td></tr>
<tr><td>43</td><td colspan="5">psu|PFB0100c | organism=Plasmodium_falciparum_3D7 | product=knob associated histidine-rich protein | location=MAL2:1</td><td>2.23E-11</td><td>30.29</td><td></td><td>71259.4</td><td>3 (3 0 0 0 0)</td></tr>
<tr><td></td><td>1722 - 1724</td><td>K.GASTTAGSTTGATTGANAVQSK.D</td><td>1938.93628</td><td>0.00585</td><td>2</td><td>3.17E-08</td><td>5.75</td><td>0.66</td><td>1217.3</td><td>22/42</td></tr>
<tr><td></td><td>3681</td><td>R.FPLGMNDEDEEGKEALAIK.D</td><td>2106.00586</td><td>0.00034</td><td>3</td><td>2.23E-11</td><td>5.01</td><td>0.52</td><td>1511.4</td><td>36/72</td></tr>
<tr><td></td><td>1584</td><td>K.NAANNGEQVMSR.G</td><td>1290.58557</td><td>-0.00557</td><td>2</td><td>8.40E-06</td><td>3.05</td><td>0.56</td><td>1109.7</td><td>16/22</td></tr>
<tr><td>44</td><td colspan="5">psu|PF10_0104 | organism=Plasmodium_falciparum_3D7 | product=hypothetical protein | location=MAL10:422960-424364(</td><td>2.58E-08</td><td>30.28</td><td></td><td>25661.7</td><td>3 (3 0 0 0 0)</td></tr>
<tr><td></td><td>3393 - 3395</td><td>K.WGTGSGNQLVTAVTTNK.N</td><td>1733.88171</td><td>-0.00099</td><td>2</td><td>2.58E-08</td><td>5.69</td><td>0.61</td><td>1206.4</td><td>20/32</td></tr>
<tr><td></td><td>3956</td><td>K.NTSYWEIGENVYLK.N</td><td>1715.82751</td><td>0.00152</td><td>2</td><td>1.28E-07</td><td>3.02</td><td>0.60</td><td>732.1</td><td>18/26</td></tr>
<tr><td></td><td>2579</td><td>K.NINQNGYLSTSK.S</td><td>1338.66479</td><td>-0.00080</td><td>2</td><td>1.47E-03</td><td>2.95</td><td>0.41</td><td>832.6</td><td>16/22</td></tr>
<tr><td>45</td><td colspan="2">gi|1360744|pir||B35049 ankyrin 1, erythrocyte splice form 3 - human [MASS=203445</td><td></td><td></td><td></td><td>1.97E-09</td><td>30.28</td><td></td><td>203319.9</td><td>3 (3 0 0 0 0)</td></tr>
<tr><td></td><td>3532</td><td>R.TAAVLLQNDPNPDVLSK.T</td><td>1794.95959</td><td>0.00347</td><td>2</td><td>6.70E-05</td><td>5.66</td><td>0.50</td><td>1536.8</td><td>21/32</td></tr>
<tr><td></td><td>3436</td><td>R.ISEILLDHGAPIQAK.T</td><td>1604.90063</td><td>-0.00019</td><td>2</td><td>1.97E-09</td><td>4.13</td><td>0.54</td><td>1965.8</td><td>23/28</td></tr>
<tr><td></td><td>5436</td><td>R.MSFPETVDEILDVSEDEGEELISFK.A</td><td>2858.31763</td><td>0.00833</td><td>3</td><td>8.86E-08</td><td>3.23</td><td>0.50</td><td>404.2</td><td>24/96</td></tr>
<tr><td>46</td><td colspan="5">psu|PFB0840w | organism=Plasmodium_falciparum_3D7 | product=replication factor C, subunit 2 | location=MAL2:738859-7</td><td>4.29E-11</td><td>30.26</td><td></td><td>37898.7</td><td>3 (3 0 0 0 0)</td></tr>
<tr><td></td><td>5106</td><td>K.TVQDIIEDGFDVAYIFK.S</td><td>1972.99023</td><td>-0.00398</td><td>2</td><td>4.29E-11</td><td>5.21</td><td>0.67</td><td>3214.3</td><td>26/32</td></tr>
<tr><td></td><td>4522</td><td>K.TSAINALAHELFGK.E</td><td>1471.79041</td><td>0.00151</td><td>2</td><td>6.25E-05</td><td>3.64</td><td>0.55</td><td>1104.7</td><td>17/26</td></tr>
<tr><td></td><td>2692</td><td>K.IIETTEGDLR.R</td><td>1146.60010</td><td>-0.00337</td><td>2</td><td>6.21E-02</td><td>3.27</td><td>0.46</td><td>622.0</td><td>13/18</td></tr>
<tr><td>47</td><td colspan="5">psu|PFC0110w | organism=Plasmodium_falciparum_3D7 | product=Cytoadherence linked asexual protein 3.1 | location=MA</td><td>2.14E-07</td><td>30.26</td><td></td><td>167381.1</td><td>3 (3 0 0 0 0)</td></tr>
<tr><td></td><td>2425</td><td>K.NENANVNTPENLNK.L</td><td>1570.74561</td><td>-0.00020</td><td>2</td><td>2.65E-05</td><td>5.24</td><td>0.47</td><td>1605.7</td><td>21/26</td></tr>
<tr><td></td><td>3480</td><td>K.LLNEYDNIEQLK.S</td><td>1491.76892</td><td>0.00090</td><td>2</td><td>4.63E-05</td><td>3.37</td><td>0.50</td><td>1412.1</td><td>17/22</td></tr>
<tr><td></td><td>4362</td><td>K.NITDADDETYIIPTVQSSFHDIVK.Y</td><td>2721.32544</td><td>0.00321</td><td>3</td><td>2.14E-07</td><td>3.31</td><td>0.44</td><td>444.4</td><td>27/92</td></tr>
<tr><td>48</td><td colspan="5">psu|PF13_0143 | organism=Plasmodium_falciparum_3D7 | product=phosphoribosylpyrophosphate synthetase | location=M/</td><td>1.01E-10</td><td>30.25</td><td></td><td>49351.6</td><td>3 (3 0 0 0 0)</td></tr>
<tr><td></td><td>4671</td><td>R.VPVDNLEAQLIGLDYFTK.K</td><td>2035.07458</td><td>0.00310</td><td>2</td><td>1.01E-10</td><td>5.04</td><td>0.68</td><td>1854.5</td><td>25/34</td></tr>
<tr><td></td><td>2765</td><td>R.VPISAADVAR.M</td><td>998.56293</td><td>-0.00234</td><td>2</td><td>1.99E-04</td><td>2.32</td><td>0.36</td><td>875.1</td><td>17/18</td></tr>
<tr><td></td><td>4210</td><td>K.ESLNDLFNIK.S</td><td>1192.62085</td><td>-0.00032</td><td>2</td><td>2.50E-03</td><td>2.30</td><td>0.26</td><td>628.3</td><td>13/18</td></tr>
<tr><td>49</td><td colspan="5">psu|PF14_0538 | organism=Plasmodium_falciparum_3D7 | product=hypothetical protein | location=MAL14:2314316-232149</td><td>4.79E-10</td><td>30.25</td><td></td><td>283024.1</td><td>3 (3 0 0 0 0)</td></tr>
<tr><td></td><td>3131</td><td>K.HNYFDVEHNDTNSEIVK.Y</td><td>2060.93091</td><td>-0.00107</td><td>3</td><td>1.35E-05</td><td>4.96</td><td>0.53</td><td>2500.6</td><td>33/64</td></tr>
<tr><td></td><td>3199</td><td>K.NNSNNSTLDIINDDR.S</td><td>1704.77832</td><td>-0.00117</td><td>2</td><td>2.40E-08</td><td>4.42</td><td>0.54</td><td>1783.2</td><td>21/28</td></tr>
<tr><td></td><td>4608</td><td>R.NLIISQLIDNINEDR.K</td><td>1769.93921</td><td>0.00456</td><td>2</td><td>4.79E-10</td><td>4.03</td><td>0.59</td><td>1966.7</td><td>23/28</td></tr>
<tr><td>50</td><td colspan="5">psu|MAL8P1.98 | organism=Plasmodium_falciparum_3D7 | product=serine protease, putative | location=MAL8:616354-618</td><td>8.43E-07</td><td>30.24</td><td></td><td>42818.5</td><td>3 (3 0 0 0 0)</td></tr>
<tr><td></td><td>4590</td><td>K.VDDLIFLGSGFIYNK.N</td><td>1700.88940</td><td>-0.00019</td><td>2</td><td>5.33E-06</td><td>4.47</td><td>0.57</td><td>2015.3</td><td>22/28</td></tr>
<tr><td></td><td>3189</td><td>K.QGEVVIAYGQIQK.F</td><td>1432.77942</td><td>0.00017</td><td>2</td><td>8.43E-07</td><td>4.08</td><td>0.48</td><td>1939.6</td><td>20/24</td></tr>
<tr><td></td><td>3983</td><td>K.IDNYGLALPSNVLK.N</td><td>1516.83691</td><td>0.00249</td><td>2</td><td>1.04E-03</td><td>3.13</td><td>0.56</td><td>663.5</td><td>16/26</td></tr>
</table>

51	psu|PF14_0445 | organism=Plasmodium_falciparum_3D7 | product=hypothetical protein | location=MAL14:1920913-192158					9.66E-06	30.23		20330.7	3 (3 0 0 0 0)
	4775 - 4777	K.FNDIFLFIEDTTK.N	1602.80505	0.00200	2	9.66E-06	4.57	0.57	1523.1	19/24
	2235	K.NLNPYNNNDEEK.Q	1463.63977	-0.00349	2	6.06E-05	3.51	0.40	590.2	17/22
	2015	K.QNSDVLNQER.N	1202.57605	-0.00069	2	1.00E-03	2.45	0.41	430.1	11/18
52	psu|PFE0810c | organism=Plasmodium_falciparum_3D7 | product=40S ribosomal subunit protein S14, putative | location=M					1.63E-07	30.22		16066.7	3 (3 0 0 0 0)
	2867	K.TPQPETAIVSGPQPK.E	1549.82202	0.00017	2	1.86E-06	4.40	0.60	1004.1	21/28
	3031	R.IEDVTPIPTDSTR.K	1443.73254	-0.00031	2	1.63E-07	4.15	0.51	1052.2	20/24
	1736	K.SKTPGPGAQSALR.A	1269.69092	-0.00448	3	4.10E-02	2.63	0.40	547.2	20/48
53	psu|PFL1005c | organism=Plasmodium_falciparum_3D7 | product=chromodomain protein | location=MAL12:831245-832045					7.91E-09	30.22		30999.3	3 (3 0 0 0 0)
	4131	K.NLHNVGDELYISVIHNINNK.E	2306.18872	0.00248	3	7.91E-09	4.35	0.52	925.3	33/76
	3183	K.NESPQWVEETNIR.R	1601.75537	0.00176	2	5.31E-06	4.35	0.57	1645.7	20/24
	3652	K.HENHVNDGNLLNVEDVYSVR.I	2323.10620	-0.00352	4	5.52E-03	2.97	0.35	876.6	33/114
54	psu|PF14_0301 | organism=Plasmodium_falciparum_3D7 | product=hypothetical protein | location=MAL14:1260850-126276					3.34E-06	30.21		33245.8	3 (3 0 0 0 0)
	4081	K.FDNSGISLLDIK.Y	1321.69983	-0.00056	2	6.30E-04	4.19	0.42	1898.6	20/22
	4474	K.SINDNAISVEVIQNIMEK.K	2017.02698	0.00359	2	3.34E-06	3.79	0.61	811.5	19/34
	3617	K.LLNKPSLDLLK.N	1253.78271	0.00074	3	1.73E-03	2.50	0.16	362.5	21/40
55	psu|PFC1080c | organism=Plasmodium_falciparum_3D7 | product=Plasmodium falciparum Maurer's Cleft 2 transmembrane					3.44E-05	30.19		26987.2	3 (3 0 0 0 0)
	4650	K.KNPDDQISSLVSLVDNMNITQEK.K	2588.28711	0.00461	3	2.10E-03	3.88	0.21	802.1	28/88
	4514	K.KKNPDDQISSLVSLVDNMNITQEK.K	2716.38208	0.00039	4	4.53E-05	3.51	0.39	1026.7	33/138
	3764	K.EYMDSYLMHLR.M	1457.65515	0.00200	2	3.44E-05	2.64	0.35	623.8	16/20
56	psu|PF13_0032 | organism=Plasmodium_falciparum_3D7 | product=hydrolase, putative | location=MAL13:289766-291202(-					6.44E-08	30.19		56775.0	3 (3 0 0 0 0)
	3932	R.YIQEALYDPNIQIMK.N	1838.93567	0.00017	2	6.44E-08	3.07	0.47	1133.7	18/28
	3161	K.NTENIIYIDK.Y	1222.63135	-0.00203	2	2.99E-05	2.96	0.49	919.3	16/18
	3517	K.YEEIGIPISNSTVK.G	1549.81079	0.00090	2	6.10E-03	2.41	0.48	285.8	13/26
57	psu|PF10_0323 | organism=Plasmodium_falciparum_3D7 | product=early transcribed membrane protein 10.2, etramp 10.2					2.14E-06	30.19		38902.0	3 (3 0 0 0 0)
	2323	K.AIEQDLQQK.K	1072.56335	0.00066	2	1.93E-02	3.29	0.32	940.7	13/16
	4287	K.FNDNDLLLAAESLK.E	1562.80603	0.00090	2	2.14E-06	3.29	0.55	1702.4	20/26
	2524	K.LDDEKKEDLLK.F	1345.72095	0.00040	3	9.14E-05	3.15	0.19	1569.2	28/40
58	psu|PF10_0366 | organism=Plasmodium_falciparum_3D7 | product=ADP/ATP transporter on adenylate translocase | locatio					1.31E-04	30.19		33704.7	3 (3 0 0 0 0)
	3624	K.MLIQTQDSIPEIK.S	1515.80872	0.00017	2	1.31E-04	3.71	0.39	1298.6	20/24
	1841	R.YDQNTDFSK.F	1117.47961	-0.00031	2	1.52E-02	2.64	0.43	737.4	14/16
	3912	K.EQGVLSLWR.G	1087.58948	-0.00118	2	6.42E-03	2.38	0.26	509.9	12/16
59	psu|PF08_0076 | organism=Plasmodium_falciparum_3D7 | product=40S ribosomal protein S16, putative | location=MAL8:67					6.61E-06	30.17		16275.1	3 (3 0 0 0 0)
	4262	K.VYEPLWLIGSGK.L	1361.74634	0.00066	2	6.61E-06	3.39	0.47	809.1	17/22
	4155	K.NLDLVEPYILK.T	1316.74597	0.00151	2	7.54E-03	2.94	0.28	782.5	14/20
	2779	K.GGGQTSQIYAIR.Q	1250.64880	-0.00337	2	3.69E-02	2.75	0.26	1101.2	14/22
60	psu|PF07_0129 | organism=Plasmodium_falciparum_3D7 | product=acyl-coA synthetase, PfACS5 | location=MAL7:142353					4.32E-06	30.17		93224.9	3 (3 0 0 0 0)
	4107	K.SGSIFSGYFLEK.E	1334.66272	0.00029	2	2.18E-05	3.34	0.52	1085.0	18/22
	2025	K.TGITEDNYNDR.L	1297.56555	-0.00203	2	4.32E-06	2.77	0.55	901.5	16/20
	3817	K.IWDTNNYLTPTLK.V	1578.81628	0.00200	2	2.91E-05	2.59	0.42	438.3	15/24
61	psu|PF10_0187 | organism=Plasmodium_falciparum_3D7 | product=ribosomal protein L30e, putative | location=MAL10:786					2.08E-04	30.17		11767.1	3 (3 0 0 0 0)
	3321	K.LVIVSSNCPSIQR.S	1415.76746	-0.00179	2	1.09E-02	3.31	0.48	1094.5	17/24

	3840	R.SVIEYYAMLSK.C	1303.66028	-0.00069	2	2.52E-02	2.88	0.48	1170.2	15/20
	5486	R.ISCLVITDVGDSDIIK.T	1690.89319	0.00505	2	2.08E-04	2.69	0.45	691.8	15/30
62	psu|PFL1925w | organism=Plasmodium_falciparum_3D7 | product=cell division protein FtsH, putative | location=MAL12:165				5.41E-04	30.16		100894.9	3 (3 0 0 0 0)
	4506	K.ELDNLAFALLDK.E	1361.73108	0.00005	2	5.41E-04	3.26	0.47	1089.7	17/22
	5085	R.FEEIAGIDESKLELLEVVDFIK.N	2536.34326	0.00290	3	8.45E-03	2.83	0.49	788.6	27/84
	3400	R.YYENFFNR.R	1152.51086	-0.00092	2	1.31E-02	2.24	0.45	530.2	11/14
63	psu|PFD1015w | organism=Plasmodium_falciparum_3D7 | product=hypothetical protein, conserved | location=MAL4:100605				2.84E-05	30.16		44902.0	3 (3 0 0 0 0)
	3021 - 3023	R.GDINMDQFNEK.K	1310.56812	-0.00038	2	3.18E-05	2.85	0.45	890.4	14/20
	3712	K.LTTVALGLGENPSLR.S	1540.86938	-0.00056	2	2.84E-05	2.68	0.51	641.1	16/28
	3181	K.DFTNYIQR.I	1056.51086	-0.00105	2	6.06E-03	2.67	0.32	366.4	11/14
64	psu|PFE0080c | organism=Plasmodium_falciparum_3D7 | product=rhoptry-associated protein 2, RAP2 | location=MAL5:840				3.62E-07	30.15		46708.8	3 (3 0 0 0 0)
	4000	K.SMLSTDDYQSFFK.N	1568.69373	0.00163	2	3.62E-07	2.96	0.61	903.7	18/24
	3300	K.SNPYFIVGSR.V	1139.58435	-0.00203	2	5.89E-05	2.77	0.55	585.5	14/18
	3887	K.YTEISVLNYVR.D	1356.71582	-0.00203	2	1.11E-04	2.37	0.37	523.7	17/20
65	psu|PF14_0159 | organism=Plasmodium_falciparum_3D7 | product=hypothetical protein, conserved | location=MAL14:6454				1.98E-05	30.15		110641.7	3 (3 0 0 0 0)
	4695	R.DWFEEFAPIEVVR.N	1636.80054	0.00200	2	1.98E-05	2.95	0.59	880.8	17/24
	2179	K.STINEDINNK.L	1147.55896	-0.00203	2	5.86E-03	2.24	0.35	694.8	13/18
	2097	R.DAQYIQETGSK.M	1239.58521	-0.00142	2	6.07E-04	2.01	0.39	535.6	12/20
66	psu|PFE0675c | organism=Plasmodium_falciparum_3D7 | product=deoxyribodipyrimidine photolyase (photoreactivating enz				1.64E-04	30.13		129116.7	3 (3 0 0 0 0)
	4087	K.NNVLLLLTR.D	1055.65710	-0.00129	2	2.05E-03	2.66	0.27	879.1	13/16
	3993	R.INDNWSLIYAYEK.A	1628.79553	0.00310	2	1.64E-04	2.39	0.53	255.8	13/24
	3847	K.ELFSEEMIMK.K	1256.59009	0.00151	2	4.66E-03	2.07	0.46	1033.3	14/18
67	gi|155969697|ref|NP_775109.2| keratin 6C [Homo sapiens]				1.58E-11	20.32		59988.4	2 (2 0 0 0 0)
	5143	R.NLDLDSIIAEVKAQYEEIAQR.S	2418.25098	-0.00125	3	1.58E-11	6.10	0.62	1341.6	40/80
	2915	R.SGFSSISVSR.S	1026.52148	-0.00373	2	1.72E-03	3.12	0.37	984.1	14/18
68	psu|PF14_0627 | organism=Plasmodium_falciparum_3D7 | product=ribosomal protein S3, putative | location=MAL14:26840				1.32E-08	20.30		24652.4	2 (2 0 0 0 0)
	4813	K.FINDGVFQAELNEFLAR.I	1982.99707	0.00383	2	1.32E-08	5.38	0.63	1664.6	24/32
	3124	R.ILAEDGYSGVEVR.V	1407.71143	-0.00106	2	1.00E-07	3.76	0.64	1827.3	20/24
69	psu|PF11_0224 | organism=Plasmodium_falciparum_3D7 | product=circumsporozoite-related antigen | location=MAL11:815				7.43E-08	20.29		17285.0	2 (2 0 0 0 0)
	4787 - 4789	K.KGSGEPLIDVHDLISDMIK.K	2067.07910	-0.00030	3	7.43E-08	5.86	0.52	1691.5	34/72
	2321	K.KEEELVEVNKR.K	1372.74304	0.00074	3	6.59E-04	3.63	0.18	1135.8	22/40
70	psu|PF13_0082 | organism=Plasmodium_falciparum_3D7 | product=cop-coated vesicle membrane protein p24 precursor, p				4.43E-08	20.27		24426.7	2 (2 0 0 0 0)
	4984	K.TSELFDQFLEVFDEQER.M	2131.98193	0.00225	2	4.43E-08	5.36	0.48	2300.0	24/32
	3749	K.NNMIVGSYEFMDR.K	1575.69299	0.00164	2	7.69E-07	4.24	0.56	1709.6	21/24
71	psu|PFC0730w | organism=Plasmodium_falciparum_3D7 | product=conserved protein, putative | location=MAL3:671131-67				1.94E-06	20.26		26023.9	2 (2 0 0 0 0)
	3336	K.ISQTATSHLTQITGNLTEK.L	2043.07166	-0.00192	3	1.94E-06	5.30	0.46	940.6	30/72
	3392	R.VFNNIDDYVK.K	1226.60522	-0.00178	2	3.83E-05	3.20	0.51	1114.4	16/18
72	psu|PFD1055w | organism=Plasmodium_falciparum_3D7 | product=ribosomal protein S19s, putative | location=MAL4:10310				3.13E-08	20.26		19709.6	2 (2 0 0 0 0)
	4388 - 4390	R.SILQQLENLGYVEQNPK.K	1973.03381	0.00231	2	1.33E-07	5.24	0.49	1527.4	21/32
	4326	K.LAPLNEDWYFIR.A	1536.78455	0.00139	2	3.13E-08	3.54	0.63	302.8	17/22
73	psu|PFE1465w | organism=Plasmodium_falciparum_3D7 | product=hypothetical protein, conserved | location=MAL5:119915				1.06E-07	20.25		191498.5	2 (2 0 0 0 0)
	5179	K.FDPSNMTILNLIEDIK.W	1862.95679	0.00127	2	1.06E-07	4.99	0.59	1635.2	24/30

	1526	K.VTDENDAIDTQNKNEHND.-	2071.87988	-0.00692	3	2.47E-03	3.09	0.35	272.7	21/68
74	gi|7245526|pdb|1DEJ|A Chain A, Crystal Structure Of A DictyosteliumTETRAHYMENA CHIMERA Actin (Mutant 646: Q228k					2.19E-07	20.24		41719.9	2 (2 0 0 0 0)
	3828	K.SYELPDGQVITIGNER.F	1790.89197	0.00285	2	2.19E-07	4.71	0.45	1601.8	25/30
	2704	R.GYSFTTTAER.E	1132.52698	-0.00264	2	8.35E-05	2.34	0.63	630.9	15/18
75	psu|PFC0250c | organism=Plasmodium_falciparum_3D7 | product=AP endonuclease (DNA-(apurinic or apyrimidinic site) lya					2.06E-08	20.23		72397.8	2 (2 0 0 0 0)
	4762	K.SLADYEIMEQILNDDFK.D	2043.95801	-0.00113	3	2.06E-08	4.63	0.58	1248.2	29/64
	3475	K.ILTAGNLVDSYR.Y	1321.71106	-0.00093	2	3.54E-06	4.35	0.53	1303.6	19/22
76	psu|PF11_0065 | organism=Plasmodium_falciparum_3D7 | product=ribosomal protein S4, putative | location=MAL11:23559					1.02E-06	20.23		32333.7	2 (2 0 0 0 0)
	4169	R.LSNVFVIGDNTKPYISLPR.E	2133.17017	-0.00064	3	1.02E-06	4.62	0.45	1404.2	34/72
	2847	R.VGVISSIDK.N	917.53021	-0.00172	2	3.26E-03	2.44	0.39	583.5	12/16
77	psu|MAL13P1.61 | organism=Plasmodium_falciparum_3D7 | product=hypothetical protein, conserved in P. falciparum | loca					2.29E-06	20.23		28236.9	2 (2 0 0 0 0)
	2985 - 2987	K.VETEQSTPAKPEPTEFVNNDIHQNK.N	2852.36963	0.00124	4	2.29E-06	4.52	0.45	688.0	43/144
	2397	K.VHNDLNALK.S	1023.55817	-0.00197	2	1.59E-04	2.11	0.39	369.4	11/16
78	psu|PF14_0579 | organism=Plasmodium_falciparum_3D7 | product=ribosomal protein L27, putative | location=MAL14:2475					7.44E-09	20.22		13785.9	2 (2 0 0 0 0)
	2516	K.KAVIVNTYEGQTR.E	1478.79614	-0.00032	2	7.44E-09	4.35	0.61	1860.1	21/24
	3323	K.VIIILNGR.R	897.58801	-0.00148	2	4.37E-03	2.46	0.37	540.2	11/14
79	psu|PFE0975c | organism=Plasmodium_falciparum_3D7 | product=40S ribosomal subunit protein S24, putative | location=M					2.12E-05	20.22		15381.8	2 (2 0 0 0 0)
	4396	K.LNNVNTIVLFGFK.T	1478.83655	-0.00105	2	2.12E-05	4.37	0.59	1393.9	20/24
	3736	K.QFALEILHPNK.G	1309.72632	-0.00158	3	2.69E-04	3.23	0.28	775.5	23/40
80	psu|PF10_0075 | organism=Plasmodium_falciparum_3D7 | product=asparagine-rich antigen | location=MAL10:301126-3059					6.60E-08	20.22		182550.7	2 (2 0 0 0 0)
	1609	R.LEAEQAGATTTENR.T	1490.70813	-0.00227	2	6.60E-08	3.82	0.57	1713.9	22/26
	2609	R.MQNYAGAGISGQSVHQTNK.L	1990.93994	0.00297	3	4.30E-04	3.24	0.41	572.9	26/72
81	psu|PFA0065w | organism=Plasmodium_falciparum_3D7 | product=Plasmdoium falciparum Maurer's Cleft 2 transmembrane					7.31E-10	20.22		27542.6	2 (2 0 0 0 0)
	4705	K.KNPDAEISDLVNLVDNMNITQEK.K	2600.28711	0.00132	3	5.17E-05	3.79	0.49	592.0	24/88
	4836	K.NPDAEISDLVNLVDNMNITQEK.K	2472.19214	0.00577	3	7.31E-10	3.60	0.56	947.8	29/84
82	gi|157830361|pdb|1BKE|A Chain A, Human Serum Albumin In A Complex With Myristic Acid And Tri- Iodobenzoic Acid					2.27E-07	20.21		65992.9	2 (2 0 0 0 0)
	3276	K.KVPQVSTPTLVEVSR.N	1639.93774	-0.00124	3	2.84E-03	4.28	0.45	1427.0	28/56
	3437	K.VPQVSTPTLVEVSR.N	1511.84277	-0.00056	2	2.27E-07	2.87	0.66	1222.0	20/26
83	psu|PF14_0455 | organism=Plasmodium_falciparum_3D7 | product=multidrug resistance protein 2 (heavy metal transport fa					1.63E-06	20.20		118936.9	2 (2 0 0 0 0)
	1441	K.IYDNNNNNNNNINSK.I	1878.83252	0.00969	2	1.75E-06	3.94	0.48	725.7	20/30
	5033	K.SFTDISDLIDILR.D	1507.80029	-0.00337	2	1.63E-06	3.47	0.50	1342.6	17/24
84	psu|PFI0935w | organism=Plasmodium_falciparum_3D7 | product=DNAJ-like molecular chaperone protein, putative | locatio					2.43E-04	20.20		43232.6	2 (2 0 0 0 0)
	2805	K.IYHPDKNPDESANSSFIK.L	2061.98755	-0.00645	4	2.43E-04	3.90	0.44	1292.3	35/102
	3247	K.QAYDVLTDDVR.R	1294.62732	-0.00178	2	7.04E-04	2.36	0.48	311.4	12/20
85	psu|PFE0050w | organism=Plasmodium_falciparum_3D7 | product=hypothetical protein | location=MAL5:55841-56872(+) | l					1.62E-04	20.19		30696.0	2 (2 0 0 0 0)
	4148	R.INNAFNLDILR.T	1302.71643	0.00078	2	1.62E-04	3.78	0.38	1364.9	18/20
	3484	K.DIYKPIQSYANNFSK.V	1787.89624	-0.00283	3	1.42E-02	2.58	0.24	586.0	21/56
86	psu|PFL2005w | organism=Plasmodium_falciparum_3D7 | product=replication factor c subunit 4 | location=MAL12:1778142					3.11E-07	20.19		37613.7	2 (2 0 0 0 0)
	4620	R.FNIGSEVIQIEFLK.I	1636.89453	0.00078	2	3.11E-07	3.78	0.45	3198.8	23/26
	2781	K.IIDALQSR.C	915.52582	-0.00148	2	3.47E-03	2.42	0.14	700.1	14/14
87	psu|PF14_0083 | organism=Plasmodium_falciparum_3D7 | product=ribosomal protein S8e, putative | location=MAL14:3233					8.65E-06	20.19		25035.4	2 (2 0 0 0 0)
	4320	K.LDSGSFSWPTFGISK.N	1628.79553	-0.00019	2	8.65E-06	3.08	0.59	599.0	16/28

	4186	K.NIDPLLLEQFK.Q	1329.74133	-0.00044	2	1.06E-02	2.67	0.40	492.8	13/20
88	psu|PF14_0563 | organism=Plasmodium_falciparum_3D7 | product=DEAD-box RNA helicase, putative | location=MAL14:24					1.30E-05	20.18		84261.7	2 (2 0 0 0 0)
	5032	K.IDNELIQILTYLK.F	1575.89929	0.00090	2	1.30E-05	3.36	0.49	1353.9	17/24
	1840	R.IGDTLNNTSNQR.V	1332.65027	-0.00142	2	5.89E-05	3.17	0.49	932.4	16/22
89	psu|MAL8P1.146 | organism=Plasmodium_falciparum_3D7 | product=filament assembling protein, putative | location=MAL8					5.61E-04	20.18		86688.6	2 (2 0 0 0 0)
	4818	K.NENFQNFVLEEIATIK.N	1908.97021	-0.00014	3	5.61E-04	3.53	0.47	927.9	25/60
	2653	K.INNISSNIENEK.V	1374.68591	-0.00080	2	1.98E-03	2.96	0.39	920.6	19/22
90	psu|PFE0850c | organism=Plasmodium_falciparum_3D7 | product=60S ribosomal protein L12, putative | location=MAL5:715					2.75E-04	20.18		18100.8	2 (2 0 0 0 0)
	3013	R.QVGGEVGASSVLSPK.L	1414.75366	-0.00044	2	4.07E-03	3.51	0.54	1049.4	21/28
	2952	K.LEQVYSIAR.V	1078.58911	-0.00215	2	2.75E-04	2.73	0.34	1424.7	15/16
91	psu|PF11_0374 | organism=Plasmodium_falciparum_3D7 | product=hypothetical protein | location=MAL11:1422741-142619					2.05E-05	20.17		128465.1	2 (2 0 0 0 0)
	1871	R.EVLVSTNNNNNNNNNNNSGEK.N	2303.02417	0.00901	3	1.78E-02	3.03	0.51	693.0	27/80
	3196	K.NNILNGDADFEK.N	1349.63318	-0.00166	2	2.05E-05	2.72	0.34	677.6	16/22
92	psu|PFD0080c | organism=Plasmodium_falciparum_3D7 | product=hypothetical protein, conserved in P.falciparum | location					2.00E-05	20.17		60233.5	2 (2 0 0 0 0)
	2316	R.TPEESEQAWK.Y	1204.54810	0.00627	2	2.00E-05	3.50	0.40	1426.3	16/18
	3204	K.IVFQDLSK.K	949.53534	-0.00258	2	2.90E-03	2.05	0.19	269.1	11/14
93	psu|MAL13P1.63 | organism=Plasmodium_falciparum_3D7 | product=Plasmodium falciparum asparagine-rich protein | locat					3.49E-06	20.16		139030.0	2 (2 0 0 0 0)
	4527	K.FVIQDVFDLR.S	1251.67322	-0.00069	2	3.05E-04	2.78	0.53	1109.6	15/18
	4355	R.LNTDEIQTVVNQVIDK.A	1828.96509	0.00444	2	3.49E-06	2.76	0.47	680.0	16/30
94	gi|3387905|gb|AAC28635.1| glucose transporter glycoprotein [Homo sapiens] [MASS=					1.97E-04	20.16		37855.1	2 (2 0 0 0 0)
	4521	K.VTILELFR.S	990.59827	-0.00154	2	1.79E-03	3.29	0.50	911.0	13/14
	3889	K.TPEELFHPLGADSQV.-	1639.79626	-0.00215	2	1.97E-04	2.78	0.52	348.4	13/28
95	psu|PF13_0014 | organism=Plasmodium_falciparum_3D7 | product=40S ribosomal protein S7 homologue, putative | locatio					2.69E-06	20.16		22466.6	2 (2 0 0 0 0)
	5192	R.TLTSVYDSILEDIVSPSEIIGK.R	2379.25415	0.00322	2	2.69E-06	3.01	0.52	399.8	16/42
	2660	R.KLINELEK.K	986.58807	-0.00282	2	1.92E-01	2.59	0.10	532.1	13/14
96	psu|PF13_0224 | organism=Plasmodium_falciparum_3D7 | product=60S ribosomal subunit protein L18, putative | location=I					1.74E-05	20.16		21732.7	2 (2 0 0 0 0)
	3044	R.ISEISSSLVR.R	1090.61023	-0.00215	2	1.74E-05	2.62	0.34	864.5	14/18
	4047	K.EGAIAQLYSEMAGR.H	1495.72095	0.00102	2	4.82E-05	2.55	0.59	481.8	14/26
97	psu|PF11_0433 | organism=Plasmodium_falciparum_3D7 | product=hypothetical protein | location=MAL11:1681865-169008					1.14E-06	20.16		324624.2	2 (2 0 0 0 0)
	4679	R.VDLISGYILDIAGK.G	1476.83081	0.00029	2	1.14E-06	3.11	0.50	1107.8	19/26
	1681	K.HMNNNNNNNNNSNVVYNTR.T	2246.97046	0.00406	3	4.34E-05	2.99	0.53	801.4	24/72
98	psu|PFC1020c | organism=Plasmodium_falciparum_3D7 | product=40S ribosomal protein S3A, putative | location=MAL3:96					2.46E-05	20.14		30028.3	2 (2 0 0 0 0)
	2729	R.KGYTLIEGHTDVK.T	1460.77441	-0.00019	2	2.46E-05	2.86	0.31	1221.0	19/24
	4023	K.IYPLQNVLIR.K	1228.74121	0.00029	2	1.70E-03	2.13	0.20	310.7	14/18
99	psu|PFF1470c | organism=Plasmodium_falciparum_3D7 | product=DNA polymerase epsilon, catalytic subunit a, putative | lo					3.62E-04	20.14		344387.9	2 (2 0 0 0 0)
	4609 - 4611	K.IPLYFDFLSR.Y	1270.68298	-0.00032	2	7.77E-03	2.82	0.52	472.4	13/18
	4939	R.ISNIPLFNLFNVQNK.N	1760.96936	-0.00081	2	3.62E-04	2.52	0.45	673.8	18/28
100	gi|1174412|sp|P02549|SPTA1_HUMAN Spectrin alpha chain, erythrocyte (Erythroid alpha-spectrin)					8.08E-06	20.14		279742.3	2 (2 0 0 0 0)
	4637	K.ITDLEHFAESLIADEHYAK.E	2202.07129	-0.00279	4	8.08E-06	2.79	0.41	547.9	37/108
	4946	R.ADMEAEAPTFQALEDFSAELIDSGHHASPEIEK.K	3585.63257	0.01235	4	1.82E-04	2.34	0.38	224.0	32/192
101	psu|PF11_0071 | organism=Plasmodium_falciparum_3D7 | product=RuvB DNA helicase, putative | location=MAL11:255714					2.06E-06	20.14		53372.5	2 (2 0 0 0 0)
	4844	R.AIESPLAPILIMATNR.G	1709.96179	0.00249	2	2.06E-06	2.52	0.46	542.7	17/30

	4729	R.EASLFLVDLIK.Q	1247.72461	-0.00081	2	3.63E-01	2.37	0.35	575.9	12/20
102	psu|PF07_0040 | organism=Plasmodium_falciparum_3D7 | product=lysophospholipase-like protein, putative | location=MAL					7.46E-04	20.13		42330.6	2 (2 0 0 0 0)
	4426	K.ELYTLDDMDHLLPMEPGNER.V	2388.08472	0.00095	3	7.46E-04	2.66	0.37	348.7	20/76
	3868	K.YFYIPLAK.F	1014.56586	-0.00057	2	2.79E-01	2.07	0.43	197.7	8/14
103	gi|88180|pir||C28894 myeloperoxidase (EC 1.11.1.7), splice form H14 - human [MAS					7.40E-04	20.13		92378.3	2 (2 0 0 0 0)
	2864	R.QNQIAVDEIR.E	1185.62219	-0.00312	2	7.40E-04	2.60	0.36	620.2	16/18
	3141	R.DHGLPGYNAWR.R	1285.60730	-0.00093	2	3.05E-02	2.43	0.42	344.5	13/20
104	psu|PF07_0042 | organism=Plasmodium_falciparum_3D7 | product=hypothetical protein, conserved | location=MAL7:51848					3.42E-04	20.13		350482.6	2 (2 0 0 0 0)
	2169	K.NDQNGYINEEEKNETK.T	1924.85193	-0.00259	3	2.31E-01	2.53	0.31	442.3	19/60
	1535	K.NYPNTQNNDNTYK.S	1585.68774	-0.00007	2	3.42E-04	2.07	0.41	86.5	11/24
105	psu|PFB0915w | organism=Plasmodium_falciparum_3D7 | product=liver stage antigen 3 | location=MAL2:796750-801584(+					6.10E-04	20.12		175551.9	2 (2 0 0 0 0)
	2049 - 2051	K.KVEEGVSGLK.K	1045.58875	-0.00368	2	4.47E-02	2.36	0.20	470.2	12/18
	2492	K.NNNVIEVTNK.A	1144.59570	-0.00361	2	6.10E-04	2.23	0.28	661.9	13/18
106	psu|PF11_0053 | organism=Plasmodium_falciparum_3D7 | product=PfSNF2L | location=MAL11:182384-187224(-) | length=					3.61E-11	16.24		167335.5	2 (1 0 1 0 0)
	4467	K.IFDNSEEFDNLFNISK.I	1931.90210	0.00359	2	3.61E-11	4.76	0.55	1978.9	22/30
	3107	K.YDQIKGGVRLFDK.I	1538.83252	-0.09859	2	2.85E-01	2.21	0.50	590.9	14/24
107	psu|PFB0960c | organism=Plasmodium_falciparum_3D7 | product=Plasmdoium falciparum Maurer's Cleft 2 transmembrane					5.01E-08	16.23		14319.7	2 (0 2 0 0 0)
	4573	K.KTNLDPQTSSLVRLVDNMNITQEK.K	2744.42480	-0.04574	3	1.13E-03	4.63	0.60	1117.9	27/92
	4693	K.TNLDPQTSSLVRLVDNMNITQEK.K	2616.32983	-0.04513	3	5.01E-08	4.05	0.46	800.1	25/88
