# Supplementary material for: A Major Role for the Plasmodium falciparum ApiAP2 Protein PfSIP2 in Chromosome End Biology
Source: PLoS Pathog. 2010 Feb 26;6(2):e1000784. doi: 10.1371/journal.ppat.1000784 (PMC2829057; doi:10.1371/journal.ppat.1000784)
Supplement: Table S2 — SEQUEST search results of peptide tandem mass spectra for proteins eluted with SPE2M. (0.08 MB PDF) [file ppat.1000784.s006.pdf]

Table S2: SEQUEST search results

SPE2M affinity purification (control); 2 unique peptides; orange: detected exclusively in the SPE2M sample; grey: ribosomal proteins

	Reference					P (pro)	Score	Coverage	MW	Peptide (Hits)
	Scan(s)	Peptide	MH+	DeltaM	z	P (pep)	XC	DeltaCn	Sp	Ions
1	psu|PFI1445w | organism=Plasmodium_falciparum_3D7 | product=High molecular weight rhoptry protein-2 | location=M					3.77E-11	340.31		162560.6	34 (34 0 0 0 0)
	3786	K.HLINADDVSPSYINLYR.S	1990.00281	0.00261	2	3.77E-11	6.26	0.64	2893.4	28/32
	4766	R.LFVTEGTLEYLLLDK.I	1753.96228	0.00212	2	5.31E-07	6.01	0.49	2677.1	25/28
	2849	K.SLYGNNNNNNAGSESDVTLK.S	2110.96362	-0.00141	2	9.76E-10	5.55	0.54	1055.3	23/38
	2581	K.GSQNTEGESEVPSDDEINK.T	2034.87341	0.03228	2	2.14E-09	5.45	0.50	1164.3	26/36
	1477	K.YGAEQGVGSADSNTK.L	1483.66589	0.00066	2	8.19E-10	5.30	0.57	1813.8	25/28
	3062	K.NSQNYSLTHTEEMIK.I	1794.83264	-0.00325	2	1.41E-06	5.13	0.47	1065.8	18/28
	4569 - 4571	K.SPDSLDFSIPMNFFNHK.N	1995.92688	0.00043	3	2.10E-07	5.03	0.44	1441.3	30/64
	4694 - 4697	R.VTDAIDVYDFFYIK.K	1708.84692	0.00152	2	1.40E-07	4.87	0.57	1772.7	21/26
	3471	K.KDFTQTNALTNLPNLDNKK.T	2175.14038	0.00260	3	1.91E-03	4.86	0.42	1338.0	34/72
	3749	K.NNDQLTFLETQVAK.I	1620.82275	-0.02973	2	5.90E-07	4.76	0.61	1725.3	21/26
	3621	K.KDFTQTNALTNLPNLDNK.K	2047.04541	0.02298	3	1.42E-02	4.52	0.41	924.6	30/68
	4801	K.NFFSELWQNIR.F	1453.72229	0.00163	2	4.71E-05	4.43	0.54	1679.0	17/20
	3689	K.NIVSDALTSEEIK.R	1418.73730	-0.01545	2	2.01E-05	4.13	0.57	1204.7	19/24
	3510	K.NIVSDALTSEEIKR.H	1574.83838	0.00481	2	2.99E-07	4.03	0.53	1733.4	20/26
	4058	K.VFNGIPAFLDK.N	1220.66736	-0.00166	2	4.28E-06	3.98	0.46	841.6	18/20
	4112	K.LFEQIVDQIK.Y	1232.68848	-0.00057	2	5.10E-03	3.94	0.18	1926.4	16/18
	3663	K.QPYYLMSNVPLNNPK.K	1777.89417	0.00309	2	6.12E-04	3.83	0.49	717.0	17/28
	3858	K.DFTQTNALTNLPNLDNK.K	1918.95044	-0.00056	2	2.37E-06	3.77	0.58	1176.4	21/32
	3910	K.VHDFLVLTSQSNENLLPNK.E	2168.13452	0.00156	3	6.60E-04	3.76	0.34	703.0	30/72
	3285	K.IDDINNLNIYEHK.E	1600.79651	-0.00082	3	6.00E-04	3.72	0.34	1485.8	29/48
	3955 - 3957	K.YFDEYFFASGGK.I	1430.62634	0.00115	2	7.26E-03	3.60	0.57	831.9	16/22
	4534	K.EGNEFLMSILHMK.S	1548.75488	0.00115	2	2.05E-04	3.27	0.55	562.0	16/24
	3969	K.YIISDDLYYVNMK.V	1636.79272	0.00530	2	2.07E-06	3.27	0.57	1511.5	19/24
	4470	K.MQVLFITLR.D	1120.65466	-0.00141	2	1.98E-03	3.22	0.27	914.7	14/16
	3937	K.YNFLDIYK.Q	1075.54590	-0.00179	2	5.37E-03	3.22	0.16	751.8	13/14
	3886	K.YMTIYINEAYIK.N	1521.76575	0.00212	2	2.25E-04	3.09	0.55	1184.0	17/22
	3722	K.YLYMDEYLSEGDK.A	1625.70398	0.00139	2	5.38E-07	3.01	0.60	1247.3	19/24
	2842	K.YLLDNSNDIKK.V	1322.69507	-0.00252	2	5.96E-05	2.99	0.36	1883.5	18/20
	5849 - 5851	R.FVYASYILGLVFFIESHIDIAR.L	2573.38037	0.00318	3	2.78E-07	2.95	0.58	420.4	26/84
	4124	R.SNNIVALYILK.T	1247.73584	0.00005	2	4.14E-03	2.77	0.40	775.0	16/20
	3361	K.ADSDITYFVK.Q	1158.56775	-0.00276	2	1.04E-03	2.77	0.39	796.0	14/18
	3223	K.LPLEDYYK.L	1040.52991	-0.00118	2	3.22E-03	2.74	0.45	491.2	13/14
	4065	K.ELETILNNSPFSEEQTMK.L	2110.00098	0.00224	2	9.64E-07	2.74	0.51	252.2	12/34
	2221	R.KYVTTLTK.A	953.56659	0.01531	2	5.50E-03	2.29	0.36	332.1	10/14
2	psu|PF10_0115 | organism=Plasmodium_falciparum_3D7 | product=QF122 antigen | location=MAL10:455397-458816(					5.02E-13	170.36		131545.0	17 (17 0 0 0 0)
	4910	K.NLEEIEEANTYINYLEEQLSITK.N	2756.35132	0.00394	3	6.36E-11	7.22	0.60	2397.4	38/88
	1581	K.NNNNNNNIISNNNNNNNNNSDVKK.D	2714.22217	0.00894	3	2.08E-07	5.18	0.56	762.5	28/92

<table>
<tr><td></td><td>2006</td><td>R.SKEEMSNNNNNNNSSSNGGAFNNSYHESR.Y</td><td>3203.30640</td><td>0.00063</td><td>4</td><td>5.02E-13</td><td>5.00</td><td>0.56</td><td>798.8</td><td>36/168</td></tr>
<tr><td></td><td>4639</td><td>K.IEIENFISTINSTISR.F</td><td>1836.97021</td><td>0.00152</td><td>2</td><td>1.43E-08</td><td>4.88</td><td>0.61</td><td>1825.9</td><td>24/30</td></tr>
<tr><td></td><td>2826</td><td>K.TSHVAQIYGHEDNIHLAK.D</td><td>2033.01990</td><td>-0.00486</td><td>4</td><td>5.40E-11</td><td>4.52</td><td>0.57</td><td>1617.5</td><td>41/102</td></tr>
<tr><td></td><td>4506</td><td>K.KIEIENFISTINSTISR.F</td><td>1965.06519</td><td>0.00088</td><td>3</td><td>1.25E-05</td><td>4.37</td><td>0.57</td><td>570.2</td><td>26/64</td></tr>
<tr><td></td><td>3679 - 3681</td><td>K.DVLENLVQSEGK.E</td><td>1330.68494</td><td>0.00114</td><td>2</td><td>4.93E-06</td><td>4.25</td><td>0.58</td><td>2100.2</td><td>19/22</td></tr>
<tr><td></td><td>4551</td><td>K.ALEMIEELLK.K</td><td>1188.65442</td><td>-0.00020</td><td>2</td><td>4.05E-04</td><td>3.84</td><td>0.33</td><td>1327.2</td><td>15/18</td></tr>
<tr><td></td><td>3445</td><td>K.QNVDITEADIIR.K</td><td>1386.72229</td><td>0.02202</td><td>2</td><td>2.11E-05</td><td>3.68</td><td>0.48</td><td>1144.4</td><td>19/22</td></tr>
<tr><td></td><td>1917</td><td>K.NNNNNNNIISNNNNNNNNNSDVK.K</td><td>2586.12720</td><td>0.00200</td><td>2</td><td>4.13E-07</td><td>3.59</td><td>0.59</td><td>607.7</td><td>20/44</td></tr>
<tr><td></td><td>2975</td><td>K.EIETNTNTLIR.M</td><td>1303.68518</td><td>-0.00300</td><td>2</td><td>1.72E-03</td><td>3.32</td><td>0.46</td><td>578.7</td><td>15/20</td></tr>
<tr><td></td><td>4800</td><td>K.DDIEQFISYLQNVNFNSENK.N</td><td>2417.12549</td><td>0.00253</td><td>3</td><td>1.16E-05</td><td>3.08</td><td>0.41</td><td>704.7</td><td>24/76</td></tr>
<tr><td></td><td>4188 - 4190</td><td>K.NLNLFIIR.Q</td><td>1002.60950</td><td>-0.00081</td><td>2</td><td>1.10E-01</td><td>2.96</td><td>0.23</td><td>803.9</td><td>13/14</td></tr>
<tr><td></td><td>3521</td><td>K.ALEILEYSK.N</td><td>1065.58264</td><td>-0.00154</td><td>2</td><td>5.69E-04</td><td>2.45</td><td>0.37</td><td>621.6</td><td>12/16</td></tr>
<tr><td></td><td>3006</td><td>K.YFVYVDNNK.L</td><td>1161.55750</td><td>-0.00203</td><td>2</td><td>7.12E-04</td><td>2.30</td><td>0.46</td><td>678.8</td><td>13/16</td></tr>
<tr><td></td><td>1710</td><td>K.LSSADMENK.C</td><td>994.45099</td><td>-0.00136</td><td>2</td><td>3.78E-02</td><td>2.08</td><td>0.12</td><td>619.8</td><td>13/16</td></tr>
<tr><td></td><td>3801</td><td>K.NLDSEFVPFEEK.E</td><td>1453.68457</td><td>0.00017</td><td>2</td><td>3.02E-05</td><td>2.07</td><td>0.51</td><td>467.1</td><td>12/22</td></tr>
<tr><td>3</td><td colspan="4">gi|81175178|sp|P35527|K1C9_HUMAN Keratin, type I cytoskeletal 9 (Cytokeratin-9) (CK-9) (Keratin-9) (K9)</td><td></td><td>4.40E-12</td><td>160.33</td><td></td><td>62091.8</td><td>16 (16 0 0 0 0)</td></tr>
<tr><td></td><td>4673</td><td>K.NYSPYYNTIDDLKDQIVDLTVGNNK.T</td><td>2902.41040</td><td>0.00589</td><td>3</td><td>4.40E-12</td><td>6.56</td><td>0.51</td><td>992.9</td><td>39/96</td></tr>
<tr><td></td><td>1349</td><td>R.GGSGGSYGGGGSGGGYGGGSGSR.G</td><td>1791.72766</td><td>0.00151</td><td>2</td><td>4.51E-12</td><td>6.36</td><td>0.71</td><td>1534.9</td><td>24/44</td></tr>
<tr><td></td><td>2906</td><td>K.VQALEEANNDLENK.I</td><td>1586.76562</td><td>-0.00031</td><td>2</td><td>4.33E-07</td><td>5.59</td><td>0.45</td><td>2728.8</td><td>23/26</td></tr>
<tr><td></td><td>1330</td><td>R.GSRGGSGGSYGGGGSGGGYGGGSGSR.G</td><td>2091.88232</td><td>0.00315</td><td>3</td><td>1.05E-06</td><td>5.58</td><td>0.62</td><td>2456.5</td><td>44/100</td></tr>
<tr><td></td><td>1969</td><td>R.SGGGGGGGLGSGGSIR.S</td><td>1232.59778</td><td>0.02434</td><td>2</td><td>4.14E-05</td><td>4.38</td><td>0.45</td><td>1779.2</td><td>24/30</td></tr>
<tr><td></td><td>3823 - 3825</td><td>K.TLNDMRQEYEQLIAK.N</td><td>1851.92688</td><td>0.00206</td><td>2</td><td>2.25E-06</td><td>3.81</td><td>0.39</td><td>1408.2</td><td>19/28</td></tr>
<tr><td></td><td>2974</td><td>R.QGVDADINGLR.Q</td><td>1157.59094</td><td>-0.00447</td><td>2</td><td>2.52E-04</td><td>3.61</td><td>0.48</td><td>1008.7</td><td>18/20</td></tr>
<tr><td></td><td>3767</td><td>R.GGGGSFGYSYGGGSGGGFSASSLGGGFGGGSR.G</td><td>2705.16113</td><td>-0.00015</td><td>3</td><td>2.54E-06</td><td>3.60</td><td>0.49</td><td>1046.2</td><td>36/124</td></tr>
<tr><td></td><td>3850</td><td>R.LASYLDKVQALEEANNDLENK.I</td><td>2377.18823</td><td>0.01108</td><td>3</td><td>1.52E-03</td><td>3.30</td><td>0.33</td><td>581.5</td><td>25/80</td></tr>
<tr><td></td><td>2045</td><td>K.STMQELNSR.L</td><td>1065.49939</td><td>-0.00593</td><td>2</td><td>1.01E-04</td><td>3.18</td><td>0.34</td><td>863.6</td><td>13/16</td></tr>
<tr><td></td><td>3273</td><td>K.TLLDIDNTR.M</td><td>1060.56335</td><td>-0.00202</td><td>2</td><td>2.73E-03</td><td>2.89</td><td>0.42</td><td>674.9</td><td>14/16</td></tr>
<tr><td></td><td>1654</td><td>R.FSSSSGYGGGSSR.V</td><td>1235.52869</td><td>-0.00130</td><td>2</td><td>1.13E-07</td><td>2.80</td><td>0.63</td><td>1248.2</td><td>18/24</td></tr>
<tr><td></td><td>2338</td><td>K.IGLGGRGGSGGSYGR.G</td><td>1350.68726</td><td>0.00053</td><td>2</td><td>2.46E-02</td><td>2.74</td><td>0.32</td><td>548.3</td><td>16/28</td></tr>
<tr><td></td><td>3170</td><td>R.IKFEMEQNLR.Q</td><td>1307.67761</td><td>0.00129</td><td>3</td><td>1.02E-04</td><td>2.70</td><td>0.23</td><td>1245.4</td><td>20/36</td></tr>
<tr><td></td><td>3298</td><td>R.MTLDDFR.I</td><td>897.41345</td><td>-0.00080</td><td>2</td><td>4.83E-03</td><td>2.37</td><td>0.47</td><td>686.9</td><td>11/12</td></tr>
<tr><td></td><td>4925</td><td>K.NYSPYYNTIDDLKDQIVDLTVGNNKTLLDIDNTR.M</td><td>3943.95605</td><td>0.00576</td><td>4</td><td>3.90E-03</td><td>2.16</td><td>0.17</td><td>227.4</td><td>33/198</td></tr>
<tr><td>4</td><td colspan="5">psu|PFD0685c | organism=Plasmodium_falciparum_3D7 | product=chromosome associated protein, putative | location=</td><td>2.42E-08</td><td>160.29</td><td></td><td>141136.4</td><td>16 (16 0 0 0 0)</td></tr>
<tr><td></td><td>4790</td><td>K.LESLQEEFENFLEYK.K</td><td>1917.91162</td><td>0.00261</td><td>2</td><td>2.42E-08</td><td>5.75</td><td>0.54</td><td>2478.2</td><td>23/28</td></tr>
<tr><td></td><td>5021</td><td>R.ENILGFLIDNINVDK.T</td><td>1716.91663</td><td>0.00175</td><td>2</td><td>3.14E-08</td><td>5.70</td><td>0.50</td><td>2839.0</td><td>24/28</td></tr>
<tr><td></td><td>2162</td><td>K.TQDEDNHLNLSNNNK.S</td><td>1755.78918</td><td>-0.00166</td><td>2</td><td>1.64E-07</td><td>4.69</td><td>0.43</td><td>1454.1</td><td>21/28</td></tr>
<tr><td></td><td>2633</td><td>K.SHIEELNNNIEK.I</td><td>1439.71252</td><td>0.00689</td><td>2</td><td>4.71E-06</td><td>4.10</td><td>0.56</td><td>1657.0</td><td>19/22</td></tr>
<tr><td></td><td>4203 - 4205</td><td>R.LNILMNDFNELK.K</td><td>1463.75623</td><td>0.00145</td><td>2</td><td>6.05E-05</td><td>3.72</td><td>0.48</td><td>1454.5</td><td>19/22</td></tr>
<tr><td></td><td>3810</td><td>K.LSNMKDEEILNYLK.S</td><td>1709.87781</td><td>0.00113</td><td>3</td><td>4.97E-06</td><td>3.32</td><td>0.23</td><td>952.9</td><td>26/52</td></tr>
<tr><td></td><td>4721</td><td>K.AVDTILENHYFTLIVEDMQTAK.K</td><td>2551.27490</td><td>0.00352</td><td>3</td><td>1.16E-03</td><td>3.18</td><td>0.43</td><td>576.5</td><td>21/84</td></tr>
<tr><td></td><td>3915</td><td>K.NINQINEFILR.V</td><td>1373.75354</td><td>-0.00020</td><td>2</td><td>9.24E-05</td><td>3.12</td><td>0.46</td><td>1257.5</td><td>16/20</td></tr>
<tr><td></td><td>2991 - 2993</td><td>K.VGSTDINSLNDEVK.K</td><td>1490.73328</td><td>-0.00105</td><td>2</td><td>4.94E-06</td><td>3.12</td><td>0.42</td><td>875.6</td><td>17/26</td></tr>
</table>

	2805	K.ISEGTTNLNEIK.C	1318.68494	-0.00215	2	1.09E-03	2.93	0.41	676.7	14/22
	4418 - 4420	K.LTIVPLLNIK.K	1123.74487	0.00237	2	1.16E-04	2.92	0.41	583.4	15/18
	3533	K.NNEINMLLSK.N	1175.60889	0.00310	2	1.06E-01	2.92	0.36	786.1	13/18
	2666	K.YGSEINELNNK.S	1280.61169	0.00091	2	3.43E-04	2.73	0.48	1023.8	18/20
	3250	K.NIYEETQMLK.S	1268.61914	-0.00325	2	3.68E-02	2.61	0.21	411.6	12/18
	2963	K.FNEFNYPNDK.N	1287.56409	-0.00435	2	5.64E-03	2.60	0.30	1207.3	15/18
	4131	K.VLLPQGIQELEEYR.T	1686.90613	0.00224	2	2.03E-04	2.07	0.49	172.7	12/26
5	psu|PFI0265c | organism=Plasmodium_falciparum_3D7 | product=RhopH3 | location=MAL9:270738-274787(-) | length=					2.38E-13	160.29		104789.1	16 (16 0 0 0 0)
	2546	K.STSAASTSDEISGSEGPSTESTSTGNQGEDK.T	3004.26221	0.00712	2	2.38E-13	5.74	0.74	1060.8	24/60
	4281	R.TMYLDFESSDIFSR.E	1710.76794	0.01128	2	1.13E-07	5.53	0.64	2982.3	23/26
	3162	K.EMEELEEAEGTSNLK.K	1708.75818	-0.00166	2	4.67E-08	4.97	0.59	2722.4	23/28
	4524 - 4526	K.VFTALYNFDSFIK.T	1564.80457	0.00194	2	9.64E-06	4.79	0.41	1222.3	19/24
	3213	R.KQDVLYETDKPQTMDEASYEETVDEDAHHVNEK.Q	3893.72949	0.00416	5	2.71E-07	4.61	0.49	1523.6	54/256
	3286	K.TKDQDLEIELYK.Y	1494.76868	0.00139	2	1.34E-06	4.53	0.43	1538.2	19/22
	3406	K.ASLHVPSVLYR.R	1241.70007	0.00208	3	7.75E-03	4.18	0.48	1250.0	25/40
	4007	K.NYLDSVQNLDTECFK.K	1788.81091	0.00139	2	3.30E-09	3.99	0.57	1354.7	22/28
	2303	K.EDNSEIQCQNVR.K	1434.62781	0.00713	2	1.43E-04	3.75	0.46	1188.2	18/22
	3589	K.ELSHNITDFSFK.E	1437.70093	0.00578	2	5.06E-05	3.69	0.49	622.5	19/22
	2877	K.NLYSTVEDEQR.K	1353.62805	-0.00166	2	2.54E-04	3.65	0.45	1261.5	17/20
	3325	K.QDVLYETDKPQTMDEASYEETVDEDAHHVNEK.Q	3765.63452	-0.00390	5	3.94E-04	3.20	0.41	925.7	48/248
	4220	R.LFFTYNFGDVEPQGK.Y	1761.84827	0.00505	2	1.01E-06	2.71	0.50	436.6	14/28
	2785	K.SGAPISPVSVK.E	1041.59387	0.01213	2	4.37E-03	2.59	0.42	321.7	15/20
	3115 - 3117	K.YTYFPVK.E	917.47675	0.00026	2	1.18E-02	2.26	0.39	354.6	10/12
	2655 - 2657	R.YTSLYIHK.F	1024.54626	-0.00062	2	7.72E-02	2.01	0.40	341.5	10/14
6	psu|PFC0120w | organism=Plasmodium_falciparum_3D7 | product=Cytoadherence linked asexual protein, 3.2 | locatio					2.87E-09	158.25		167134.0	16 (15 1 0 0 0)
	2659	K.EANEENTFPEQEEPNR.Q	1932.82056	0.00639	2	8.58E-07	5.01	0.62	785.7	21/30
	4516	K.NITDADDETYIIPTVQSTFHDIVK.Y	2735.34106	0.00303	3	4.03E-06	4.85	0.55	563.9	32/92
	3565	K.YGYLGEVIAAR.L	1211.64185	0.00529	2	6.88E-07	4.50	0.52	1431.7	18/20
	3941	K.SPESFFFTHGLAAEASK.Y	1825.87549	0.00058	3	3.37E-05	4.40	0.63	740.3	25/64
	3161	K.EQSIEIYNSDISDK.I	1640.76501	-0.00056	2	9.85E-05	4.27	0.58	1073.5	19/26
	1410	K.EQTQHVQEQTYER.K	1675.76709	-0.00166	2	2.87E-09	3.74	0.57	1116.5	19/24
	3771	K.TVVTNYWYPSPIK.K	1567.81555	0.00151	2	2.03E-05	3.61	0.47	1476.5	18/24
	3962	K.SALEELNNVFTNK.E	1478.74854	0.00029	2	8.15E-07	3.57	0.62	1773.3	19/24
	2575	K.YHNIYNINNK.L	1292.63818	0.02373	2	9.43E-06	3.26	0.43	1017.2	16/18
	3357	K.DSNLLNNITK.C	1131.60046	-0.00202	2	4.00E-04	2.92	0.42	993.8	15/18
	2554	R.KYMTATPVYK.K	1201.62854	0.03349	2	1.48E-02	2.88	0.38	703.8	14/18
	2635 - 2637	R.EIEIHNSMASR.Y	1286.61572	0.00396	2	2.18E-04	2.85	0.44	449.0	17/20
	3855 - 3857	K.YFINYAIIK.L	1144.64014	-0.00234	2	1.83E-03	2.83	0.35	638.0	15/16
	3459 - 3461	K.HIPNNLVDELEK.L	1420.74304	0.00304	2	6.70E-07	2.56	0.47	293.5	15/22
	3762	R.SYPLSLVLEHK.F	1285.71509	-0.01021	2	3.76E-03	2.56	0.53	227.7	13/20
	3475	K.ILEFSMSDR.F	1097.52954	-0.00129	2	4.14E-04	2.51	0.42	715.5	14/16
7	gi|11935049|gb|AAG41947.1|AF304164_1 keratin 1 [Homo sapiens]					2.22E-15	150.38		66027.0	15 (15 0 0 0 0)

	2481	R.GSYGSGGSSYGSGGGSYGSGGGGGGHGSYGSGSSSGGYR.G	3312.30811	0.00644	3	2.22E-15	7.36	0.64	1418.6	40/152
	1745	R.GGGGGGYGSGGSSYGSGGGSYGSGGGGGGGR.G	2383.95190	0.00249	2	8.60E-12	6.43	0.71	1133.9	24/60
	3347	K.SKAEAESLYQSKYEELQITAGR.H	2501.25171	0.00669	3	2.76E-09	5.96	0.60	1816.3	37/84
	4244	K.NKLNDLEDALQQAKEDLAR.L	2184.12549	0.00284	3	3.64E-07	5.53	0.52	1426.0	34/72
	4942	R.SLDLDSIIAEVKAQYEDIAQKSK.A	2564.34546	-0.00009	3	4.61E-08	5.11	0.58	953.2	33/88
	1990	K.SKAEAESLYQSK.Y	1340.66919	-0.00288	2	3.59E-06	4.50	0.51	1470.6	19/22
	4397 - 4399	R.SLDLDSIIAEVK.A	1302.71509	0.00383	2	1.21E-04	4.05	0.45	1674.5	19/22
	5005	R.SLDLDSIIAEVKAQYEDIAQK.S	2349.21851	0.00664	2	8.06E-06	3.87	0.56	1211.9	21/40
	3166	K.QISNLQQSISDAEQR.G	1716.85107	0.00176	2	2.52E-11	3.85	0.64	1711.7	22/28
	4374	R.THNLEPYFESFINNLRR.R	2150.07788	0.00405	4	2.70E-03	3.21	0.32	711.7	30/96
	2799	R.TNAENEFVTIKK.D	1393.73218	-0.00374	2	8.86E-03	3.17	0.54	1436.6	17/22
	2197	K.AQYEDIAQK.S	1065.52112	0.02885	2	6.18E-03	3.14	0.33	494.5	13/16
	1669	R.LRSEIDNVKK.Q	1201.68994	-0.00521	3	4.21E-01	2.93	0.16	1009.2	20/36
	2987 - 2989	K.IEISELNR.V	973.53131	-0.00160	2	5.92E-03	2.80	0.28	647.6	13/14
	3977	K.SLNNQFASFIDKVR.F	1638.85986	-0.00149	3	2.82E-06	2.68	0.51	517.0	27/52
8	psu|PF14_0316 | organism=Plasmodium_falciparum_3D7 | product=DNA topoisomerase II, putative | location=MAL14:					3.94E-10	140.24		169121.8	14 (14 0 0 0 0)
	3415	R.ISVYNDGEGIPVDIHK.E	1755.89124	-0.00093	2	2.07E-08	4.83	0.60	816.2	21/30
	3370	K.EKDLSYYDFVNK.E	1520.72681	0.00114	2	3.92E-05	3.98	0.35	1405.8	19/22
	3989	K.STLTTTNMTLFDPNLK.L	1796.90991	0.00078	2	1.18E-06	3.91	0.62	1377.6	19/30
	4268	K.EFLEELLTDEK.H	1365.67834	0.00347	2	1.22E-03	3.73	0.31	1730.9	17/20
	1875	K.NNNNNNNNNDEDEIVK.I	1873.79065	-0.00008	2	3.94E-10	3.72	0.45	679.8	19/30
	3257	R.DGDSIDMAFSK.K	1185.50928	-0.00020	2	4.52E-02	3.64	0.50	914.2	15/20
	4406 - 4410	K.GFLSEFVTPIVK.V	1336.75110	0.00310	2	1.37E-03	2.76	0.44	850.2	15/22
	3541	R.IMGLDITDK.N	1005.52850	0.00451	2	1.79E-04	2.76	0.19	383.8	13/16
	3625	R.YGSLMIMTDQDYDGSHIK.G	2073.92554	0.00717	3	1.91E-04	2.76	0.42	625.9	24/68
	4145	K.IMFLWTGDR.D	1138.57141	0.00041	2	2.29E-01	2.72	0.20	539.1	11/16
	4019 - 4021	K.IDNDTLEITELPIK.K	1613.86328	0.00060	2	8.82E-03	2.59	0.38	425.6	14/26
	3642	K.DLSYYDFVNK.E	1263.58923	0.00176	2	7.06E-04	2.57	0.46	832.0	14/18
	3555	K.GLLINMIHK.F	1038.61279	0.00567	2	3.80E-03	2.33	0.19	1104.3	13/16
	3933	R.NITVETMWLK.D	1234.65002	0.00005	2	6.36E-03	2.01	0.37	256.6	10/18
9	psu|PF11_0317 | organism=Plasmodium_falciparum_3D7 | product=structural maintenance of chromosome protein, pu					2.52E-11	120.29		214541.8	12 (12 0 0 0 0)
	2266	K.NMENDKDINNEKENMEK.E	2094.90674	0.01572	3	2.52E-11	5.56	0.39	1693.3	32/64
	3702	K.EESLQFEQDILNQNK.K	1834.88171	-0.00117	2	1.84E-05	4.61	0.52	1705.5	20/28
	2729	K.MNSHIDQQDQNESNVHLNNENIK.K	2721.22803	-0.00450	4	9.60E-06	4.20	0.28	1571.9	41/132
	3602	K.NLNNLNQELNQLR.D	1582.82959	0.00762	2	7.22E-06	3.54	0.49	912.0	20/24
	4426	K.IDNIYEIIEHNLR.D	1641.85950	0.00455	3	2.50E-01	3.23	0.34	545.7	23/48
	4463 - 4465	R.MDFIPYENFVSNLK.K	1716.83020	0.00304	2	1.79E-06	3.16	0.52	489.8	17/26
	3790	K.FMENLNLQIK.E	1249.66089	0.00139	2	3.24E-04	3.08	0.37	999.3	16/18
	2766	K.LAYDQLNER.I	1121.55859	-0.00239	2	8.85E-05	2.96	0.47	921.9	15/16
	4822	K.IHIDSTNNYENPYENYNIIEFIENIDEYKK.C	3734.74976	-0.00670	4	4.61E-03	2.70	0.25	794.5	35/174
	2274	K.LNINNHDDMK.I	1213.56299	0.00383	2	6.12E-03	2.66	0.36	710.7	14/18
	2115	K.ESNEQIDNIQK.I	1317.62805	-0.00166	2	1.57E-03	2.48	0.32	848.1	15/20

	1689	K.VMENYEQK.I	1040.47168	-0.00105	2	1.57E-03	2.08	0.39	501.7	12/14
10	gi|40354192|ref|NP_000412.2| keratin 10 [Homo sapiens]					4.23E-11	120.27		58791.5	12 (12 0 0 0 0)
	3453	K.GSLGGGFSSGGFSGGSFSR.G	1707.77209	0.00567	2	4.23E-11	5.32	0.66	1517.8	23/36
	4973	K.TIDDLKNQILNLTTDNANILLQIDNAR.L	3052.62720	0.00626	3	6.83E-04	4.58	0.46	1614.3	32/104
	2795	R.ALEESNYELEGK.I	1381.64819	-0.00239	2	1.53E-05	4.46	0.60	2192.7	19/22
	5234	R.VLDELTLTKADLEMQIESLTEELAYLKK.N	3236.72217	-0.00010	4	1.43E-07	4.06	0.47	734.9	39/162
	2199	R.SQYEQLAEQNRK.D	1493.73425	0.00383	2	5.04E-04	3.77	0.44	1273.7	17/22
	2446	R.SLLEGEGSSGGGGR.G	1262.59717	-0.00142	2	1.32E-06	3.77	0.48	1372.6	21/26
	2645	R.LENEIQTYR.S	1165.58484	0.00127	2	3.16E-04	3.69	0.14	1153.3	15/16
	5072	K.ADLEMQIESLTEELAYLK.K	2096.04688	0.00552	3	5.02E-05	3.50	0.52	876.8	24/68
	4667	R.NVSTGDVNVEMNAAPGVDLTQLLNNMR.S	2872.39282	0.00242	3	3.95E-05	3.42	0.58	990.8	31/104
	2457	R.SQYEQLAEQNR.K	1365.63928	-0.00178	2	9.53E-05	3.22	0.41	1291.0	17/20
	2542	K.YENEVALR.Q	993.50000	0.00511	2	9.02E-03	3.09	0.32	1000.4	14/14
	3566	R.SQYEQLAEQNRKDAEAWFNEK.S	2584.20630	0.01210	4	1.62E-02	1.96	0.35	181.9	29/120
11	psu|MAL7P1.229 | organism=Plasmodium_falciparum_3D7 | product=Cytoadherence linked asexual protein | location=					1.34E-11	110.32		162677.5	11 (11 0 0 0 0)
	2958	K.NVNENLNGDSNPNDNINQLK.S	2226.03809	-0.00069	2	1.34E-11	4.23	0.62	1250.9	25/38
	5208	K.NVDDILFFADIINIR.K	1777.94836	0.00237	2	6.71E-05	4.06	0.50	1774.2	19/28
	4417	K.HLPNNLLDELLR.Q	1446.80640	0.00688	2	2.20E-06	3.77	0.55	446.0	17/22
	3683 - 3685	R.NILNVVDEVNK.S	1256.68445	-0.00014	2	1.41E-04	3.74	0.49	1201.0	17/20
	3003 - 3005	K.GEAIILGDTK.I	1016.56226	-0.00408	2	2.60E-02	2.95	0.36	1078.6	14/18
	3874	K.EQSIESYNANISDLIK.K	1823.90210	-0.00032	2	3.37E-10	2.91	0.61	912.9	18/30
	3569	K.LILDSLENDK.L	1159.62048	0.00518	2	1.76E-04	2.87	0.39	717.4	16/18
	3246	R.GYLDEAMEAR.I	1154.51465	0.00114	2	3.80E-03	2.80	0.38	698.6	13/18
	4610	K.SINDADDDIYIVPTIQSSFYDIAK.Y	2703.30347	0.00137	3	2.50E-04	2.75	0.37	395.0	22/92
	1850	R.YAMENMHK.I	1023.43866	-0.00099	2	1.46E-03	2.26	0.50	547.2	12/14
	3030	K.IALEELNK.V	929.53021	0.00182	2	8.66E-03	2.25	0.27	540.2	12/14
12	psu|PFF0835w | organism=Plasmodium_falciparum_3D7 | product=hypothetical protein, conserved | location=MAL6:71					1.62E-07	110.28		52947.8	11 (11 0 0 0 0)
	3333	K.IENEASKYDDETIVPLK.S	1963.98584	-0.00119	3	4.07E-07	4.76	0.53	1560.5	31/64
	3717	K.LLINQIQALNK.N	1267.77319	0.02007	2	5.51E-05	4.52	0.43	1630.4	18/20
	3353	K.LGSISQSIDEIK.K	1289.69470	-0.00142	2	7.47E-05	4.18	0.43	1135.7	18/22
	5153	R.EELALIENMLAYLK.K	1649.88184	0.00114	2	1.62E-07	4.13	0.48	2501.7	23/26
	2259 - 2261	K.LKELQNSYQEK.N	1379.71655	0.01055	2	3.10E-04	3.63	0.44	1014.9	16/20
	3303	K.YDDETIVPLK.S	1192.60962	-0.00239	2	2.72E-05	3.35	0.51	1332.7	16/18
	3077	K.QIGFENEEDIEK.K	1450.66968	-0.00008	2	3.07E-03	3.32	0.49	942.1	15/22
	2745	K.MNHYFMER.R	1127.47607	-0.00154	2	8.66E-04	2.88	0.44	811.4	13/14
	2911	K.IEKPDFDAYNEK.L	1468.69543	-0.00007	2	1.24E-04	2.53	0.39	431.8	15/22
	2499	K.NKPLLSSYSK.I	1136.63098	0.00078	2	7.25E-02	2.25	0.37	120.5	10/18
	3698	K.LEDIDVLPYR.E	1232.65210	0.00139	2	1.73E-02	2.17	0.36	469.2	13/18
13	psu|PF13_0305 | organism=Plasmodium_falciparum_3D7 | product=elongation factor 1 alpha | location=MAL13:226523					5.26E-10	90.32		48927.9	9 (9 0 0 0 0)
	4323 - 4325	K.VGYQADKVDFIPISGFEGDNLIEK.S	2654.33472	0.01221	3	8.44E-10	6.31	0.55	1628.8	37/92
	3395	K.EVLEEARPGDNIGFNVK.N	1886.96069	0.00152	2	2.68E-08	5.63	0.56	960.7	19/32
	4808	K.NMITGTSQADVALLVVPAEVGGFEGAFSK.E	2908.47607	0.00376	3	5.26E-10	5.41	0.63	1532.7	41/112

	4383 - 4385	K.VDFIPISGFEGDNLIEK.S	1892.96399	0.00774	2	8.89E-07	5.41	0.60	1068.0	25/32
	3794	K.KPMVVETFTEYPPLGR.F	1863.96729	0.00090	2	3.64E-08	4.71	0.64	1910.3	22/30
	3643	K.FTAQVIILNHPGEIK.N	1679.94788	-0.00851	3	2.84E-07	3.88	0.45	703.5	27/56
	3734	R.YFFTVIDAPGHK.D	1394.71033	-0.00003	3	4.42E-06	3.31	0.49	944.5	27/44
	3225	K.SGDSALVSLEPK.K	1202.62634	0.00017	2	3.01E-04	3.14	0.49	1792.3	18/22
	3018	K.FLNIDSK.I	836.45123	-0.01637	2	1.73E-01	2.04	0.17	503.8	10/12
14	psu|PF14_0102 | organism=Plasmodium_falciparum_3D7 | product=rhoptry-associated protein 1, RAP1 | location=MAL					1.60E-06	78.24		89996.4	8 (7 1 0 0 0)
	4266	R.TMVTIINDYFEAK.K	1544.76648	0.00249	2	7.16E-05	4.79	0.43	1019.6	18/24
	3971	K.YSLNNMEENINILK.N	1694.84180	0.00419	2	1.60E-06	4.12	0.36	1258.0	19/26
	3901	K.FHPNIDYLTLADGYK.L	1766.87476	-0.00021	3	4.03E-05	4.00	0.38	1569.8	28/56
	3819 - 3821	K.TFSGIGFNLTEK.E	1313.67358	0.01214	2	3.80E-05	3.61	0.58	990.7	18/22
	3070	K.TDMLSLQNEESK.I	1394.64673	-0.00240	2	6.58E-04	3.26	0.38	817.2	18/22
	3159	R.ESMISTTFEQQK.E	1428.66748	-0.00056	2	1.74E-05	2.86	0.43	982.3	17/22
	3086	K.LHVITSGLSYK.A	1217.68884	0.03399	2	3.37E-06	2.86	0.43	843.9	16/20
	3454	K.TLEEFLK.K	879.48224	0.02171	2	3.88E-02	2.20	0.10	338.5	11/12
15	psu|PFF1185w | organism=Plasmodium_falciparum_3D7 | product=iswi protein homologue | location=MAL6:999525-10					2.71E-10	70.37		315421.1	7 (7 0 0 0 0)
	3906	R.RPLVDNVQLKPHQEDGVEWLLK.S	2613.41479	-0.00058	4	2.71E-10	7.35	0.46	4028.8	54/126
	3251	R.IDALDQQYEDQLR.K	1606.77075	0.00066	2	4.67E-07	4.53	0.55	2469.4	21/24
	4448	K.SFLTGGAILADEMGLGK.T	1679.86731	-0.00044	2	1.31E-09	4.22	0.64	2746.5	25/32
	3346	K.GVGTTFLSDGSMR.F	1327.63110	-0.00032	2	1.80E-06	4.08	0.59	1513.9	20/24
	1681	K.NISGEEHIESSSK.L	1416.66016	-0.00484	2	6.15E-06	3.93	0.57	1231.3	20/24
	2421	K.SLYSNYQDK.A	1117.51599	-0.00251	2	3.81E-02	2.27	0.26	491.7	12/16
	3222	K.DLQPFVNPR.V	1085.57385	-0.00093	2	4.99E-02	2.09	0.33	414.4	12/16
16	psu|PFE0060w | organism=Plasmodium_falciparum_3D7 | product=hypothetical protein | location=MAL5:64065-65489(					9.55E-09	70.29		48690.3	7 (7 0 0 0 0)
	4337	R.TLILGSFPQAGEILR.E	1614.92139	0.00773	2	2.68E-07	5.71	0.51	1454.0	24/28
	1443 - 1445	K.NHEESNANMNNHNSFNDK.S	2115.85327	-0.00387	3	7.33E-08	4.83	0.64	1620.6	37/68
	3746	K.FSYALDPNDYASIEDK.L	1847.83337	-0.00300	2	9.55E-09	4.13	0.65	1220.4	23/30
	2414	K.NEQDTNNTHTPNHDEYSHNLPK.N	2605.12988	-0.00254	4	9.94E-02	3.62	0.33	572.5	35/126
	2950	K.HLLQQINTYK.H	1257.69495	-0.00301	2	4.00E-06	3.25	0.51	963.6	16/18
	2489	K.HEEVHKPEEFHKPEEFHK.H	2313.10474	0.00467	5	3.75E-02	2.92	0.25	1695.9	45/136
	1733	K.HEENHKPQMVGQAPPEK.E	1955.93921	-0.00132	4	9.07E-02	2.65	0.42	973.7	32/96
17	psu|MAL8P1.105 | organism=Plasmodium_falciparum_3D7 | product=hypothetical protein, conserved | location=MAL8:!					3.05E-07	70.27		133674.9	7 (7 0 0 0 0)
	4934	K.VVNLFDEENIVINETNYIIDHNVK.S	2844.44141	0.00405	4	3.05E-07	4.46	0.39	761.0	38/138
	3335	K.KPNNTTTGFYTPQFHIAK.E	2065.05005	0.00113	3	6.13E-03	4.09	0.44	756.3	27/68
	4902	K.DSHILIFDVLNGDIIYEK.K	2104.09619	0.00127	2	1.25E-06	2.78	0.47	422.3	12/34
	4146 - 4148	K.LNLFSSPYVQELK.K	1537.82605	0.00414	2	4.95E-03	2.69	0.38	272.1	13/24
	3199	K.NQVIGYYLNK.E	1211.64185	-0.00118	2	1.91E-03	2.62	0.38	1038.2	15/18
	3930	K.EIINDFIIK.G	1104.62988	-0.00117	2	7.23E-02	2.11	0.14	647.3	13/16
	3638	K.IAYLPFSNDK.N	1167.60449	0.00274	2	5.40E-04	2.04	0.36	740.2	14/18
18	gi|14277739|pdb|1HYN|P Chain P, Crystal Structure Of The Cytoplasmic Domain Of Human Erythrocyte Band-3 Protei					2.06E-09	70.26		42509.3	7 (7 0 0 0 0)
	3343 - 3345	K.HSHAGELEALGGVKPAVLTR.S	2042.11414	-0.00011	4	2.06E-09	5.25	0.52	2573.2	53/114
	4876 - 4878	K.GTVLLDLQETSLAGVANQLLDR.F	2326.26123	0.00144	3	1.79E-03	5.13	0.53	1968.0	36/84

	4339	R.ADFLEQPVLGFVR.L	1490.80017	0.00725	2	5.02E-06	4.74	0.57	1293.8	19/24
	2614	R.YQSSPAKPDSSFYK.G	1604.75916	0.01408	2	2.51E-07	4.44	0.57	1746.2	21/26
	4254	R.LQEAAELEAVELPVPIR.F	1877.03784	-0.00064	3	1.09E-03	4.06	0.48	1124.7	32/64
	3677	K.IPPDSEATLVLVGR.A	1466.82129	0.00676	2	3.10E-08	3.92	0.59	848.6	23/26
	3526	R.FIFEDQIRPQDR.E	1563.79138	0.00554	2	1.75E-02	2.57	0.21	306.6	12/22
19	gi|112798|sp|P16452|EPB42_HUMAN Erythrocyte membrane protein band 4.2 (Erythrocyte protein 4.2) (P4.2)					6.97E-12	70.23		76924.7	7 (7 0 0 0 0)
	3258	R.VLPTPQTQATQEGALLNK.R	1909.03894	0.00273	2	6.97E-12	4.49	0.57	701.1	23/34
	2351 - 2353	K.VALTAQTGEQPSK.I	1329.70093	0.00029	2	1.59E-03	4.09	0.53	1947.2	19/24
	2662	R.VVTTFASAQGTGGR.L	1351.69641	0.00273	2	7.40E-06	3.19	0.39	1181.1	19/26
	1970	K.YPEGSLQEK.E	1050.51025	-0.00178	2	1.36E-03	2.52	0.33	704.1	14/16
	4777 - 4782	K.DNGIRPPSLETASPLYLLLK.A	2197.22266	-0.00037	3	1.18E-01	2.50	0.30	459.8	22/76
	2881	R.NPPENTFLR.L	1087.55310	-0.00288	2	1.31E-03	2.49	0.35	569.6	12/16
	2610	K.WSQPVHVAR.V	1079.57446	0.00835	2	1.73E-02	2.21	0.33	325.6	11/16
20	psu|PF14_0186 | organism=Plasmodium_falciparum_3D7 | product=hypothetical protein | location=MAL14:797708-799					1.47E-06	70.22		69935.1	7 (7 0 0 0 0)
	2613	K.IINDNSNLSAPEK.V	1414.71729	0.01237	2	5.32E-04	4.44	0.42	1254.6	18/24
	2341	K.NENNMLDTTNQK.D	1421.63257	-0.00142	2	4.38E-03	3.31	0.44	609.7	16/22
	4046	K.INFYFLPNQEK.K	1412.72083	0.00175	2	1.59E-03	3.09	0.49	1088.4	16/20
	4739 - 4741	K.YNIMNIPTMVLLDR.N	1692.88110	-0.00019	2	1.01E-05	3.04	0.49	778.3	19/26
	2831	K.HINNFTYIK.H	1149.60510	-0.00105	2	3.94E-02	2.42	0.33	812.0	15/16
	3894	K.DNNVNVIYIPLDNK.L	1630.84351	0.00102	2	1.47E-06	2.36	0.49	595.5	15/26
	3891	K.FFNFLNK.Y	929.48798	-0.00026	2	2.07E-02	2.29	0.36	460.9	10/12
21	psu|PF08_0032 | organism=Plasmodium_falciparum_3D7 | product=DnaJ protein, putative | location=MAL8:1044885-1					9.62E-08	70.21		76633.7	7 (7 0 0 0 0)
	3355	K.LIESNSINLNMK.N	1375.72498	0.00126	2	5.61E-05	4.16	0.54	1707.1	19/22
	3102	K.YNEIDNISNESIK.K	1538.73328	0.00126	2	9.62E-08	4.07	0.54	2037.8	22/24
	1945	K.TYQNINSSYNK.F	1331.62268	-0.00349	2	4.94E-05	3.51	0.33	1525.8	17/20
	1597	R.KTYQNINSSYNK.F	1459.71753	0.00151	2	1.89E-06	3.18	0.41	947.3	16/22
	2961	K.MLNDALYK.M	967.49170	-0.00258	2	9.25E-04	2.68	0.21	544.0	13/14
	3871 - 3873	K.DIEILNFQK.I	1119.60449	-0.00172	2	8.40E-04	2.27	0.26	976.4	13/16
	3063	K.LYNIINR.T	905.52032	-0.02247	2	1.14E-01	2.02	0.24	559.1	11/12
22	psu|PF14_0368 | organism=Plasmodium_falciparum_3D7 | product=2-Cys peroxiredoxin | location=MAL14:1575927-15					2.96E-09	60.26		21793.3	6 (6 0 0 0 0)
	3849	K.GGIGNIQHTLISDITK.S	1666.91223	0.00212	2	2.96E-09	5.14	0.63	1891.5	21/30
	4341	K.AEAVFADNTFGEVNLHDFIGK.K	2294.10889	0.00778	3	8.83E-04	3.46	0.49	556.9	31/80
	5593 - 5602	R.SYNVLFGDSVSLR.A	1456.74304	0.00135	2	4.02E-05	2.83	0.38	775.6	16/24
	3839	K.QGVVQHLLVNNLAIGR.S	1731.00244	-0.01741	2	6.22E-08	2.60	0.49	363.0	14/30
	3319	R.NVELIGCSVDSK.Y	1263.62488	-0.00118	2	1.29E-03	2.60	0.26	411.7	13/22
	3317	R.AFVLIDK.Q	805.48181	-0.00032	2	6.09E-02	2.52	0.19	445.3	10/12
23	gi|547754|sp|P35908.1|K22E_HUMAN Keratin, type II cytoskeletal 2 epidermal (Cytokeratin-2e) (CK 2e) (K2e) (keratin-					1.13E-11	60.24		65825.4	6 (6 0 0 0 0)
	1375	R.GSSSGGGYSSGSSSYGSGGR.Q	1740.70557	0.00175	2	1.13E-11	4.67	0.69	1783.2	26/38
	1533	R.GGSGGGGSISGGGYGSGGGSGGR.Y	1741.74841	0.00164	2	1.48E-09	4.38	0.73	1038.8	23/44
	2505	R.GFSSGSAVVSGGSR.R	1254.60730	0.00115	2	1.52E-05	4.15	0.59	2187.2	22/26
	1938	R.SKEEAEALYHSK.Y	1391.68018	0.00027	3	4.29E-06	3.34	0.42	1001.0	22/44
	1519	R.YGSGGGSKGGSISGGGYGSGGGK.H	1890.85767	-0.01102	3	2.55E-04	3.24	0.41	977.2	30/88

	3019	R.YLDGLTAER.T	1037.52625	0.00090	2	7.55E-02	2.17	0.14	632.5	13/16
24	psu|PF14_0359 | organism=Plasmodium_falciparum_3D7 | product=hypothetical protein, conserved | location=MAL14:1					7.21E-08	60.24		48438.4	6 (6 0 0 0 0)
	2470	K.QQQQQQQEAYDDEDHQPEMEGGR.V	2774.13428	0.00436	3	8.30E-07	4.71	0.62	416.0	23/88
	5022	R.KLYDEYGEEGLENGEQPADATDLFDFILNAGK.G	3561.65430	0.00380	4	1.81E-06	4.48	0.52	1076.0	37/186
	3577	K.VTLEQLYNGATK.K	1336.71069	0.00408	2	7.21E-08	3.93	0.53	1434.1	19/22
	2443	K.KRGEDIVSEVK.V	1259.69543	-0.00051	3	8.92E-05	3.02	0.25	1250.5	22/40
	3114	R.EVLDEGMPTYK.D	1281.60315	-0.00130	2	2.30E-04	2.59	0.39	355.2	11/20
	3290	R.KIIEVYIPK.G	1102.68701	-0.00483	2	1.13E-02	2.16	0.34	594.8	12/16
25	psu|PF14_0567 | organism=Plasmodium_falciparum_3D7 | product=hypothetical protein | location=MAL14:2433297-24					8.38E-06	60.22		40043.0	6 (6 0 0 0 0)
	4182 - 4184	K.YFNISPLIIDNK.R	1436.77844	0.00072	2	4.39E-03	4.30	0.45	1056.6	18/22
	2599 - 2601	K.NTSIHPVINASR.S	1308.70190	0.01348	2	8.38E-06	3.25	0.40	721.5	18/22
	2317	K.YIDVSNTEK.L	1068.52075	-0.00154	2	1.01E-03	2.57	0.26	743.6	14/16
	1450	K.EKEDDHSSSNVTNEVK.K	1817.81482	-0.00389	4	1.63E-02	2.54	0.13	587.3	27/90
	1567	K.EDDHSSSNVTNEVK.K	1560.67725	-0.00179	2	4.48E-04	2.31	0.34	293.4	16/26
	2562	K.LSEPPQIK.Y	911.51965	0.02111	2	6.22E-02	2.21	0.16	461.3	11/14
26	psu|PF14_0593 | organism=Plasmodium_falciparum_3D7 | product=hypothetical protein | location=MAL14:2524856-25					2.13E-13	50.24		161116.5	5 (5 0 0 0 0)
	1891	K.NDTNNQTNDLNSNKDETSGSQVTNR.F	2766.21582	0.00425	3	2.13E-13	4.55	0.49	1230.6	31/96
	1625	K.KNDTNNQTNDLNSNKDETSGSQVTNR.F	2894.31079	-0.00523	4	3.44E-05	4.15	0.45	1497.1	43/150
	4110	R.IDKPIAIVSVLGDMHTGK.S	1894.04663	-0.00169	4	6.61E-03	3.81	0.48	1372.1	41/102
	4654	K.SYEYEKDEYNEEIIDLLNLYK.T	2683.26611	-0.00143	3	2.28E-02	2.83	0.12	404.7	20/80
	3851	K.LPPLLNEIK.K	1036.64014	-0.00020	2	2.67E-03	2.14	0.30	441.8	14/16
27	gi|27574029|pdb|1N11|A Chain A, D34 Region Of Human Ankyrin-R And Linker [MASS=4					1.39E-08	50.24		46366.4	5 (5 0 0 0 0)
	3155	K.NGASPNEVSSDGTTPLAIAK.R	1928.95593	0.00164	2	1.39E-08	4.77	0.61	1080.6	23/38
	3607	K.LLLENNANPNLATTAGHTPLHIAAR.E	2622.41113	0.01357	4	2.30E-05	4.68	0.45	2360.8	48/144
	4198	R.EGHVETVLALLEK.E	1437.79480	0.00022	3	1.51E-03	4.23	0.24	1110.5	26/48
	2237	K.HGVMVDATTR.M	1086.53601	0.01128	2	1.88E-06	3.06	0.54	1570.7	17/18
	4051	R.LGYISVTDVLK.V	1207.69324	-0.00203	2	1.27E-04	2.87	0.19	682.7	15/20
28	gi|1360744|pir||B35049 ankyrin 1, erythrocyte splice form 3 - human [MASS=203445					1.45E-09	50.21		203319.9	5 (5 0 0 0 0)
	3434	R.ISEILLDHGAPIQAK.T	1604.90063	0.00323	2	1.45E-09	3.76	0.54	1704.0	21/28
	3529	R.TAAVLLQNDPNPDVLSK.T	1794.95959	0.00567	2	1.44E-04	3.31	0.61	650.6	18/32
	2803	R.ELVNYGANVNAQSQK.G	1634.81323	-0.00056	2	1.50E-07	3.27	0.52	1278.9	19/28
	3154	R.EADAATSFLR.A	1080.53198	-0.00093	2	6.14E-05	3.10	0.32	978.8	17/18
	3238	K.MVVELLHK.E	968.55975	-0.00080	2	1.07E-01	2.22	0.45	508.0	12/14
29	gi|4507021|ref|NP_000333.1| solute carrier family 4, anion exchanger, member 1 [Homo sapiens]					9.14E-06	50.21		101727.8	5 (5 0 0 0 0)
	2671 - 2673	K.ASTPGAAAQIQEVK.E	1370.72742	0.00157	2	3.82E-03	4.14	0.48	754.1	19/26
	2787	R.SVTHANALTVMGK.A	1328.69910	-0.00020	2	9.14E-06	3.86	0.53	1696.9	20/24
	3799	R.GWVIHPLGLR.S	1147.67346	0.00054	2	8.25E-03	2.75	0.35	1268.4	15/18
	2566	K.IFQDHPLQK.T	1125.60510	0.02459	2	6.66E-03	2.49	0.23	375.6	12/16
	4538 - 4540	R.VLLPLIFR.N	970.64478	-0.00093	2	4.73E-04	2.05	0.41	560.0	11/14
30	psu|PFB0100c | organism=Plasmodium_falciparum_3D7 | product=knob associated histidine-rich protein | location=MA					1.26E-08	40.29		71259.4	4 (4 0 0 0 0)
	1650	K.GASTTAGSTTGATTGANAVQSK.D	1938.93628	0.00615	2	1.26E-08	5.24	0.66	1234.5	24/42
	3678	R.FPLGMNDEDEEGKEALAIK.D	2106.00586	0.00986	3	3.54E-07	5.04	0.51	1759.3	37/72

	3215	R.FPLGMNDEDEEGK.E	1480.62610	0.00102	2	7.76E-08	4.11	0.64	1700.1	21/24
	1558	K.NAANNGEQVMSR.G	1290.58557	-0.00154	2	5.31E-05	3.69	0.59	1525.1	18/22
31	psu|PFC0110w | organism=Plasmodium_falciparum_3D7 | product=Cytoadherence linked asexual protein 3.1 | location					1.28E-08	40.27		167381.1	4 (4 0 0 0 0)
	2401	K.NENANVNTPENLNK.L	1570.74561	0.00065	2	4.12E-06	5.31	0.46	1589.4	21/26
	3473	K.LLNEYDNIEQLK.S	1491.76892	0.00261	2	4.88E-05	4.46	0.36	1353.2	17/22
	3898	K.TIVTNYWYPSPIK.K	1581.83118	0.00090	2	1.10E-05	3.42	0.52	750.6	16/24
	3271	K.ESSPIGDHGTFFR.K	1449.67566	0.00200	2	1.28E-08	2.59	0.58	303.8	16/24
32	gi|155969697|ref|NP_775109.2| keratin 6C [Homo sapiens]					2.92E-11	40.25		59988.4	4 (4 0 0 0 0)
	5101	R.NLDLDSIIAEVKAQYEEIAQR.S	2418.25098	0.00395	2	2.92E-11	5.04	0.58	1987.5	26/40
	5039	R.NLDLDSIIAEVKAQYEEIAQRSR.A	2661.38428	-0.00143	3	1.99E-02	3.47	0.11	314.0	21/88
	3107	R.AIGGGLSSVGGGSSTIKYTTTSSSSR.K	2418.21069	0.00169	3	1.28E-05	3.44	0.51	1031.1	33/100
	2850	R.RGFSANSARLPGVSR.S	1574.85095	-0.00460	3	4.84E-04	3.26	0.41	839.8	25/56
33	psu|PFI0165c | organism=Plasmodium_falciparum_3D7 | product=DEAD/DEAH box helicase, putative | location=MAL9					4.05E-08	40.25		300733.3	4 (4 0 0 0 0)
	5106	K.ELEDMTINLINELMK.R	1805.90234	0.00225	2	7.76E-07	4.93	0.56	1718.2	22/28
	4522	R.LVDVYNFNNITNLIK.S	1779.96399	0.00298	2	4.05E-08	4.29	0.55	2028.6	22/28
	1511	K.INDNNNNNINCDNTK.N	1719.73511	-0.00069	2	2.33E-07	3.51	0.61	697.7	17/28
	4405	R.SPFLVSLFK.G	1037.60303	0.00334	2	7.22E-04	3.12	0.49	986.9	14/16
34	psu|PF14_0301 | organism=Plasmodium_falciparum_3D7 | product=hypothetical protein | location=MAL14:1260850-12					1.23E-06	40.23		33245.8	4 (4 0 0 0 0)
	4067 - 4069	K.FDNSGISLLDIK.Y	1321.69983	0.00066	2	3.46E-04	4.23	0.34	1664.7	19/22
	4442	K.SINDNAISVEVIQNIMEK.K	2017.02698	0.00334	2	1.23E-06	3.80	0.61	895.8	20/34
	3614	K.LLNKPSLDLLK.N	1253.78271	0.00376	3	3.36E-03	2.64	0.20	434.0	23/40
	2775	K.FELEHSSISK.H	1176.58948	-0.00276	2	5.94E-03	2.09	0.40	926.4	14/18
35	gi|4504301|ref|NP_003529.1| histone cluster 1, H4a [Homo sapiens]					1.09E-04	40.22		11360.4	4 (4 0 0 0 0)
	3149	R.ISGLIYEETR.G	1180.62085	-0.00252	2	1.09E-04	4.32	0.49	1197.2	17/18
	2878	R.DNIQGITKPAIR.R	1325.75354	0.01848	2	2.38E-04	3.33	0.24	1080.0	18/22
	3665	K.VFLENVIR.D	989.57782	0.00047	2	4.99E-03	2.74	0.29	570.6	13/14
	1781	R.DAVTYTEHAK.R	1134.54260	-0.00117	2	5.40E-03	2.30	0.52	387.8	11/18
36	psu|PFI0605c | organism=Plasmodium_falciparum_3D7 | product=hypothetical protein, conserved | location=MAL9:551					2.56E-07	40.19		52671.4	4 (4 0 0 0 0)
	3297	K.VNNNLLNSIEK.R	1257.67969	-0.00215	2	1.70E-04	3.14	0.32	867.1	15/20
	3327	K.FKIDGIYDDHDYNK.N	1742.80200	-0.00454	3	3.51E-04	3.09	0.38	815.4	25/52
	2746	K.IDGIYDDHDYNK.N	1467.63867	0.00090	2	2.56E-07	2.81	0.43	1028.5	18/22
	2765	K.LYIDPDNEK.N	1106.53638	-0.00289	2	2.13E-02	2.01	0.15	909.2	13/16
37	psu|PFE0080c | organism=Plasmodium_falciparum_3D7 | product=rhoptry-associated protein 2, RAP2 | location=MAL5					1.27E-06	40.19		46708.8	4 (4 0 0 0 0)
	4272	K.FMENLDLYIMK.E	1416.69019	0.00346	2	1.50E-05	3.71	0.50	1349.7	17/20
	3991	K.SMLSTDDYQSFFK.N	1568.69373	0.00200	2	1.27E-06	3.03	0.57	793.1	18/24
	3301	K.SNPYFIVGSR.V	1139.58435	-0.02693	2	5.22E-05	2.61	0.57	592.6	14/18
	3875 - 3877	K.YTEISVLNYVR.D	1356.71582	-0.00038	2	8.01E-06	2.53	0.59	535.0	16/20
38	psu|PFB0895c | organism=Plasmodium_falciparum_3D7 | product=replication factor C subunit 1, putative | location=MA					1.03E-06	40.18		104119.9	4 (4 0 0 0 0)
	2689	R.LPNQENLYDK.L	1233.61096	-0.00337	2	7.65E-05	3.58	0.39	777.8	16/18
	2354	K.LLPQTDQTQENDK.T	1529.74414	0.00066	2	1.03E-06	3.01	0.53	394.8	20/24
	2571	K.AFELQQQNK.S	1105.56360	0.02165	2	4.83E-03	2.91	0.32	1149.8	13/16
	4884	K.WNVFSQIAHDLSLADK.I	1843.93372	-0.00162	3	8.19E-04	2.70	0.41	462.0	21/60

39	psu|MAL7P1.5 | organism=Plasmodium_falciparum_3D7 | product=Plasmodium falciparum Maurer's Cleft 2 transmemb					4.07E-07	30.27		27539.4	3 (3 0 0 0 0)
	4754 - 4756	K.NPDDQISDLVSLVDNMNITQEK.K	2488.18726	0.00299	3	1.19E-05	5.49	0.52	1378.6	34/84
	4542	K.KKNPDDQISDLVSLVDNMNITQEK.K	2744.37720	0.00369	3	1.91E-06	4.84	0.25	1406.8	31/92
	4656	K.KNPDDQISDLVSLVDNMNITQEK.K	2616.28223	0.00284	3	4.07E-07	4.48	0.15	1291.6	32/88
40	psu|PF14_0445 | organism=Plasmodium_falciparum_3D7 | product=hypothetical protein | location=MAL14:1920913-19					8.73E-07	30.25		20330.7	3 (3 0 0 0 0)
	4732 - 4734	K.FNDIFLFIEDTTK.N	1602.80505	0.00188	2	8.73E-07	5.09	0.55	1939.1	21/24
	2171	K.NLNPYNNNDEEK.Q	1463.63977	-0.00264	2	9.25E-04	2.59	0.33	446.5	15/22
	3927	R.NSTNDLFSSFYEK.F	1551.69617	0.00310	2	1.19E-06	2.00	0.46	252.3	11/24
41	psu|PFD0090c | organism=Plasmodium_falciparum_3D7 | product=hypothetical protein, conserved in P.falciparum | loc					1.31E-05	30.25		49712.6	3 (3 0 0 0 0)
	3615 - 3617	R.FGSYEQVLISQPHEFNER.L	2180.04077	0.00870	3	1.31E-05	4.92	0.49	1826.2	32/68
	1659	K.KLSQDYNDVNK.K	1323.65393	-0.00374	2	3.86E-02	2.95	0.32	1032.6	14/20
	1485	K.LSQDYNDVNKK.F	1323.65393	0.00017	2	1.73E-02	2.86	0.30	1186.3	16/20
42	psu|PF14_0159 | organism=Plasmodium_falciparum_3D7 | product=hypothetical protein, conserved | location=MAL14:(					2.02E-06	30.24		110641.7	3 (3 0 0 0 0)
	4657	R.DWFEEFAPIEVVR.N	1636.80054	0.00066	2	2.02E-06	4.74	0.62	1581.2	20/24
	2050	R.DAQYIQETGSK.M	1239.58521	0.00016	2	4.89E-05	2.88	0.50	1056.0	15/20
	2141	K.STINEDINNK.L	1147.55896	0.01384	2	1.61E-03	2.35	0.38	794.4	15/18
43	psu|PFA0065w | organism=Plasmodium_falciparum_3D7 | product=Plasmdoium falciparum Maurer's Cleft 2 transmeml					1.44E-05	30.24		27542.6	3 (3 0 0 0 0)
	4666	K.KNPDAEISDLVNLVDNMNITQEK.K	2600.28711	0.00063	4	1.44E-05	4.10	0.53	2250.5	42/132
	4799	K.NPDAEISDLVNLVDNMNITQEK.K	2472.19214	0.00229	3	8.68E-04	3.78	0.44	1174.1	30/84
	4514	K.KKNPDAEISDLVNLVDNMNITQEK.K	2728.38208	-0.00083	4	9.69E-05	3.75	0.31	1304.0	36/138
44	psu|PF14_0538 | organism=Plasmodium_falciparum_3D7 | product=hypothetical protein | location=MAL14:2314316-23					2.79E-08	30.23		283024.1	3 (3 0 0 0 0)
	4566	R.NLIISQLIDNINEDR.K	1769.93921	0.00115	2	2.41E-06	4.63	0.52	2047.1	21/28
	3195	K.NNSNNSTLDIINDDR.S	1704.77832	0.00078	2	2.79E-08	4.36	0.54	1653.0	20/28
	3131 - 3133	K.HNYFDVEHNDTNSEIVK.Y	2060.93091	-0.00189	3	4.15E-06	4.15	0.50	1810.4	31/64
45	gi|24430192|ref|NP_005548.2| keratin 16 [Homo sapiens]					3.76E-05	30.22		51236.3	3 (3 0 0 0 0)
	2962	K.TEELNKEVASNSELVQSSR.S	2120.04663	-0.00174	3	3.76E-05	4.50	0.50	1978.2	32/72
	2638	R.QTRPILKEQSSSSFSQGQSS.-	2182.07349	0.00845	3	8.34E-04	3.83	0.49	1089.2	29/76
	1833	R.EVFTSSSSSSSR.Q	1260.57019	-0.00203	2	1.65E-04	3.37	0.48	1206.8	18/22
46	psu|PFE1005w | organism=Plasmodium_falciparum_3D7 | product=40S ribosomal subunit protein S9, putative | locatio					3.83E-04	30.22		22109.3	3 (3 0 0 0 0)
	4624	R.QGLLGENEEKLDYVLGLTLPK.L	2329.26489	0.00181	3	2.37E-03	4.34	0.40	1062.1	29/80
	3425	R.IFQGEALLR.R	1046.59937	-0.00191	2	1.38E-02	2.33	0.27	541.1	12/16
	2583	R.QGLLGENEEK.L	1116.55310	0.01628	2	3.83E-04	2.25	0.26	287.2	13/18
47	psu|MAL13P1.61 | organism=Plasmodium_falciparum_3D7 | product=hypothetical protein, conserved in P. falciparum |					2.90E-06	30.21		28236.9	3 (3 0 0 0 0)
	2983 - 2985	K.VETEQSTPAKPEPTEFVNNDIHQNK.N	2852.36963	0.00258	4	2.90E-06	4.28	0.47	630.0	43/144
	1446	R.KNEITNHYSER.I	1390.67102	-0.00387	3	2.10E-04	2.66	0.31	346.5	17/40
	1679	K.NEITNHYSER.I	1262.57605	0.00090	2	6.05E-06	2.39	0.23	523.0	12/18
48	psu|PF14_0455 | organism=Plasmodium_falciparum_3D7 | product=multidrug resistance protein 2 (heavy metal transpo					2.00E-05	30.21		118936.9	3 (3 0 0 0 0)
	1415	K.IYDNNNNNNNNINSK.I	1878.83252	-0.00581	2	2.00E-05	3.81	0.36	651.0	18/30
	4500	R.NIIGIVPQDTILFNESIK.Y	2014.12195	0.00468	2	5.42E-04	2.82	0.39	300.2	15/34
	1531	K.SNEPHTETNSSIDKDDVNK.N	2129.95825	-0.00369	3	1.82E-02	2.79	0.42	422.5	23/72
49	psu|MAL13P1.171 | organism=Plasmodium_falciparum_3D7 | product=transmembrane protein Tmp21 homologue, puta					3.16E-05	30.21		24979.3	3 (3 0 0 0 0)
	2930	K.TEHLNQGTLYLK.Q	1416.74817	-0.00032	2	3.16E-05	3.94	0.48	1206.7	18/22

	4367 - 4372	K.QILDQMNIFHVNLK.R	1712.91528	0.00349	3	4.89E-04	3.61	0.30	937.2	23/52
	3477	K.LPIFETVNK.H	1060.60376	-0.00032	2	3.59E-03	2.83	0.38	844.2	14/16
50	gi|4503595|ref|NP_000493.1| eosinophil peroxidase [Homo sapiens]					7.92E-05	30.20		80989.3	3 (3 0 0 0 0)
	3697	R.IVYEGGIDPILR.G	1344.75220	0.00273	2	7.92E-05	3.57	0.54	1276.3	16/22
	2773	R.RPLLGASNQALAR.W	1366.79138	0.03388	3	4.11E-04	3.45	0.39	1542.8	27/48
	4251	R.NGFLLPLVR.A	1028.62512	0.00042	2	6.96E-03	3.17	0.37	890.7	14/16
51	psu|PFE1465w | organism=Plasmodium_falciparum_3D7 | product=hypothetical protein, conserved | location=MAL5:11					8.06E-11	30.19		191498.5	3 (3 0 0 0 0)
	5137	K.FDPSNMTILNLIEDIK.W	1862.95679	0.00139	2	8.06E-11	3.77	0.56	1369.4	24/30
	4122	R.VGLLDYLNK.V	1034.58801	-0.00141	2	9.62E-05	2.72	0.15	1027.8	14/16
	1502	K.VTDENDAIDTQNKNEHND.-	2071.87988	-0.00436	3	5.78E-03	2.55	0.39	238.5	19/68
52	psu|MAL8P1.98 | organism=Plasmodium_falciparum_3D7 | product=serine protease, putative | location=MAL8:616354-					2.07E-06	30.19		42818.5	3 (3 0 0 0 0)
	4567	K.VDDLIFLGSGFIYNK.N	1700.88940	-0.00080	2	2.99E-06	3.83	0.52	1896.2	21/28
	3187	K.QGEVVIAYGQIQK.F	1432.77942	0.00285	2	2.07E-06	3.54	0.46	1576.6	19/24
	3554	K.NIALLLQNK.G	1026.63062	0.00480	2	1.28E-02	2.55	0.30	442.2	13/16
53	psu|PF10_0366 | organism=Plasmodium_falciparum_3D7 | product=ADP/ATP transporter on adenylate translocase | lo					1.43E-04	30.18		33704.7	3 (3 0 0 0 0)
	3622	K.MLIQTQDSIPEIK.S	1515.80872	0.00639	2	1.43E-04	3.55	0.43	1293.2	20/24
	1787	R.YDQNTDFSK.F	1117.47961	-0.00166	2	3.75E-03	2.95	0.44	904.4	14/16
	3905	K.EQGVLSLWR.G	1087.58948	-0.00118	2	2.77E-02	2.07	0.21	343.8	9/16
54	psu|PFD1015w | organism=Plasmodium_falciparum_3D7 | product=hypothetical protein, conserved | location=MAL4:10					4.79E-07	30.17		44902.0	3 (3 0 0 0 0)
	3015 - 3017	R.GDINMDQFNEK.K	1310.56812	-0.00093	2	3.26E-05	3.33	0.43	911.4	14/20
	3710	K.LTTVALGLGENPSLR.S	1540.86938	0.00262	2	4.79E-07	3.23	0.49	1160.2	20/28
	3174	K.DFTNYIQR.I	1056.51086	0.03033	2	2.27E-02	2.62	0.37	406.8	10/14
55	psu|PFL1925w | organism=Plasmodium_falciparum_3D7 | product=cell division protein FtsH, putative | location=MAL12					7.92E-04	30.17		100894.9	3 (3 0 0 0 0)
	4879 - 4883	K.LELLEVVDFIK.N	1317.76648	-0.00007	2	7.92E-04	3.34	0.43	1425.3	16/20
	5044	R.FEEIAGIDESKLELLEVVDFIK.N	2536.34326	0.00419	3	2.34E-02	2.59	0.42	686.0	25/84
	1925	K.DNFNNTNYR.T	1157.49707	-0.00276	2	2.91E-03	2.59	0.39	694.3	14/16
56	gi|88180|pir||C28894 myeloperoxidase (EC 1.11.1.7), splice form H14 - human [MAS					3.17E-05	30.17		92378.3	3 (3 0 0 0 0)
	4175	R.IGLDLPALNMQR.S	1340.73547	0.00091	2	3.17E-05	3.33	0.33	553.1	16/22
	3139	R.DHGLPGYNAWR.R	1285.60730	0.00102	2	1.47E-02	2.63	0.42	480.4	15/20
	2870	R.QNQIAVDEIR.E	1185.62219	-0.00264	2	4.91E-03	2.35	0.40	297.5	12/18
57	psu|PF07_0129 | organism=Plasmodium_falciparum_3D7 | product=acyl-coA synthetase, PfACS5 | location=MAL7:142					4.08E-05	30.17		93224.9	3 (3 0 0 0 0)
	4093	K.SGSIFSGYFLEK.E	1334.66272	0.00102	2	4.63E-05	3.31	0.50	1059.3	18/22
	1954	K.TGITEDNYNDR.L	1297.56555	-0.00166	2	4.08E-05	2.79	0.51	900.0	16/20
	1871 - 1873	K.MMETYNSTK.L	1104.46997	0.00096	2	2.35E-04	2.11	0.47	394.7	11/16
58	gi|3387905|gb|AAC28635.1| glucose transporter glycoprotein [Homo sapiens] [MASS=					5.34E-05	30.16		37855.1	3 (3 0 0 0 0)
	4488	K.VTILELFR.S	990.59827	-0.00112	2	7.43E-04	3.27	0.43	937.3	13/14
	3511	R.TFDEIASGFR.Q	1142.54761	-0.00130	2	5.34E-05	2.94	0.55	1050.8	15/18
	3879	K.TPEELFHPLGADSQV.-	1639.79626	0.00042	2	1.60E-02	2.52	0.52	395.4	14/28
59	psu|PFA0195w | organism=Plasmodium_falciparum_3D7 | product=hypothetical protein, conserved | location=MAL1:17					1.92E-04	30.14		67759.6	3 (3 0 0 0 0)
	1838	K.INYDQNYNK.D	1171.53784	-0.00093	2	6.86E-04	2.75	0.41	608.3	13/16
	1878	K.FMNHQINSEK.K	1247.58374	-0.00862	2	4.10E-04	2.69	0.34	581.8	15/18
	1559	K.NLTNQTEEPK.N	1173.57458	-0.00080	2	1.92E-04	2.16	0.25	826.1	14/18

60	psu|PFC1080c | organism=Plasmodium_falciparum_3D7 | product=Plasmodium falciparum Maurer's Cleft 2 transmemb					2.53E-05	28.20		26987.2	3 (2 1 0 0 0)
	4617	K.KNPDDQISSLVSLVDNMNITQEK.K	2588.28711	0.00077	3	1.85E-02	4.01	0.25	659.2	25/88
	2093	K.EKNESINELQK.Y	1331.68018	-0.00337	2	2.53E-05	2.95	0.37	529.6	16/20
	3759	K.EYMDSYLMHLR.M	1457.65515	0.00151	2	1.49E-04	2.62	0.36	390.6	14/20
61	psu|PF10_0130 | organism=Plasmodium_falciparum_3D7 | product=hypothetical protein | location=MAL10:515720-517					7.47E-05	28.18		75420.2	3 (2 1 0 0 0)
	4507	K.NNLINLLYIYK.D	1380.78857	-0.00007	2	7.47E-05	3.35	0.45	1341.8	17/20
	2561	K.LNIQNVK.N	828.49377	0.02056	2	1.41E-01	2.21	0.23	584.2	11/12
	3797	K.FAYFDLTR.N	1032.51489	0.00017	2	5.09E-04	2.09	0.35	637.5	13/14
62	psu|PF10_0104 | organism=Plasmodium_falciparum_3D7 | product=hypothetical protein | location=MAL10:422960-424					8.03E-09	20.29		25661.7	2 (2 0 0 0 0)
	3386	K.WGTGSGNQLVTAVTTNK.N	1733.88171	0.00188	2	8.03E-09	5.83	0.60	1199.5	20/32
	2574	K.NINQNGYLSTSK.S	1338.66479	0.02508	2	1.89E-04	3.69	0.53	1005.5	18/22
63	psu|PF14_0627 | organism=Plasmodium_falciparum_3D7 | product=ribosomal protein S3, putative | location=MAL14:26					6.56E-10	20.29		24652.4	2 (2 0 0 0 0)
	3119	R.ILAEDGYSGVEVR.V	1407.71143	0.00175	2	1.68E-07	4.47	0.59	2193.7	21/24
	4771	K.FINDGVFQAELNEFLAR.I	1982.99707	0.00408	2	6.56E-10	4.31	0.67	1220.2	22/32
64	psu|PF11_0062 | organism=Plasmodium_falciparum_3D7 | product=histone H2B | location=MAL11:226066-226419(+)					2.55E-04	20.25		13117.1	2 (2 0 0 0 0)
	4641	K.SMNIMNSFLVDTFEK.I	1775.83423	0.00090	2	2.55E-04	4.90	0.57	1891.1	21/28
	4009	R.YDSYGLYIFK.V	1268.61975	-0.00105	2	1.56E-03	3.05	0.55	1010.3	15/18
65	gi|9739163|gb|AAF97931.1|AF274874_1 keratin 5 [Homo sapiens]					2.64E-08	20.24		62423.1	2 (2 0 0 0 0)
	5108 - 5114	R.NLDLDSIIAEVKAQYEEIANR.S	2404.23535	0.00168	3	2.64E-08	4.88	0.45	1006.1	34/80
	1989	K.YEELQQTAGR.H	1194.57495	-0.00142	2	7.21E-04	3.34	0.36	1446.6	16/18
66	gi|57864582|ref|NP_001009931.1| hornerin [Homo sapiens]					6.53E-09	20.24		282225.7	2 (2 0 0 0 0)
	1366	R.HGSGSGHSSSYGQHGSGSGWSSSSGR.H	2477.02100	0.00649	4	6.53E-09	4.68	0.60	819.3	40/150
	1425	R.GPYESGSGHSSGLGHQESR.S	1928.84814	-0.00509	3	2.42E-02	2.67	0.38	550.9	24/72
67	psu|PFE0050w | organism=Plasmodium_falciparum_3D7 | product=hypothetical protein | location=MAL5:55841-56872(					1.21E-06	20.22		30696.0	2 (2 0 0 0 0)
	3487	K.DIYKPIQSYANNFSK.V	1787.89624	0.00176	2	1.21E-06	4.38	0.48	831.0	23/28
	4139	R.INNAFNLDILR.T	1302.71643	0.00041	2	2.95E-05	4.26	0.33	1423.3	18/20
68	psu|PFE0810c | organism=Plasmodium_falciparum_3D7 | product=40S ribosomal subunit protein S14, putative | locatio					4.44E-07	20.22		16066.7	2 (2 0 0 0 0)
	2866	K.TPQPETAIVSGPQPK.E	1549.82202	0.00200	2	4.44E-07	4.22	0.60	857.9	20/28
	3025	R.IEDVTPIPTDSTR.K	1443.73254	0.00091	2	3.76E-06	3.92	0.57	993.9	19/24
69	psu|PF13_0268 | organism=Plasmodium_falciparum_3D7 | product=ribosomal protein L17, putative | location=MAL13:2					2.48E-06	20.22		23400.0	2 (2 0 0 0 0)
	4457	K.FLLNILDNVQANAESR.N	1816.95520	-0.00094	3	2.48E-06	4.34	0.40	2530.0	33/60
	2935 - 2937	K.YLNDVIEK.K	993.52515	-0.00484	2	5.61E-03	2.29	0.12	454.5	10/14
70	gi|7245526|pdb|1DEJ|A Chain A, Crystal Structure Of A DictyosteliumTETRAHYMENA CHIMERA Actin (Mutant 646: Q					5.19E-08	20.21		41719.9	2 (2 0 0 0 0)
	3817	K.SYELPDGQVITIGNER.F	1790.89197	0.00175	2	5.19E-08	4.29	0.55	1838.8	26/30
	1841	K.DSYVGDEAQSK.R	1198.52222	-0.00179	2	2.96E-05	2.62	0.11	904.8	16/20
71	psu|PF11_0511 | organism=Plasmodium_falciparum_3D7 | product=hypothetical protein | location=MAL11:1985867-19					6.22E-06	20.21		22898.1	2 (2 0 0 0 0)
	4210	R.ALAINDYIDYLK.E	1411.74670	0.00078	2	6.22E-06	4.28	0.52	1369.5	19/22
	4710	K.LLDLVEQYIYSNIEHEK.K	2106.07544	0.00162	3	1.82E-02	3.69	0.43	375.0	26/64
72	psu|MAL13P1.22 | organism=Plasmodium_falciparum_3D7 | product=DNA ligase 1 | location=MAL13:222018-224922(-					3.53E-05	20.21		104440.1	2 (2 0 0 0 0)
	5071	K.AGTGFSDEILSTLYETLSEK.I	2161.05469	0.00297	2	3.53E-05	4.27	0.58	592.4	17/38
	2715	K.TLVENASYEPSR.R	1365.66443	-0.00276	2	1.90E-04	2.70	0.41	623.1	12/22
73	psu|PF10_0187 | organism=Plasmodium_falciparum_3D7 | product=ribosomal protein L30e, putative | location=MAL10					1.39E-05	20.21		11767.1	2 (2 0 0 0 0)

	3841	R.SVIEYYAMLSK.C	1303.66028	-0.00252	2	1.52E-04	4.19	0.51	1516.3	17/20
	3318	K.LVIVSSNCPSIQR.S	1415.76746	0.00004	2	1.39E-05	3.96	0.58	1436.3	19/24
74	psu|PF11_0465 | organism=Plasmodium_falciparum_3D7 | product=dynamin-like protein | location=MAL11:1800541-18					4.42E-07	20.21		96313.0	2 (2 0 0 0 0)
	4493	R.TIFNEFLDEYVGK.N	1574.77368	0.00127	2	4.42E-07	4.18	0.60	1449.6	19/24
	4860	K.SSVLESIVGMDFLPR.G	1649.85669	0.00237	2	3.49E-04	3.04	0.51	1207.0	19/28
75	psu|PF13_0032 | organism=Plasmodium_falciparum_3D7 | product=hydrolase, putative | location=MAL13:289766-2912					8.05E-07	20.20		56775.0	2 (2 0 0 0 0)
	3509	K.YEEIGIPISNSTVK.G	1549.81079	0.00225	2	1.07E-04	3.04	0.51	668.3	19/26
	3922	R.YIQEALYDPNIQIMK.N	1838.93567	0.00078	2	8.05E-07	2.87	0.43	927.2	17/28
76	gi|119703753|ref|NP_005546.2| keratin 6B [Homo sapiens]					8.85E-07	20.20		60030.3	2 (2 0 0 0 0)
	2893	R.ATGGGLSSVGGGSSTIKYTTTSSSSR.K	2406.17432	0.00034	3	1.82E-06	4.05	0.53	1058.9	34/100
	2687	R.ATGGGLSSVGGGSSTIK.Y	1435.73877	-0.00227	2	8.85E-07	3.82	0.58	1537.7	25/32
77	psu|PF11_0224 | organism=Plasmodium_falciparum_3D7 | product=circumsporozoite-related antigen | location=MAL11					3.08E-04	20.20		17285.0	2 (2 0 0 0 0)
	2495	K.KEEELVEVNK.R	1216.64197	0.00969	2	3.08E-04	4.04	0.27	1432.6	16/18
	2323	K.KEEELVEVNKR.K	1372.74304	0.00055	3	1.58E-03	3.83	0.26	1535.6	25/40
78	psu|PFE0040c | organism=Plasmodium_falciparum_3D7 | product=Mature parasite-infected erythrocyte surface antiger					2.10E-10	20.20		168184.9	2 (2 0 0 0 0)
	4171	K.FSEMNVVHQILNYEER.I	2007.95923	0.00150	3	9.13E-02	4.01	0.47	1311.4	31/60
	2445	K.VSGPYENSENSNVTSESEETKK.L	2415.07935	0.00339	3	2.10E-10	3.67	0.53	1180.3	36/84
79	psu|PF11_0301 | organism=Plasmodium_falciparum_3D7 | product=spermidine synthase | location=MAL11:1121928-1					1.40E-07	20.19		36571.7	2 (2 0 0 0 0)
	3519	K.NVLVVGGGDGGIIR.E	1325.75354	0.00310	2	1.40E-07	3.86	0.52	2108.6	23/26
	3178	R.VNVFIEDASK.F	1121.58374	0.00188	2	4.53E-02	2.19	0.42	419.4	10/18
80	psu|PF11_0374 | organism=Plasmodium_falciparum_3D7 | product=hypothetical protein | location=MAL11:1422741-14					1.24E-05	20.19		128465.1	2 (2 0 0 0 0)
	2179	K.TNEDENAESNLNPNEK.Y	1817.77844	-0.00166	2	1.24E-05	3.81	0.52	980.3	19/30
	2802	K.SNLDSSNLNEINK.N	1447.70227	-0.00081	2	8.39E-05	3.54	0.44	1061.6	18/24
81	gi|4557701|ref|NP_000413.1| keratin 17 [Homo sapiens]					9.44E-06	20.19		48076.1	2 (2 0 0 0 0)
	2441	R.EVATNSELVQSGK.S	1361.69067	-0.00007	2	9.44E-06	3.77	0.59	1136.5	19/24
	1894	K.ATMQNLNDR.L	1062.49963	-0.00007	2	1.33E-02	2.16	0.37	628.3	13/16
82	psu|PFC0730w | organism=Plasmodium_falciparum_3D7 | product=conserved protein, putative | location=MAL3:67113					1.19E-04	20.19		26023.9	2 (2 0 0 0 0)
	3326	K.ISQTATSHLTQITGNLTEK.L	2043.07166	-0.00375	3	1.56E-02	3.75	0.43	999.6	31/72
	3385	R.VFNNIDDYVK.K	1226.60522	-0.00373	2	1.19E-04	3.45	0.48	1181.1	16/18
83	gi|2765839|emb|CAB09722.1| rhesus D category VI type III protein [Homo sapiens]					2.56E-04	20.18		45218.1	2 (2 0 0 0 0)
	3885	K.YFDDQVFWK.F	1247.57312	0.00017	2	2.56E-04	3.61	0.41	865.5	15/16
	3997	K.FPHLAVGF.-	887.47742	-0.00087	2	3.85E-04	2.88	0.52	773.6	13/14
84	psu|PFL1005c | organism=Plasmodium_falciparum_3D7 | product=chromodomain protein | location=MAL12:831245-83					3.44E-08	20.18		30999.3	2 (2 0 0 0 0)
	3183	K.NESPQWVEETNIR.R	1601.75537	0.00591	2	3.44E-08	3.51	0.58	1512.4	20/24
	4129	K.NLHNVGDELYISVIHNINNK.E	2306.18872	0.00161	4	3.83E-02	3.24	0.34	1317.7	37/114
85	psu|PF14_0098 | organism=Plasmodium_falciparum_3D7 | product=hypothetical protein | location=MAL14:392972-393					1.23E-06	20.18		34930.4	2 (2 0 0 0 0)
	3547	K.NLNDNVDVILTK.K	1357.73218	0.00688	2	1.82E-03	3.59	0.48	1393.7	19/22
	3911	K.NYIYNDPSDLLIR.A	1595.80640	0.00078	2	1.23E-06	2.35	0.31	447.8	14/24
86	psu|PFC1020c | organism=Plasmodium_falciparum_3D7 | product=40S ribosomal protein S3A, putative | location=MAL					3.34E-05	20.18		30028.3	2 (2 0 0 0 0)
	2923	K.GYTLIEGHTDVK.T	1332.67944	-0.00044	2	3.34E-05	3.56	0.49	1393.1	19/22
	4013	K.IYPLQNVLIR.K	1228.74121	-0.00020	2	1.43E-02	2.18	0.23	253.7	13/18
87	psu|PF11_0071 | organism=Plasmodium_falciparum_3D7 | product=RuvB DNA helicase, putative | location=MAL11:25					4.21E-04	20.17		53372.5	2 (2 0 0 0 0)

	4793	R.AIESPLAPILIMATNR.G	1709.96179	0.00065	3	1.36E-03	3.50	0.49	778.2	23/60
	4683 - 4685	R.EASLFLVDLIK.Q	1247.72461	-0.00032	2	4.21E-04	2.34	0.52	953.2	15/20
88	psu|PF14_0563 | organism=Plasmodium_falciparum_3D7 | product=DEAD-box RNA helicase, putative | location=MAL1					1.27E-05	20.17		84261.7	2 (2 0 0 0 0)
	4987	K.IDNELIQILTYLK.F	1575.89929	-0.00007	2	1.27E-05	3.43	0.52	1470.6	17/24
	1802	R.IGDTLNNTSNQR.V	1332.65027	-0.00008	2	1.06E-04	2.83	0.39	538.1	13/22
89	psu|MAL8P1.53 | organism=Plasmodium_falciparum_3D7 | product=hypothetical protein, conserved | location=MAL8:9					1.47E-05	20.17		60504.5	2 (2 0 0 0 0)
	3853	R.HNIDLLPIYIHGAK.T	1603.89551	0.00107	3	1.47E-05	3.34	0.59	1143.1	27/52
	3095	K.ELYNGNIVMNNK.G	1408.68896	-0.00166	2	1.45E-01	2.77	0.42	947.6	15/22
90	psu|PF11_0506 | organism=Plasmodium_falciparum_3D7 | product=hypothetical protein | location=MAL11:1950207-19					2.27E-04	20.17		66258.1	2 (2 0 0 0 0)
	1565	K.IKPTYEENDEDK.S	1480.68018	-0.00369	3	1.33E-03	3.33	0.41	878.6	25/44
	1903	K.KDEYDIQSR.V	1153.54834	-0.00105	2	2.27E-04	2.41	0.37	576.2	12/16
91	psu|PF13_0224 | organism=Plasmodium_falciparum_3D7 | product=60S ribosomal subunit protein L18, putative | locati					1.74E-05	20.17		21732.7	2 (2 0 0 0 0)
	3939	R.FPALHLPTLQK.E	1264.74121	-0.00137	3	2.12E-02	3.31	0.38	899.0	23/40
	4031	K.EGAIAQLYSEMAGR.H	1495.72095	0.00029	2	1.74E-05	2.52	0.55	520.7	13/26
92	psu|MAL13P1.50 | organism=Plasmodium_falciparum_3D7 | product=hypothetical protein, conserved | location=MAL13					2.47E-05	20.17		24397.8	2 (2 0 0 0 0)
	3903	K.SNLIVNNIVFK.A	1260.73108	0.00066	2	2.47E-05	3.31	0.49	1005.3	16/20
	3981	R.IYDFLIPK.K	1008.57642	-0.00038	2	4.13E-02	2.60	0.35	501.6	11/14
93	psu|PFF1415c | organism=Plasmodium_falciparum_3D7 | product=DNAJ domain protein, putative | location=MAL6:119					8.32E-05	20.16		44641.0	2 (2 0 0 0 0)
	3534	K.TITQQVAPGFIMQNK.I	1675.88354	0.00774	2	8.32E-05	3.28	0.51	830.9	18/28
	4118	K.FSEIAEAYEILGDEEKR.K	1998.96545	0.00114	3	1.35E-01	2.96	0.42	353.8	22/64
94	psu|PF14_0036 | organism=Plasmodium_falciparum_3D7 | product=acid phosphatase, putative | location=MAL14:1414					9.74E-06	20.16		35800.9	2 (2 0 0 0 0)
	4045	K.FPLDPIQTNQITDLK.N	1742.93237	0.00225	2	2.61E-05	3.23	0.42	1621.5	24/28
	3231 - 3233	K.QITISAVGMQAK.D	1246.68237	-0.00245	2	9.74E-06	2.81	0.50	834.0	17/22
95	psu|PFI0935w | organism=Plasmodium_falciparum_3D7 | product=DNAJ-like molecular chaperone protein, putative | lo					1.07E-04	20.16		43232.6	2 (2 0 0 0 0)
	2813	K.IYHPDKNPDESANSSFIK.L	2061.98755	-0.00108	4	2.05E-04	3.22	0.30	1210.6	39/102
	3243	K.QAYDVLTDDVR.R	1294.62732	-0.00178	2	1.07E-04	2.22	0.45	687.8	17/20
96	psu|PFI1730w | organism=Plasmodium_falciparum_3D7 | product=cytoadherence linked asexual protein 9(CLAG9) | lo					4.05E-04	20.15		160312.3	2 (2 0 0 0 0)
	3291	R.LSVHDEPFLR.F	1212.63721	-0.00095	3	6.15E-04	3.06	0.39	1120.2	22/36
	1757	K.FSQENDPVSK.V	1150.53748	0.00041	2	4.05E-04	2.95	0.38	1096.7	14/18
97	gi|34526448|dbj|BAC85240.1| unnamed protein product [Homo sapiens] [MASS=53025]					5.84E-04	20.15		52992.1	2 (2 0 0 0 0)
	2477	K.VTMQNLNDR.L	1090.53101	0.00029	2	5.84E-04	3.05	0.38	1027.4	14/16
	3287 - 3289	K.DAEAWFNEK.S	1109.48987	-0.00313	2	1.22E-01	2.11	0.39	723.4	12/16
98	psu|PF07_0042 | organism=Plasmodium_falciparum_3D7 | product=hypothetical protein, conserved | location=MAL7:5					5.18E-04	20.15		350482.6	2 (2 0 0 0 0)
	2098	K.NDQNGYINEEEKNETK.T	1924.85193	-0.00186	3	5.96E-02	2.96	0.34	625.9	23/60
	1513	K.NYPNTQNNDNTYK.S	1585.68774	-0.00349	2	5.18E-04	2.34	0.38	118.8	12/24
99	psu|PFF1430c | organism=Plasmodium_falciparum_3D7 | product=amino acid transporter | location=MAL6:1213950-12					7.64E-04	20.14		68815.1	2 (2 0 0 0 0)
	3075	K.SYNNLLINK.E	1078.58911	-0.00032	2	6.82E-02	2.79	0.13	788.0	13/16
	1634	K.NTETYTNVNDK.K	1298.58594	-0.00069	2	7.64E-04	2.12	0.41	251.3	11/20
100	psu|PF13_0014 | organism=Plasmodium_falciparum_3D7 | product=40S ribosomal protein S7 homologue, putative | lo					7.94E-04	20.14		22466.6	2 (2 0 0 0 0)
	5145	R.TLTSVYDSILEDIVSPSEIIGK.R	2379.25415	0.00298	2	7.94E-04	2.64	0.45	340.1	14/42
	4042	K.TILIYIPYK.I	1123.67615	-0.00081	2	1.06E-02	2.07	0.39	542.8	12/16
101	psu|PFL2005w | organism=Plasmodium_falciparum_3D7 | product=replication factor c subunit 4 | location=MAL12:177					5.40E-04	20.13		37613.7	2 (2 0 0 0 0)

	4653	K.IIILDEVDSMTTAAQQSLR.R	2104.09546	0.00186	3	5.40E-04	2.53	0.30	571.4	26/72
	2783	K.IIDALQSR.C	915.52582	0.00993	2	3.05E-03	2.18	0.20	579.4	13/14
102	psu|PF11_0507 | organism=Plasmodium_falciparum_3D7 | product=antigen 332, putative | location=MAL11:1952200-1				3.07E-04	20.12		620797.5	2 (2 0 0 0 0)
	2838	K.NVSEEIPVEEK.N	1272.63184	-0.00203	2	3.07E-04	2.16	0.40	630.9	13/20
	2815	K.SVSEEIPVEEK.S	1245.62085	0.00029	2	1.78E-03	2.15	0.27	657.4	13/20
103	psu|PFL1725w | organism=Plasmodium_falciparum_3D7 | product=ATP synthase beta chain, mitochondrial precursor,				3.02E-04	18.14		58357.6	2 (1 1 0 0 0)
	5213	R.FVELEDTIIGFSELLK.G	1852.99426	-0.00027	3	5.50E-03	2.85	0.15	819.5	23/60
	5086	K.SLQDIIAILGIDELSEQDK.L	2100.10718	-0.00027	3	3.02E-04	2.81	0.45	935.4	24/72
104	psu|PFB0960c | organism=Plasmodium_falciparum_3D7 | product=Plasmdoium falciparum Maurer's Cleft 2 transmemb				4.07E-07	16.19		14319.7	2 (0 2 0 0 0)
	4656	K.TNLDPQTSSLVRLVDNMNITQEK.K	2616.32983	-0.04476	3	4.07E-07	3.82	0.41	939.2	27/88
	4542	K.KTNLDPQTSSLVRLVDNMNITQEK.K	2744.42480	-0.04391	3	5.60E-03	3.61	0.54	762.8	25/92
