# Supplementary material for: A Major Role for the Plasmodium falciparum ApiAP2 Protein PfSIP2 in Chromosome End Biology
Source: PLoS Pathog. 2010 Feb 26;6(2):e1000784. doi: 10.1371/journal.ppat.1000784 (PMC2829057; doi:10.1371/journal.ppat.1000784)
Supplement: Table S3 — Genome-wide prediction of SPE2 consensus motifs. The position of 777 SPE2 consensus motifs experimentally determined by competition EMSA are listed. Column 1: Chromosome ID. Column 2: nucleotide position with respect to the left telomere end of the first bp of the SPE2 motif. Column 3: nucleotide position of the last bp of the SPE2 motif with respect to the left telomere on each chromosome. Column 4: “+” denominates positive association of the corresponding SPE2 motif in the ChIP-on-chip experiment (threshold: min 1,000bp, >1.4 log2 ChIP over input). Column 5: SPE2 orientation on sense (“1”) or antisense (“−1”) strand. Column 6: “tel” indicates SPE2 location in TAREs, “var” indicates SPE2 location upstream of upsB var genes. SPE2 elements associated with genes that do not fall into the first to classes are identified by PlasmoDB annotation of the corresponding gene. Column 7: distance in bp to previous SPE2 motif. Column 8: blank cells represent canonical 4bp spacing between the two half sites of the bipartite SPE2 motif. “5” represents 5bp spacing. Column 9: PlasmoDB accession numbers of SPE2-associated genes. Column 10: SPE2 positions other than in TARE or upstream of coding sequences are indicated. Column 11: orientation of SPE2-associated genes on the sense (“1”) or antisense (“−1”) strand. Column 12: nucleotide position of the ATG start codon of SPE2-associated genes with respect to the left telomere on each chromosome. Values/information in individual columns was retrieved from PlasmoDB version 5.5 (www.plasmodb.org). (0.03 MB PDF) [file ppat.1000784.s007.pdf]

Table S3: Genome-wide SPE2 prediction

chromosome	SPE2 start	SPE2 end	PfnSIP2-HA ChIP-on-chip	plus/minus strand	chromosomal landmark	distance to previous SPE2	5bp spacing	Accession	Comments	plus/minus strand	ATG
chr1	2385	2400	+	-1	TARE						
chr1	2520	2535	+	-1	TARE	120					
chr1	2790	2805	+	-1	TARE	255					
chr1	2925	2940	+	-1	TARE	120					
chr1	3060	3075	+	-1	TARE	120					
chr1	3351	3366	+	-1	TARE	276					
chr1	3486	3501	+	-1	TARE	120					
chr1	3621	3636	+	-1	TARE	120					
chr1	3756	3771	+	-1	TARE	120					
chr1	4047	4062		-1	TARE	276					
chr1	4684	4699		-1	TARE	622					
chr1	4840	4855		1	TARE	141					
chr1	5022	5037	+	-1	TARE	167					
chr1	5367	5382	+	1	TARE	330					
chr1	5497	5513	+	-1	TARE	115	5				
chr1	6210	6225	+	-1	TARE	697					
chr1	6464	6479	+	1	TARE	239					
chr1	7842	7857		1	TARE	1363					
chr1	9452	9467		-1	TARE	1595					
chr1	27336	27352	+	1	var		5	PFA0005w		1	29733
chr1	27338	27354	+	-1	var	-14	5	PFA0005w			
chr1	27394	27410	+	1	var	40	5	PFA0005w			
chr1	27396	27412	+	-1	var	-14	5	PFA0005w			
chr1	27511	27526	+	1	var	99		PFA0005w			
chr1	27538	27553	+	1	var	12		PFA0005w			
chr1	27560	27575	+	1	var	7		PFA0005w			
chr1	27588	27603		1	var	13		PFA0005w			
chr1	612437	612452		-1	var			PFA0765c	exon1		
chr1	618900	618915		-1	var			PFA0765c		-1	616613
chr1	618927	618942		-1	var	12		PFA0765c			
chr1	618949	618964		-1	var	7		PFA0765c			
chr1	618977	618992		-1	var	13		PFA0765c			
chr1	619000	619015		-1	var	8		PFA0765c			
chr1	619026	619041		-1	var	11		PFA0765c			
chr1	619053	619068		-1	var	12		PFA0765c			
chr1	619075	619090		-1	var	7		PFA0765c			
chr1	619097	619112		-1	var	7		PFA0765c			
chr1	619408	619424	+	1	var	296	5	PFA0765c			
chr1	619410	619426	+	-1	var	-14	5	PFA0765c			
chr1	619508	619524	+	1	var	82	5	PFA0765c			
chr1	619510	619526	+	-1	var	-14	5	PFA0765c			
chr10	2015	2030		-1	TARE						
chr10	2150	2165		-1	TARE	120					
chr10	2285	2300		-1	TARE	120					
chr10	2420	2435	+	-1	TARE	120					
chr10	2555	2570	+	-1	TARE	120					
chr10	2690	2705	+	-1	TARE	120					
chr10	3116	3131	+	-1	TARE	411					
chr10	3251	3266	+	-1	TARE	120					
chr10	3677	3692		-1	TARE	411					
chr10	4331	4346		-1	TARE	639					
chr10	4487	4502		1	TARE	141					
chr10	4669	4684		-1	TARE	167					
chr10	5014	5029	+	1	TARE	330					
chr10	5144	5160	+	-1	TARE	115	5				
chr10	5422	5437	+	1	TARE	262					
chr10	6112	6127	+	1	TARE	675					
chr10	8180	8195		1	TARE	2053					
chr10	9799	9814		-1	TARE	1604					
chr10	25587	25602	+	1	var			PF10_0001		1	28491
chr10	25610	25625	+	1	var	8		PF10_0001			
chr10	25638	25653	+	1	var	13		PF10_0001			
chr10	25662	25677	+	1	var	9		PF10_0001			
chr10	25686	25701	+	1	var	9		PF10_0001			
chr10	25710	25725	+	1	var	9		PF10_0001			
chr10	25736	25751	+	1	var	11		PF10_0001			
chr10	25763	25778	+	1	var	12		PF10_0001			
chr10	25790	25805	+	1	var	12		PF10_0001			
chr10	25817	25832	+	1	var	12		PF10_0001			
chr10	25844	25859	+	1	var	12		PF10_0001			
chr10	25871	25886	+	1	var	12		PF10_0001			

chr10	25898	25913	+	1	var	12		PF10_0001			
chr10	25925	25940	+	1	var	12		PF10_0001			
chr10	25952	25967	+	1	var	12		PF10_0001			
chr10	25979	25994	+	1	var	12		PF10_0001			
chr10	26006	26021	+	1	var	12		PF10_0001			
chr10	26033	26048	+	1	var	12		PF10_0001			
chr10	26060	26075	+	1	var	12		PF10_0001			
chr10	26087	26102	+	1	var	12		PF10_0001			
chr10	26114	26129	+	1	var	12		PF10_0001			
chr10	26141	26156	+	1	var	12		PF10_0001			
chr10	26168	26183	+	1	var	12		PF10_0001			
chr10	26195	26210	+	1	var	12		PF10_0001			
chr10	26222	26237	+	1	var	12		PF10_0001			
chr10	26250	26265	+	1	var	13		PF10_0001			
chr10	26277	26292	+	1	var	12		PF10_0001			
chr10	26304	26319		1	var	12		PF10_0001			
chr10	26331	26346		1	var	12		PF10_0001			
chr10	159324	159339		-1	hyp			PF10_0037		-1	158517
chr10	609000	609015		1	FAD synthetase putative			PF10_0147		1	611212
chr10	691351	691366		-1	hyp			PF10_0166		-1	690819
chr10	772648	772663		-1	hyp			PF10_0184		-1	771953
chr10	1652090	1652105	+	-1	var			PF10_0406		-1	1649947
chr10	1652114	1652129	+	-1	var	9		PF10_0406			
chr10	1652141	1652156	+	-1	var	12		PF10_0406			
chr10	1652169	1652184	+	-1	var	13		PF10_0406			
chr10	1652191	1652206	+	-1	var	7		PF10_0406			
chr10	1652213	1652228	+	-1	var	7		PF10_0406			
chr10	1652240	1652255	+	-1	var	12		PF10_0406			
chr10	1652267	1652282	+	-1	var	12		PF10_0406			
chr10	1652294	1652309	+	-1	var	12		PF10_0406			
chr10	1652321	1652336	+	-1	var	12		PF10_0406			
chr10	1652348	1652363	+	-1	var	12		PF10_0406			
chr10	1652375	1652390	+	-1	var	12		PF10_0406			
chr10	1652402	1652417	+	-1	var	12		PF10_0406			
chr10	1652429	1652444	+	-1	var	12		PF10_0406			
chr10	1652735	1652751	+	1	var	291	5	PF10_0406			
chr10	1652737	1652753	+	-1	var	-14	5	PF10_0406			
chr10	1678016	1678031		1	TARE						
chr10	1679643	1679658		-1	TARE	1612					
chr10	1681711	1681726	+	-1	TARE	2053					
chr10	1682119	1682134	+	-1	TARE	393					
chr10	1682464	1682479	+	1	TARE	330					
chr10	1682646	1682661	+	-1	TARE	167					
chr10	1682802	1682817	+	1	TARE	141					
chr10	1683437	1683452	+	1	TARE	620					
chr10	1683728	1683743	+	1	TARE	276					
chr10	1683863	1683878	+	1	TARE	120					
chr10	1683998	1684013	+	1	TARE	120					
chr10	1684289	1684304	+	1	TARE	276					
chr10	1684424	1684439	+	1	TARE	120					
chr10	1684559	1684574	+	1	TARE	120					
chr10	1684694	1684709		1	TARE	120					
chr10	1684829	1684844		1	TARE	120					
chr10	1684964	1684979		1	TARE	120					
chr11	2559	2574		-1	TARE						
chr11	2694	2709		-1	TARE	120					
chr11	2964	2979	+	-1	TARE	255					
chr11	3099	3114	+	-1	TARE	120					
chr11	3234	3249	+	-1	TARE	120					
chr11	3660	3675	+	-1	TARE	411					
chr11	3795	3810	+	-1	TARE	120					
chr11	3930	3945	+	-1	TARE	120					
chr11	4065	4080	+	-1	TARE	120					
chr11	4221	4236	+	-1	TARE	141					
chr11	4855	4870	+	-1	TARE	619					
chr11	5011	5026	+	1	TARE	141					
chr11	5193	5208	+	-1	TARE	167					
chr11	5538	5553	+	1	TARE	330					
chr11	5668	5684	+	-1	TARE	115	5				
chr11	5946	5961	+	1	TARE	262					
chr11	6636	6651	+	1	TARE	675					
chr11	8016	8031		1	TARE	1365					
chr11	9173	9189		1	TARE	1142					
chr11	9635	9650		-1	TARE	446					
chr11	21536	21552	+	1	var		5	PF11_0007		1	24160
chr11	21538	21554	+	-1	var	-14	5	PF11_0007			
chr11	21900	21915	+	1	var	346		PF11_0007			
chr11	21928	21943	+	1	var	13		PF11_0007			
chr11	21955	21970	+	1	var	12		PF11_0007			
chr11	21983	21998	+	1	var	13		PF11_0007			
chr11	22006	22021	+	1	var	8		PF11_0007			
chr11	22034	22049	+	1	var	13		PF11_0007			
chr11	22058	22073	+	1	var	9		PF11_0007			
chr11	22084	22099	+	1	var	11		PF11_0007			

chr11	490720	490735		1	hyp			PF11_0528		1	492090
chr11	607322	607337		1	hyp			PF11_0168	intron	-1	613393
chr11	663498	663513		1	hyp			PF11_0179		1	664162
chr11	1606698	1606714		-1	hyp		5	PF11_0414	PF11_0415	1	
chr12	1879	1894		-1	TARE						
chr12	2014	2029	+	-1	TARE	120					
chr12	2149	2164	+	-1	TARE	120					
chr12	2284	2299	+	-1	TARE	120					
chr12	2419	2434	+	-1	TARE	120					
chr12	2554	2569	+	-1	TARE	120					
chr12	2845	2860	+	-1	TARE	276					
chr12	2980	2995	+	-1	TARE	120					
chr12	3115	3130	+	-1	TARE	120					
chr12	3541	3556	+	-1	TARE	411					
chr12	4176	4191	+	-1	TARE	620					
chr12	4332	4347	+	1	TARE	141					
chr12	4514	4529	+	-1	TARE	167					
chr12	4859	4874	+	1	TARE	330					
chr12	4989	5005	+	-1	TARE	115	5				
chr12	7335	7350		1	TARE	2330					
chr12	8316	8331		-1	TARE	966					
chr12	8955	8970		-1	TARE	624					
chr12	14401	14417	+	1	var		5	PFL0005w		1	16973
chr12	14403	14419	+	-1	var	-14	5	PFL0005w			
chr12	14727	14742	+	1	var	308		PFL0005w			
chr12	14755	14770	+	1	var	13		PFL0005w			
chr12	14783	14798	+	1	var	13		PFL0005w			
chr12	14810	14825	+	1	var	12		PFL0005w			
chr12	14838	14853	+	1	var	13		PFL0005w			
chr12	14866	14881	+	1	var	13		PFL0005w			
chr12	205902	205917		-1	hyp			PFL0210c	exon	-1	205973
chr12	347208	347224		1	hyp		5	PFL0370w	exon 2	1	345917
chr12	776402	776417		-1	var			PFL0935c	central	-1	774190
chr12	776456	776471		-1	var	39		PFL0935c	central		
chr12	855439	855454		-1	hyp			PFL1025c		-1	854732
chr12	917547	917563		1	hyp		5	PFL1090w		1	918714
chr12	2123397	2123412		1	chitinase			PFL2510w		1	2125421
chr12	2251020	2251035	+	-1	var			PFL2665c		-1	2248945
chr12	2251047	2251062	+	-1	var	12		PFL2665c			
chr12	2251074	2251089	+	-1	var	12		PFL2665c			
chr12	2251101	2251116	+	-1	var	12		PFL2665c			
chr12	2251128	2251143	+	-1	var	12		PFL2665c			
chr12	2251155	2251170	+	-1	var	12		PFL2665c			
chr12	2251182	2251197	+	-1	var	12		PFL2665c			
chr12	2251209	2251224	+	-1	var	12		PFL2665c			
chr12	2251236	2251251	+	-1	var	12		PFL2665c			
chr12	2251263	2251278	+	-1	var	12		PFL2665c			
chr12	2251550	2251565	+	1	var	272		PFL2665c			
chr12	2251552	2251567	+	-1	var	-13		PFL2665c			
chr12	2251604	2251620	+	1	var	37	5	PFL2665c			
chr12	2251606	2251622	+	-1	var	-14	5	PFL2665c			
chr12	2251704	2251720	+	1	var	82	5	PFL2665c			
chr12	2251706	2251722	+	-1	var	-14	5	PFL2665c			
chr12	2262775	2262790		1	TARE						
chr12	2264385	2264400		-1	TARE	1595					
chr12	2266730	2266746	+	1	TARE	2330	5				
chr12	2266861	2266876	+	-1	TARE	115					
chr12	2267206	2267221	+	1	TARE	330					
chr12	2267388	2267403	+	-1	TARE	167					
chr12	2267544	2267559		1	TARE	141					
chr12	2268177	2268192		1	TARE	618					
chr12	2268468	2268483	+	1	TARE	276					
chr12	2268603	2268618	+	1	TARE	120					
chr12	2268738	2268753	+	1	TARE	120					
chr12	2268873	2268888	+	1	TARE	120					
chr12	2269164	2269179	+	1	TARE	276					
chr12	2269299	2269314	+	1	TARE	120					
chr12	2269434	2269449	+	1	TARE	120					
chr12	2269569	2269584	+	1	TARE	120					
chr12	2269704	2269719		1	TARE	120					
chr12	2269839	2269854		1	TARE	120					
chr13	2605	2620		-1	TARE						
chr13	2740	2755		-1	TARE	120					
chr13	3010	3025		-1	TARE	255					
chr13	3145	3160		-1	TARE	120					
chr13	3280	3295		-1	TARE	120					
chr13	3571	3586		-1	TARE	276					
chr13	3706	3721		-1	TARE	120					
chr13	3841	3856		-1	TARE	120					
chr13	3976	3991		-1	TARE	120					
chr13	4111	4126		-1	TARE	120					
chr13	4267	4282		-1	TARE	141					
chr13	4906	4921		-1	TARE	624					
chr13	5062	5077		1	TARE	141					

chr13	5244	5259		-1	TARE	167					
chr13	5589	5604		1	TARE	330					
chr13	5719	5735		-1	TARE	115	5				
chr13	5743	5758		-1	TARE	8					
chr13	8066	8081		1	TARE	2308					
chr13	9685	9700		-1	TARE	1604					
chr13	18709	18725		1	var		5	MAL13P1.1		1	21467
chr13	18711	18727		-1	var	-14	5	MAL13P1.1			
chr13	19004	19019		1	var	277		MAL13P1.1			
chr13	19032	19047		1	var	13		MAL13P1.1			
chr13	19060	19075		1	var	13		MAL13P1.1			
chr13	19088	19103		1	var	13		MAL13P1.1			
chr13	19116	19131		1	var	13		MAL13P1.1			
chr13	19144	19159		1	var	13		MAL13P1.1			
chr13	19172	19187		1	var	13		MAL13P1.1			
chr13	19200	19215		1	var	13		MAL13P1.1			
chr13	19228	19243		1	var	13		MAL13P1.1			
chr13	19256	19271		1	var	13		MAL13P1.1			
chr13	19284	19299		1	var	13		MAL13P1.1			
chr13	19312	19327		1	var	13		MAL13P1.1			
chr13	19340	19355		1	var	13		MAL13P1.1			
chr13	19367	19382		1	var	12		MAL13P1.1			
chr13	981644	981660		-1	hyp		5	PF13_0134	MAL13P1.13(	1	
chr13	1331766	1331781		1	hyp			PF13_0173		1	1332497
chr13	2866730	2866745	+	-1	var			MAL13P1.356		-1	2864550
chr13	2866752	2866767	+	-1	var	7		MAL13P1.356			
chr13	2866774	2866789	+	-1	var	7		MAL13P1.356			
chr13	2866796	2866811	+	-1	var	7		MAL13P1.356			
chr13	2866818	2866833	+	-1	var	7		MAL13P1.356			
chr13	2866840	2866855	+	-1	var	7		MAL13P1.356			
chr13	2866862	2866877	+	-1	var	7		MAL13P1.356			
chr13	2866884	2866899	+	-1	var	7		MAL13P1.356			
chr13	2866906	2866921	+	-1	var	7		MAL13P1.356			
chr13	2866928	2866943	+	-1	var	7		MAL13P1.356			
chr13	2866950	2866965	+	-1	var	7		MAL13P1.356			
chr13	2866972	2866987	+	-1	var	7		MAL13P1.356			
chr13	2866994	2867009	+	-1	var	7		MAL13P1.356			
chr13	2867212	2867228	+	1	var	203	5	MAL13P1.356			
chr13	2867214	2867230	+	-1	var	-14	5	MAL13P1.356			
chr13	2884497	2884512		1	TARE						
chr13	2886105	2886120		-1	TARE	1593					
chr13	2887760	2887776	+	1	TARE	1640	5				
chr13	2888173	2888188	+	-1	TARE	397					
chr13	2888450	2888466	+	1	TARE	262	5				
chr13	2888581	2888596	+	-1	TARE	115					
chr13	2888926	2888941		1	TARE	330					
chr13	2889108	2889123	+	-1	TARE	167					
chr13	2889264	2889279	+	1	TARE	141					
chr13	2889840	2889855	+	1	TARE	561					
chr13	2890266	2890281	+	1	TARE	411					
chr13	2890401	2890416	+	1	TARE	120					
chr13	2890536	2890551	+	1	TARE	120					
chr13	2890827	2890842	+	1	TARE	276					
chr13	2890962	2890977	+	1	TARE	120					
chr13	2891097	2891112	+	1	TARE	120					
chr13	2891367	2891382		1	TARE	255					
chr13	2891502	2891517		1	TARE	120					
chr14	657954	657969		1	hyp			PF14_0161		1	659538
chr14	1237682	1237698		-1	hyp		5	PF14_0293		-1	1237067
chr14	1919382	1919397		-1	putative centrin			PF14_0443		-1	1917953
chr14	2261182	2261198		-1				PF14_0527	exon	1	2259401
chr2	3053	3068	+	-1	TARE						
chr2	3188	3203	+	-1	TARE	120					
chr2	3323	3338	+	-1	TARE	120					
chr2	3458	3473	+	-1	TARE	120					
chr2	3593	3608	+	-1	TARE	120					
chr2	4019	4034	+	-1	TARE	411					
chr2	4154	4169	+	-1	TARE	120					
chr2	4289	4304	+	-1	TARE	120					
chr2	4559	4574	+	-1	TARE	255					
chr2	4715	4730	+	-1	TARE	141					
chr2	5350	5365	+	-1	TARE	620					
chr2	5506	5521	+	1	TARE	141					
chr2	5688	5703	+	-1	TARE	167					
chr2	6033	6048	+	1	TARE	330					
chr2	6163	6179	+	-1	TARE	115	5				
chr2	6441	6456	+	1	TARE	262					
chr2	6852	6868	+	-1	TARE	396	5				
chr2	7130	7145	+	1	TARE	262					
chr2	8509	8524	+	1	TARE	1364					
chr2	10100	10115		-1	TARE	1576					
chr2	22417	22433	+	1	var		5	PFB0010w		1	25232
chr2	22419	22435	+	-1	var	-14	5	PFB0010w			
chr2	22475	22491	+	1	var	40	5	PFB0010w			

chr2	22477	22493	+	-1	var	-14	5	PFB0010w			
chr2	22910	22925	+	1	var	417		PFB0010w			
chr2	22937	22952	+	1	var	12		PFB0010w			
chr2	22964	22979	+	1	var	12		PFB0010w			
chr2	22992	23007	+	1	var	13		PFB0010w			
chr2	23020	23035	+	1	var	13		PFB0010w			
chr2	23047	23062	+	1	var	12		PFB0010w			
chr2	23074	23089		1	var	12		PFB0010w			
chr2	23102	23117		1	var	13		PFB0010w			
chr2	610088	610104		-1	hyp		5	PFB0680w	exon1	1	610087
chr2	925761	925776		-1	var			PFB1055c		-1	923648
chr2	925783	925798		-1	var	7		PFB1055c			
chr2	925805	925820		-1	var	7		PFB1055c			
chr2	925827	925842		-1	var	7		PFB1055c			
chr2	925849	925864		-1	var	7		PFB1055c			
chr2	925871	925886		-1	var	7		PFB1055c			
chr2	925893	925908		-1	var	7		PFB1055c			
chr2	925915	925930		-1	var	7		PFB1055c			
chr2	925937	925952		-1	var	7		PFB1055c			
chr2	925959	925974		-1	var	7		PFB1055c			
chr2	925981	925996		-1	var	7		PFB1055c			
chr2	926003	926018		-1	var	7		PFB1055c			
chr2	926025	926040		-1	var	7		PFB1055c			
chr2	926052	926067		-1	var	12		PFB1055c			
chr2	926282	926298	+	1	var	215	5	PFB1055c			
chr2	926284	926300	+	-1	var	-14	5	PFB1055c			
chr2	926385	926401	+	1	var	85	5	PFB1055c			
chr2	926387	926403	+	-1	var	-14	5	PFB1055c			
chr2	937952	937967		1	TARE						
chr2	939552	939567		-1	TARE	1585					
chr2	941897	941913	+	1	TARE	2330	5				
chr2	942028	942043	+	-1	TARE	115					
chr2	942555	942570		-1	TARE	512					
chr2	942711	942726		1	TARE	141					
chr2	943348	943363		1	TARE	622					
chr2	943639	943654		1	TARE	276					
chr2	943774	943789	+	1	TARE	120					
chr2	943909	943924	+	1	TARE	120					
chr2	944044	944059	+	1	TARE	120					
chr2	944335	944350	+	1	TARE	276					
chr2	944605	944620	+	1	TARE	255					
chr2	944875	944890	+	1	TARE	255					
chr2	945010	945025	+	1	TARE	120					
chr2	945012	945027	+	-1	TARE	-13					
chr3	1362	1377		-1	TARE						
chr3	1497	1512		-1	TARE	120					
chr3	1767	1782	+	-1	TARE	255					
chr3	1902	1917	+	-1	TARE	120					
chr3	2037	2052	+	-1	TARE	120					
chr3	2328	2343	+	-1	TARE	276					
chr3	2463	2478	+	-1	TARE	120					
chr3	2598	2613	+	-1	TARE	120					
chr3	3024	3039	+	-1	TARE	411					
chr3	3743	3758	+	-1	TARE	704					
chr3	3899	3914	+	1	TARE	141					
chr3	4081	4096	+	-1	TARE	167					
chr3	4426	4441	+	1	TARE	330					
chr3	4556	4572	+	-1	TARE	115	5				
chr3	6903	6918		1	TARE	2331					
chr3	8491	8506		-1	TARE	1573					
chr3	30989	31005	+	1	var		5	PFC0005w		1	33641
chr3	30991	31007	+	-1	var	-14	5	PFC0005w			
chr3	31320	31335	+	1	var	313		PFC0005w			
chr3	31343	31358	+	1	var	8		PFC0005w			
chr3	31365	31380	+	1	var	7		PFC0005w			
chr3	31387	31402	+	1	var	7		PFC0005w			
chr3	31415	31430	+	1	var	13		PFC0005w			
chr3	31442	31457	+	1	var	12		PFC0005w			
chr3	31470	31485	+	1	var	13		PFC0005w			
chr3	31498	31513	+	1	var	13		PFC0005w			
chr3	199711	199726		-1	hyp			PFC0180c	PFC0185w		
chr3	1037163	1037178		-1	var/hyp			PFC1120c		-1	1034924
chr3	1037190	1037205		-1	var/hyp	12		PFC1120c			
chr3	1037217	1037232		-1	var/hyp	12		PFC1120c			
chr3	1037244	1037259		-1	var/hyp	12		PFC1120c			
chr3	1037271	1037286		-1	var/hyp	12		PFC1120c			
chr3	1037299	1037314		-1	var/hyp	13		PFC1120c			
chr3	1037327	1037342		-1	var/hyp	13		PFC1120c			
chr3	1037355	1037370		-1	var/hyp	13		PFC1120c			
chr3	1037383	1037398		-1	var/hyp	13		PFC1120c			
chr3	1037410	1037425		-1	var/hyp	12		PFC1120c			
chr3	1037438	1037453		-1	var/hyp	13		PFC1120c			
chr3	1037465	1037480		-1	var/hyp	12		PFC1120c			
chr3	1037492	1037507		-1	var/hyp	12		PFC1120c			

chr3	1037520	1037535		-1	var/hyp	13		PFC1120c			
chr3	1037542	1037557		-1	var/hyp	7		PFC1120c			
chr3	1037566	1037581		-1	var/hyp	9		PFC1120c			
chr3	1037588	1037603		-1	var/hyp	7		PFC1120c			
chr3	1037610	1037625		-1	var/hyp	7		PFC1120c			
chr3	1037840	1037856	+	1	var/hyp	215		PFC1120c			
chr3	1037842	1037858	+	-1	var/hyp	-14		PFC1120c			
chr3	1038022	1038038	+	1	var/hyp	164		PFC1120c			
chr3	1038024	1038040	+	-1	var/hyp	-14		PFC1120c			
chr3	1051232	1051247		1	TARE						
chr3	1051871	1051886		1	TARE	624					
chr3	1052851	1052866		-1	TARE	965					
chr3	1054920	1054935	+	-1	TARE	2054					
chr3	1055197	1055213	+	1	TARE	262	5				
chr3	1055328	1055343	+	-1	TARE	115					
chr3	1055673	1055688	+	1	TARE	330					
chr3	1055855	1055870	+	-1	TARE	167					
chr3	1056011	1056026	+	1	TARE	141					
chr3	1056646	1056661	+	1	TARE	620					
chr3	1057072	1057087	+	1	TARE	411					
chr3	1057207	1057222	+	1	TARE	120					
chr3	1057342	1057357	+	1	TARE	120					
chr3	1057633	1057648	+	1	TARE	276					
chr3	1057768	1057783	+	1	TARE	120					
chr3	1057903	1057918	+	1	TARE	120					
chr3	1058173	1058188	+	1	TARE	255					
chr3	1058308	1058323		1	TARE	120					
chr4	8314	8329	+	-1	TARE						
chr4	8449	8464	+	-1	TARE	120					
chr4	8719	8734	+	-1	TARE	255					
chr4	8854	8869	+	-1	TARE	120					
chr4	8989	9004	+	-1	TARE	120					
chr4	9281	9296	+	-1	TARE	277					
chr4	9416	9431	+	-1	TARE	120					
chr4	9551	9566	+	-1	TARE	120					
chr4	9842	9857	+	-1	TARE	276					
chr4	10478	10493	+	-1	TARE	621					
chr4	10634	10649	+	1	TARE	141					
chr4	10816	10831	+	-1	TARE	167					
chr4	11161	11176	+	1	TARE	330					
chr4	11291	11307	+	-1	TARE	115	5				
chr4	11570	11585	+	1	TARE	263					
chr4	13638	13653		1	TARE	2053					
chr4	15257	15272		-1	TARE	1604					
chr4	32590	32606	+	1	var		5	PFD0005w		1	35153
chr4	32592	32608	+	-1	var	-14	5	PFD0005w			
chr4	32690	32706	+	1	var	82	5	PFD0005w			
chr4	32692	32708	+	-1	var	-14	5	PFD0005w			
chr4	32864	32879	+	1	var	156		PFD0005w			
chr4	32887	32902	+	1	var	8		PFD0005w			
chr4	32910	32925	+	1	var	8		PFD0005w			
chr4	32937	32952	+	1	var	12		PFD0005w			
chr4	32964	32979	+	1	var	12		PFD0005w			
chr4	32991	33006	+	1	var	12		PFD0005w			
chr4	270350	270365		1	protease putative			PFD0230c	PFD0235c	-1	
chr4	270352	270367		-1	protease putative	-13		PFD0230c		-1	269883
chr4	326137	326153		1	hyp		5	PFD0300w		1	327491
chr4	879424	879439		1	hyp			PFD0955w		1	880118
chr4	1185858	1185873		-1	var			PFD1245c		-1	1183848
chr4	1185885	1185900		-1	var	12		PFD1245c			
chr4	1185912	1185927		-1	var	12		PFD1245c			
chr4	1185939	1185954		-1	var	12		PFD1245c			
chr4	1185966	1185981		-1	var	12		PFD1245c			
chr4	1185993	1186008		-1	var	12		PFD1245c			
chr4	1186020	1186035		-1	var	12		PFD1245c			
chr4	1186047	1186062		-1	var	12		PFD1245c			
chr4	1186075	1186090		-1	var	13		PFD1245c			
chr4	1186102	1186117		-1	var	12		PFD1245c			
chr4	1186130	1186145		-1	var	13		PFD1245c			
chr4	1186158	1186173		-1	var	13		PFD1245c			
chr4	1186181	1186196		-1	var	8		PFD1245c			
chr4	1186563	1186579	+	1	var	367	5	PFD1245c			
chr4	1186565	1186581		-1	var	-14	5	PFD1245c			
chr4	1195676	1195691		1	hyp	9095		PFD1250w	intron	1	1192754
chr4	1197297	1197312		-1	TARE	1606					
chr4	1198952	1198968	+	1	TARE	1640	5				
chr4	1199083	1199098	+	-1	TARE	115					
chr4	1199428	1199443	+	1	TARE	330					
chr4	1199610	1199625	+	-1	TARE	167					
chr4	1199766	1199781		1	TARE	141					
chr4	1200401	1200416		1	TARE	620					
chr4	1200557	1200572		1	TARE	141					
chr4	1200692	1200707		1	TARE	120					
chr4	1200827	1200842		1	TARE	120					

chr4	1200962	1200977		1	TARE	120					
chr4	1201253	1201268		1	TARE	276					
chr4	1201388	1201403	+	1	TARE	120					
chr4	1201523	1201538	+	1	TARE	120					
chr4	1201793	1201808		1	TARE	255					
chr4	1201928	1201943		1	TARE	120					
chr5	2067	2082		-1	TARE						
chr5	2202	2217		-1	TARE	120					
chr5	2472	2487		-1	TARE	255					
chr5	2607	2622		-1	TARE	120					
chr5	2742	2757		-1	TARE	120					
chr5	3033	3048		-1	TARE	276					
chr5	3168	3183		-1	TARE	120					
chr5	3303	3318		-1	TARE	120					
chr5	3438	3453		-1	TARE	120					
chr5	3573	3588		-1	TARE	120					
chr5	3729	3744		-1	TARE	141					
chr5	4368	4383		-1	TARE	624					
chr5	4524	4539		1	TARE	141					
chr5	4706	4721		-1	TARE	167					
chr5	5051	5066		1	TARE	330					
chr5	5181	5197		-1	TARE	115	5				
chr5	5205	5220		-1	TARE	8					
chr5	7528	7543		1	TARE	2308					
chr5	9147	9162		-1	TARE	1604					
chr5	18171	18187		1	var		5	PFE0005w		1	20929
chr5	18173	18189		-1	var	-14	5	PFE0005w			
chr5	18466	18481		1	var	277		PFE0005w			
chr5	18494	18509		1	var	13		PFE0005w			
chr5	18522	18537		1	var	13		PFE0005w			
chr5	18550	18565		1	var	13		PFE0005w			
chr5	18578	18593		1	var	13		PFE0005w			
chr5	18606	18621		1	var	13		PFE0005w			
chr5	18634	18649		1	var	13		PFE0005w			
chr5	18662	18677		1	var	13		PFE0005w			
chr5	18690	18705		1	var	13		PFE0005w			
chr5	18718	18733		1	var	13		PFE0005w			
chr5	18746	18761		1	var	13		PFE0005w			
chr5	18774	18789		1	var	13		PFE0005w			
chr5	18802	18817		1	var	13		PFE0005w			
chr5	18829	18844		1	var	12		PFE0005w			
chr5	82925	82941		-1	rap3		5	PFE0075c		-1	82336
chr5	85954	85970		-1	rap2		5	PFE0080c		-1	85237
chr5	365711	365726		1	hyp			PFE0440w		1	367022
chr5	898616	898631		1	hyp			PFE1105c	exon	-1	899751
chr6	545545	545561		1	ribosomal protein L18, putative		5	PFF0645c		1	546959
chr6	880216	880232		1	hyp		5	PFF1040w		1	881961
chr6	1384746	1384761	+	-1	var			PFF1595c		-1	1382628
chr6	1384773	1384788	+	-1	var	12		PFF1595c			
chr6	1384800	1384815	+	-1	var	12		PFF1595c			
chr6	1384827	1384842	+	-1	var	12		PFF1595c			
chr6	1385040	1385056	+	-1	var	198	5	PFF1595c			
chr6	1406100	1406115		1	TARE						
chr6	1406728	1406743		1	TARE	613					
chr6	1407708	1407723		-1	TARE	965					
chr6	1411844	1411859	+	-1	TARE	4121					
chr6	1412121	1412137		1	TARE	262	5				
chr6	1412252	1412267		-1	TARE	115					
chr6	1412597	1412612		1	TARE	330					
chr6	1412779	1412794		-1	TARE	167					
chr6	1412935	1412950		1	TARE	141					
chr6	1413571	1413586		1	TARE	621					
chr6	1413862	1413877		1	TARE	276					
chr6	1413997	1414012		1	TARE	120					
chr6	1414132	1414147		1	TARE	120					
chr6	1414424	1414439		1	TARE	277					
chr6	1414559	1414574		1	TARE	120					
chr6	1414694	1414709		1	TARE	120					
chr6	1414964	1414979		1	TARE	255					
chr6	1415099	1415114		1	TARE	120					
chr7	3689	3704		-1	TARE						
chr7	3824	3839		-1	TARE	120					
chr7	4094	4109		-1	TARE	255					
chr7	4229	4244		-1	TARE	120					
chr7	4364	4379		-1	TARE	120					
chr7	4655	4670		-1	TARE	276					
chr7	4790	4805		-1	TARE	120					
chr7	4925	4940		-1	TARE	120					
chr7	5060	5075		-1	TARE	120					
chr7	5195	5210		-1	TARE	120					
chr7	5330	5345		-1	TARE	120					
chr7	5465	5480		-1	TARE	120					
chr7	5600	5615		-1	TARE	120					
chr7	5735	5750		-1	TARE	120					

chr7	5870	5885		-1	TARE	120					
chr7	6005	6020		-1	TARE	120					
chr7	6140	6155		-1	TARE	120					
chr7	6275	6290		-1	TARE	120					
chr7	6410	6425		-1	TARE	120					
chr7	6566	6581		-1	TARE	141					
chr7	7138	7153		-1	TARE	557					
chr7	7294	7309		1	TARE	141					
chr7	7476	7491		-1	TARE	167					
chr7	7821	7836		1	TARE	330					
chr7	7951	7967		-1	TARE	115	5				
chr7	9608	9623		1	TARE	1641					
chr7	10580	10595		-1	TARE	957					
chr7	11208	11223		-1	TARE	613					
chr7	27640	27656		1	var		5	MAL7P1.212		1	30673
chr7	27642	27658		-1	var	-14	5	MAL7P1.212			
chr7	27695	27711		1	var	37	5	MAL7P1.212			
chr7	27697	27713		-1	var	-14	5	MAL7P1.212			
chr7	28211	28226		1	var	498		MAL7P1.212			
chr7	28238	28253		1	var	12		MAL7P1.212			
chr7	28265	28280		1	var	12		MAL7P1.212			
chr7	28294	28309		1	var	14		MAL7P1.212			
chr7	28322	28337		1	var	13		MAL7P1.212			
chr7	28350	28365		1	var	13		MAL7P1.212			
chr7	28378	28393		1	var	13		MAL7P1.212			
chr7	28406	28421		1	var	13		MAL7P1.212			
chr7	28434	28449		1	var	13		MAL7P1.212			
chr7	28462	28477		1	var	13		MAL7P1.212			
chr7	28486	28501		1	var	9		MAL7P1.212			
chr7	28510	28525		1	var	9		MAL7P1.212			
chr7	28538	28553		1	var	13		MAL7P1.212			
chr7	1483602	1483617		-1	var			MAL7P1.187		-1	1481610
chr7	1483630	1483645		-1	var	13		MAL7P1.187			
chr7	1483657	1483672		-1	var	12		MAL7P1.187			
chr7	1483685	1483700		-1	var	13		MAL7P1.187			
chr7	1483708	1483723		-1	var	8		MAL7P1.187			
chr7	1483735	1483750		-1	var	12		MAL7P1.187			
chr7	1484407	1484423	+	1	var	657	5	MAL7P1.187			
chr7	1484409	1484425	+	-1	var	-14	5	MAL7P1.187			
chr7	1492987	1493002		1	TARE						
chr7	1494589	1494604		-1	TARE	1587					
chr7	1496657	1496672	+	-1	TARE	2053					
chr7	1496934	1496950	+	1	TARE	262	5				
chr7	1497065	1497080	+	-1	TARE	115					
chr7	1497410	1497425	+	1	TARE	330					
chr7	1497592	1497607		-1	TARE	167					
chr7	1498425	1498440		1	TARE	818					
chr7	1498716	1498731	+	1	TARE	276					
chr7	1498851	1498866	+	1	TARE	120					
chr7	1498986	1499001	+	1	TARE	120					
chr7	1499277	1499292	+	1	TARE	276					
chr7	1499412	1499427	+	1	TARE	120					
chr7	1499547	1499562	+	1	TARE	120					
chr7	1499817	1499832	+	1	TARE	255					
chr7	1499952	1499967		1	TARE	120					
chr8	3090	3105		-1	TARE						
chr8	3225	3240		-1	TARE	120					
chr8	3360	3375	+	-1	TARE	120					
chr8	3495	3510	+	-1	TARE	120					
chr8	3630	3645	+	-1	TARE	120					
chr8	3765	3780	+	-1	TARE	120					
chr8	4056	4071	+	-1	TARE	276					
chr8	4191	4206	+	-1	TARE	120					
chr8	4326	4341	+	-1	TARE	120					
chr8	4461	4476	+	-1	TARE	120					
chr8	4752	4767		-1	TARE	276					
chr8	5387	5402		-1	TARE	620					
chr8	5543	5558		1	TARE	141					
chr8	5725	5740	+	-1	TARE	167					
chr8	6070	6085	+	1	TARE	330					
chr8	6200	6216	+	-1	TARE	115	5				
chr8	6478	6493	+	1	TARE	262					
chr8	6914	6929	+	-1	TARE	421					
chr8	7168	7183	+	1	TARE	239					
chr8	7857	7872	+	1	TARE	674					
chr8	19536	19552	+	1	var		5	PF08_0142		1	22369
chr8	19538	19554	+	-1	var	-14	5	PF08_0142			
chr8	19915	19930	+	1	var	361		PF08_0142			
chr8	19938	19953	+	1	var	8		PF08_0142			
chr8	19961	19976	+	1	var	8		PF08_0142			
chr8	19984	19999	+	1	var	8		PF08_0142			
chr8	20007	20022	+	1	var	8		PF08_0142			
chr8	20030	20045	+	1	var	8		PF08_0142			
chr8	20053	20068	+	1	var	8		PF08_0142			

chr8	20075	20090	+	1	var	7		PF08_0142			
chr8	20097	20112	+	1	var	7		PF08_0142			
chr8	20119	20134	+	1	var	7		PF08_0142			
chr8	20147	20162	+	1	var	13		PF08_0142			
chr8	20174	20189	+	1	var	12		PF08_0142			
chr8	20202	20217	+	1	var	13		PF08_0142			
chr8	20229	20244	+	1	var	12		PF08_0142			
chr8	20256	20271	+	1	var	12		PF08_0142			
chr8	26307	26323		1	var		5	PF08_0142	exon 1		
chr8	208975	208991		1	hyp		5	MAL8P1.145		1	209571
chr8	400130	400145		-1	serine protease putative			MAL8P1.126		-1	399909
chr8	1124705	1124721		-1	ubiquitin protein ligase 1, putative		5	MAL8P1.23	exon	-1	1140216
chr8	1401461	1401476	+	-1	var			MAL8P1.220		-1	1399234
chr8	1401488	1401503	+	-1	var	12		MAL8P1.220			
chr8	1401515	1401530	+	-1	var	12		MAL8P1.220			
chr8	1401542	1401557	+	-1	var	12		MAL8P1.220			
chr8	1401570	1401585	+	-1	var	13		MAL8P1.220			
chr8	1401598	1401613	+	-1	var	13		MAL8P1.220			
chr8	1401625	1401640	+	-1	var	12		MAL8P1.220			
chr8	1401653	1401668	+	-1	var	13		MAL8P1.220			
chr8	1401680	1401695	+	-1	var	12		MAL8P1.220			
chr8	1401707	1401722	+	-1	var	12		MAL8P1.220			
chr8	1401730	1401745	+	-1	var	8		MAL8P1.220			
chr8	1402058	1402074	+	1	var	313	5	MAL8P1.220			
chr8	1402060	1402076	+	-1	var	-14	5	MAL8P1.220			
chr8	1410598	1410613		1	hyp	8522		MAL8P1.330		1	1412780
chr8	1411226	1411241		1	hyp	613		MAL8P1.330		1	1412780
chr8	1412206	1412221	+	-1	hyp	965		MAL8P1.330		1	1412780
chr8	1413861	1413877	+	1	TARE	1640	5				
chr8	1413992	1414007	+	-1	TARE	115					
chr8	1414337	1414352	+	1	TARE	330					
chr8	1414519	1414534	+	-1	TARE	167					
chr8	1414675	1414690	+	1	TARE	141					
chr8	1415310	1415325	+	1	TARE	620					
chr8	1415466	1415481	+	1	TARE	141					
chr8	1415601	1415616	+	1	TARE	120					
chr8	1415736	1415751	+	1	TARE	120					
chr8	1415871	1415886	+	1	TARE	120					
chr8	1416006	1416021	+	1	TARE	120					
chr8	1416297	1416312	+	1	TARE	276					
chr8	1416432	1416447	+	1	TARE	120					
chr8	1416567	1416582	+	1	TARE	120					
chr8	1417242	1417257		1	TARE	660					
chr8	1417377	1417392		1	TARE	120					
chr9	979	994	+	-1	TARE						
chr9	1114	1129	+	-1	TARE	120					
chr9	1249	1264	+	-1	TARE	120					
chr9	1384	1399	+	-1	TARE	120					
chr9	1519	1534	+	-1	TARE	120					
chr9	1654	1669	+	-1	TARE	120					
chr9	1789	1804	+	-1	TARE	120					
chr9	2080	2095	+	-1	TARE	276					
chr9	2215	2230	+	-1	TARE	120					
chr9	2350	2365	+	-1	TARE	120					
chr9	2641	2656	+	-1	TARE	276					
chr9	3277	3292	+	-1	TARE	621					
chr9	3433	3448	+	1	TARE	141					
chr9	3615	3630	+	-1	TARE	167					
chr9	3960	3975	+	1	TARE	330					
chr9	4090	4106	+	-1	TARE	115	5				
chr9	6436	6451		1	TARE	2330					
chr9	8045	8060		-1	TARE	1594					
chr9	17198	17214	+	1	var		5	PFI0005w		1	20080
chr9	17200	17216	+	-1	var	-14	5	PFI0005w			
chr9	17255	17271	+	1	var	39	5	PFI0005w			
chr9	17257	17273	+	-1	var	-14	5	PFI0005w			
chr9	17527	17542	+	1	var	254		PFI0005w			
chr9	17554	17569	+	1	var	12		PFI0005w			
chr9	17581	17596	+	1	var	12		PFI0005w			
chr9	17608	17623	+	1	var	12		PFI0005w			
chr9	17635	17650	+	1	var	12		PFI0005w			
chr9	17662	17677	+	1	var	12		PFI0005w			
chr9	17689	17704	+	1	var	12		PFI0005w			
chr9	17716	17731	+	1	var	12		PFI0005w			
chr9	17743	17758	+	1	var	12		PFI0005w			
chr9	17771	17786	+	1	var	13		PFI0005w			
chr9	17798	17813	+	1	var	12		PFI0005w			
chr9	17826	17841	+	1	var	13		PFI0005w			
chr9	17853	17868	+	1	var	12		PFI0005w			
chr9	17881	17896	+	1	var	13		PFI0005w			
chr9	17908	17923	+	1	var	12		PFI0005w			
chr9	275999	276014		-1	RHOPH3			PFI0265c		-1	274787
chr9	373429	373444		-1	S1/P1 nuclease, putative			PFI0385c		-1	370444
chr9	398159	398174		-1	hyp			PFI0410c		-1	398007

chr9	616555	616571		1	hyp		5	PFI0705w		1	617954
chr9	1359737	1359752		-1	hyp			PFI1680w	PFI1685w	1	
chr9	1505450	1505465		-1	var			PFI1830c		-1	1503324
chr9	1505477	1505492		-1	var	12		PFI1830c			
chr9	1505505	1505520		-1	var	13		PFI1830c			
chr9	1505532	1505547	+	-1	var	12		PFI1830c			
chr9	1505559	1505574	+	-1	var	12		PFI1830c			
chr9	1505587	1505602	+	-1	var	13		PFI1830c			
chr9	1505614	1505629	+	-1	var	12		PFI1830c			
chr9	1505641	1505656	+	-1	var	12		PFI1830c			
chr9	1505669	1505684	+	-1	var	13		PFI1830c			
chr9	1505692	1505707	+	-1	var	8		PFI1830c			
chr9	1505715	1505730	+	-1	var	8		PFI1830c			
chr9	1506187	1506202	+	1	var	457		PFI1830c			
chr9	1506189	1506204	+	-1	var	-13		PFI1830c			
chr9	1506243	1506259	+	1	var	39	5	PFI1830c			
chr9	1506245	1506261	+	-1	var	-14	5	PFI1830c			
chr9	1532044	1532059		1	TARE						
chr9	1533643	1533658		-1	TARE	1584					
chr9	1535711	1535726	+	-1	TARE	2053					
chr9	1535988	1536004	+	1	TARE	262	5				
chr9	1536119	1536134	+	-1	TARE	115					
chr9	1536464	1536479	+	1	TARE	330					
chr9	1536646	1536661	+	-1	TARE	167					
chr9	1536802	1536817	+	1	TARE	141					
chr9	1537441	1537456	+	1	TARE	624					
chr9	1537732	1537747	+	1	TARE	276					
chr9	1537867	1537882	+	1	TARE	120					
chr9	1538137	1538152	+	1	TARE	255					
chr9	1538428	1538443	+	1	TARE	276					
chr9	1538563	1538578	+	1	TARE	120					
chr9	1538698	1538713	+	1	TARE	120					
chr9	1538968	1538983	+	1	TARE	255					
chr9	1539103	1539118	+	1	TARE	120					
