# Supplementary material for: A Major Role for the Plasmodium falciparum ApiAP2 Protein PfSIP2 in Chromosome End Biology
Source: PLoS Pathog. 2010 Feb 26;6(2):e1000784. doi: 10.1371/journal.ppat.1000784 (PMC2829057; doi:10.1371/journal.ppat.1000784)
Supplement: Table S4 — Summary of the genome-wide location of SPE2 elements and their characteristics. Column 1: Classification of SPE2-associated loci into three groups (var genes, other genes, TARE). Column 2: The groups classified in column 1 are further subgrouped according to various criteria (see also Figure 3D). Column 3: total number of SPE2 sites in each subgroup. Column 4: number of SPE2-associated genes in each subgroup. Column 5: number of SPE2 sites in each subgroup that are located in either subtelomeric or chromosome-central positions. Column 6: number of SPE2 sites in each subgroup in either sense or antisense orientation with respect to the orientation of the SPE2-associated gene. Column 7: average distance between, and average number of, SPE2 sites in SPE2-defined subtelomeric landmarks. Column 8: number of SPE2 sites in each subgroup with either 4bp or 5bp spacing between half sites. Column 9: relative position of the most upstream (“first”) and downstream (“last”) SPE2 sites upstream of SPE2-associated genes. (0.01 MB PDF) [file ppat.1000784.s008.pdf]

Table S4: Summary genome-wide SPE2 prediction

1	2	3	4	5		6		7		8		9	
SPE2 classification	SPE2 type	total #	# of genes	chr. location		orientation		clustered SPE2 elements		spacing between half sites		rel. position to ATG	
				subtel.	cen.	sense	antisense	avg. distance	avg. # of SPE2	4 bp	5 bp	first	last
**var genes**	**upsB**	**330**	**24**	**328**	**2**	**297**	**33**			**268**	**62**		
	cluster 1	264	24	262	2	264	0	12.3 (+/- 5.9)	11.0 (+/- 5.7)	264	0	2132.4 (+/- 70.2)	2403.1 (+/- 172.2)
	cluster 2	66	22	66	0	33	33	62.1 (+/- 43.2)	3.0 (+/- 1.3)	4	62	2698.7 (+/- 162.3)	2774.3 (+/- 162.8)
**other genes**	**single SPE2**	**42**	**37**	**6**	**36**	**33**	**4**			**27**	**15**		
	upstream	27	27	3	24	27	0			18	9	1096.0 (+/- 646.8)	
	cds	10	10	2	8	6	4			6	4		
	intergenic	5		1	4					3	2		
	**multiple SPE2**	**3**	1	1	0	1	2			3	0	574	2182
**TARE**	**all**	**402**		**402**	**0**	**181**	**221**	**379.6 (+/- 525.2)**	**18.3 (+/- 2.5)**	**379**	**23**		
	left	232		232	0	47	185	365.1 (+/- 487.7)	19.3 (+/- 3.0)	219	13		
	right	170		170	0	134	36	399.5 (+/- 573.7)	17.0 (+/- 0.9)	160	10		
	**TOTAL**	**777**								**677**	**100**		
