# Supplementary material for: A Major Role for the Plasmodium falciparum ApiAP2 Protein PfSIP2 in Chromosome End Biology
Source: PLoS Pathog. 2010 Feb 26;6(2):e1000784. doi: 10.1371/journal.ppat.1000784 (PMC2829057; doi:10.1371/journal.ppat.1000784)
Supplement: Table S5 — Single SPE2 sites upstream of internal genes. Column 1: Chromosome ID. Column 2: nucleotide position of the first bp of the SPE2 motif with respect to the left telomere end. Column 3: nucleotide position of the last bp of the SPE2 motif with respect to the left telomere on each chromosome. Column 4: SPE2 orientation on the sense (“1”) or antisense (“−1”) strand. Column 5: PlasmoDB gene annotation of associated genes. Column 6: PlasmoDB accession number of associated genes. Column 7: Location of SPE2 element with respect to the coding sequence. Column 8: blank cells represent canonical 4bp spacing between the two half sites of the bipartite SPE2 motif. “5” represents 5bp spacing. Column 9: orientation of SPE2-associated genes on the sense (“1”) or antisense (“−1”) strand. Column 10: nucleotide position of the ATG start codon of SPE2-associated genes with respect to the left telomere on each chromosome. Column 11: distance of SPE2 element in bp upstream of associated genes. Column 12: Peak transcription. Values/information in individual columns was retrieved from PlasmoDB version 5.5 (www.plasmodb.org). (0.02 MB PDF) [file ppat.1000784.s009.pdf]

Table S5: Single SPE2 sites upstream of chromosome-internal genes

chromosome	SPE2 start	SPE2 end	+/-	annotation	accession	SPE2 location	5bp spacing	gene +/-	ATG	SPE2 upstream (bp)	Pf-iRBC+Spz+Gam max expr stage (Affy)
chr12	2123397	2123412	1	chitinase	PFL2510w	upstream		1	2125421	2024	Merozoite
chr12	917547	917563	1	glideosome-associated protein 45	PFL1090w	upstream	5	1	918714	1167	Late Schizogony
chr12	855439	855454	-1	conserved Plasmodium protein, unknown function	PFL1025c	upstream		-1	854732	722	Gametocyte
chr12	347208	347224	1	conserved Plasmodium protein, unknown function	PFL0370w	exon 2	5	1	345917		Gametocyte
chr12	205902	205917	-1	eukaryotic initiation factor 5a, putative	PFL0210c	exon		-1	205973		Early Trophozoite
chr9	1359737	1359752	-1	UBX domain, putative	PFI1680w	PFI1685w		1			Gametocyte
chr9	616555	616571	1	conserved Plasmodium protein, unknown function	PFI0705w	upstream	5	1	617954	1399	Gametocyte
chr9	398159	398174	-1	conserved Plasmodium protein, unknown function	PFI0410c	upstream		-1	398007	167	Late Schizogony
chr9	373429	373444	-1	P1 nuclease, putative	PFI0385c	upstream		-1	370444	3000	Late Schizogony
chr9	275999	276014	-1	RhopH3	PFI0265c	upstream		-1	274787	1227	Late Schizogony
chr6	880216	880232	1	conserved Plasmodium protein, unknown function	PFF1040w	upstream	5	1	881961	1745	Early Ring
chr6	545545	545561	1	integral membrane protein, putative	PFF0645c	upstream	5	1	546959	1414	Late Schizogony
chr5	898616	898631	1	conserved Plasmodium protein, unknown function	PFE1105c	exon		-1	899751		Early Schizogony
chr5	365711	365726	1	conserved Plasmodium protein, unknown function	PFE0440w	upstream		1	367022	1311	Late Schizogony
chr5	85954	85970	-1	rhoptry-associated protein 2, RAP2	PFE0080c	upstream	5	-1	85237	733	Early Schizogony
chr5	82925	82941	-1	rhoptry-associated protein 3, RAP3	PFE0075c	upstream	5	-1	82336	605	Late Schizogony
chr4	879424	879439	1	apical merozoite protein	PFD0955w	upstream		1	880118	694	Late Schizogony
chr4	326137	326153	1	conserved Plasmodium protein, unknown function	PFD0300w	upstream	5	1	327491	1354	Early Schizogony
chr4	270350	270365	1	protease, putative	PFD0230c	PFD0235c		-1			Late Schizogony
chr4	270352	270367	-1	protease, putative	PFD0230c	upstream		-1	269883	484	Late Schizogony
chr3	199711	199726	-1	membrane skeletal protein, putative	PFC0180c	PFC0185w					Merozoite
chr2	610088	610104	-1	rhoptry neck protein 6	PFB0680w	exon1	5	1	610087		Late Schizogony
chr14	2261182	2261198	-1	conserved Plasmodium protein, unknown function	PF14 0527	exon		1	2259401		Late Schizogony
chr14	1919382	1919397	-1	centrin-2	PF14 0443	upstream		-1	1917953	1444	Gametocyte
chr14	1237682	1237698	-1	conserved Plasmodium protein, unknown function	PF14 0293	upstream	5	-1	1237067	631	Late Schizogony
chr14	657954	657969	1	conserved protein, unknown function	PF14 0161	upstream		1	659538	1584	Merozoite
chr13	1331766	1331781	1	conserved Plasmodium protein, unknown function	PF13 0173	upstream		1	1332497	731	Late Schizogony
chr13	981644	981660	-1	conserved Plasmodium protein, unknown function	PF13 0134	MAL13P1.130	5	1			Gametocyte
chr11	490720	490735	1	conserved Plasmodium protein, unknown function	PF11 0528	upstream		1	492090	1370	Late Schizogony
chr11	1606698	1606714	-1	conserved Plasmodium protein, unknown function	PF11 0414	PF11 0415	5	1			Early Trophozoite
chr11	663498	663513	1	conserved Plasmodium protein, unknown function	PF11 0179	upstream		1	664162	664	Merozoite
chr11	607322	607337	1	moving junction protein	PF11 0168	intron		-1	613393		Late Schizogony
chr10	772648	772663	-1	conserved Plasmodium protein, unknown function	PF10 0184	upstream		-1	771953	710	Gametocyte
chr10	691351	691366	-1	conserved Plasmodium protein, unknown function	PF10 0166	upstream		-1	690819	547	Late Schizogony
chr10	609000	609015	1	FAD synthetase, putative	PF10 0147	upstream		1	611212	2212	Gametocyte
chr10	159324	159339	-1	conserved Plasmodium protein, unknown function	PF10 0037	upstream		-1	158517	822	Late Schizogony
chr8	1124705	1124721	-1	ubiquitin-protein ligase 1, putative	MAL8P1.23	exon	5	-1	1140216		Early Ring
chr8	208975	208991	1	mitogen-activated protein kinase organizer 1, putative	MAL8P1.145	upstream	5	1	209571	596	Gametocyte
chr8	400130	400145	-1	serine protease, putative	MAL8P1.126	upstream		-1	399909	236	Late Schizogony
chr4	1195676	1195691	1	hypothetical protein, conserved in P. falciparum	PFD1250w	intron		1	1192754		
chr8	1410598	1410613	1	hypothetical protein, conserved in P. falciparum	MAL8P1.330	upstream		-1	1412780	2182	
chr8	1411226	1411241	1	hypothetical protein, conserved in P. falciparum	MAL8P1.330	upstream		-1	1412780	1554	
chr8	1412206	1412221	-1	hypothetical protein, conserved in P. falciparum	MAL8P1.330	upstream		-1	1412780	574	
