# Supplementary material for: A Major Role for the Plasmodium falciparum ApiAP2 Protein PfSIP2 in Chromosome End Biology
Source: PLoS Pathog. 2010 Feb 26;6(2):e1000784. doi: 10.1371/journal.ppat.1000784 (PMC2829057; doi:10.1371/journal.ppat.1000784)
Supplement: Protocol S1 — Affinity purification and mass spectrometry-based identification of the SPE2-interacting protein PfSIP2. (0.03 MB DOC) [file ppat.1000784.s010.doc]

**Protocol S1**

**Affinity purification and mass spectrometry-based identification of the SPE2-interacting protein PfSIP2**

*Generation of the SPE2 affinity matrix*

Complementary and phosphorylated oligonucleotides SPE2concatF and SPE2concatR were annealed to produce SPE2concat (81bp) containing three consecutive SPE2 sites and 7b complementary 5' overhangs (sequence derived from the PFL0005w promoter). An adapter molecule (SPE2biotin) containing a single SPE2 motif and a 7b taaagtc 5' overhang (lower strand) was generated by annealing oligonucleotides SPE2biotinF (5' biotin) and SPE2biotinR (5' phosphate). 900 pmol SPE2concat were mixed with 300 pmol SPE2biotin and concatenated using T4 DNA ligase in 1ml at room temperature for 16 hrs. Successful concatenation was verified by agarose gel electrophoresis (size range: 82bp to > 3kb; average length: 500bp). After ethanol precipitation, 5' overhangs were polished with Klenow enzyme, precipitated and dissolved in 300 μl TE (approx. 200ng concatenated SPE2 elements/μl). 280 μl were bound to 5mg Dynabeads M280 (Dynal Biotech) in a 1ml volume for 30 min at room temperature. The supernatant was removed and beads washed twice with 1.5 ml and finally resuspended in 500μl 1M NaCl. Beads carrying immobilised mutated SPE2 elements were generated accordingly. Beads carrying single stranded competitor DNA were obtained by immobilising 3 nmol of a biotinylated 78 base oligonucleotide (SSCOMPbiotin) onto 5mg of beads as described above. See Protocol S4 for oligonucleotide sequences.

*Affinity purification of the SPE2-binding protein*

Nuclei were isolated from 5x10^10^ schizonts and nuclear proteins extracted with 7 ml 0.8M HSB (20mM HEPES (pH7.9), 1M KCl, 1mM EDTA, 1mM EGTA, 1mM DTT, protease inhibitors) by vortexing for 30min at 4°C. After centrifugation at 13'000 rpm for 30 min the supernatant was adjusted to approx. 400mM KCl by adding ¼ vol dilution buffer (20mM Hepes pH 7.9, 1mM EDTA, 1mM EGTA, 1mM DTT, 40% glycerol). 6ml nuclear extract were diluted fivefold to adjust for binding conditions (20mM Hepes pH 7.9, 100mM KCl, 1mM EDTA, 2mM DTT, 2mM MgCl_2_, 0.025mM ZnCl_2_, protease inhibitors, 20μg/ml sheared salmon sperm DNA, 0.1% Triton-X100). To remove proteins binding non-specifically to the beads or ssDNA, the extract was incubated for 20min at RT on a rotating wheel with 4mg ssDNA-loaded magnetic beads. The cleared supernatant was split and incubated with 2mg beads each loaded either with SPE2 or mutated SPE2M DNA in the presence of 100pmol/ml ss 30b oligonucleotide for 45min at RT on a rotating wheel. Beads were washed once in 30ml WB1 (20mM Hepes pH 7.9, 160mM KCl, 1mM EDTA, 2mM DTT, 2mM MgCl_2_, 0.025mM ZnCl_2_, protease inhibitors, 20μg/ml sheared salmon sperm DNA, 100pmol/ml ss 30b oligonucleotide, 0.1% Triton-X100) and twice in WB2 (WB1 lacking competitor DNA). Bound proteins were eluted with 300μl EB (20mM Hepes pH 7.9, 2M KCl, 2mM EDTA, 20% glycerol, protease inhibitors).

*Protein analysis and identification*

Proteins eluted from beads were precipitated with 10% TCA, washed twice in ice-cold acetone and air-dried. The protein pellets were dissolved in 50μl 100 mM Tris-HCl, pH 8.0 and the sample was digested with 2μg of trypsin (Promega, WI, USA) for 18 hrs at 37^o^C. The digest was acidified with TFA to 1% final concentration and the sample was centrifuged for 5 min at 12,000 rpm. Digested proteins were analysed by capillary liquid chromatography tandem MS (LC/MS/MS) using a 300SB C-18 trap column (0.3x50mm) (Agilent Technologies, Basel, Switzerland) connected to a 0.1 mm x 10 cm capillary separation column packed with Magic C18 (5 μm particle diameter). The capillary column was connected to an Orbitrap FT hybrid instrument (Thermo Finnigan, San Jose, CA, USA). A linear gradient from 2 to 60% solvent B (0.1% acetic acid and 80% acetonitrile in water) in solvent A (0.1% acetic acid and 2% acetonitrile in water) in 85 min was delivered with a Rheos 2200 pump (Flux Instruments, Basel, Switzerland) at a flow of 50 μl/min. A pre-column split was used to reduce the flow to approximately 500 nl/min. 10 μl sample was injected with an autosampler thermostatted to 4°C onto the trap column for efficient desalting. The eluting peptides were ionized at 1.6 kV. The mass spectrometer was operated in a data-dependent fashion so that peptide ions were automatically selected for fragmentation by collision-induced dissociation (MS/MS) in the Orbitrap. The MS/MS spectra were then searched against a combined *P. falciparum*/human annotated protein database using TurboSequest software [51]. Post-search filtering of the search was set to ΔCn 0.1, the Xcorr vs. charge was 1.5 for singly, 2.0 for doubly, and 2.5 for triply charged ions, peptide probability was 0.5, and protein probability was 0.01. For a positive identification, a minimum of two unique peptides were accepted.
