# Supplementary material for: A Major Role for the Plasmodium falciparum ApiAP2 Protein PfSIP2 in Chromosome End Biology
Source: PLoS Pathog. 2010 Feb 26;6(2):e1000784. doi: 10.1371/journal.ppat.1000784 (PMC2829057; doi:10.1371/journal.ppat.1000784)
Supplement: Protocol S2 — Plasmid constructs. (0.03 MB DOC) [file ppat.1000784.s011.doc]

**Protocol S2**

**Plasmid constructs**

*Transfection constructs*

The first 1161bp (aa 1-387) of the PFF0200c ORF were amplified using primers 200*BamH*I F and 200*Nco*I R and cloned into pBcam-3xHA [27] to obtain pBcamSIP2-N-3xHA. pBcamSIP2-N-2xTy was generated by excision of the 3xHA tag in pBcamSIP2-N-3xHA with *Nco*I/*Sal*I and replacement with a 93bp ds oligonucleotide encoding a 2xTy tag. pSIP2-2xTy_3'RP was generated by replacement of the *Pst*I-*Nco*I fragment (rep20 repeat, *cam* promoter, *pfhp1*) in pHcamHP1-2xTy [27] with a 702bp PCR fragment encoding the 3' end of PFF0200c. pHBupsB^R^ carrying the PFL0005w upsB promoter (-2621 to -1) was described previously [54]. pHBupsB^R^ΔSPE2 was generated by replacing the *cam* promoter in pHBcam^R^ [24] with the PFL0005w upsB promoter lacking SPE2 repeats (-2071 to -1).

*Plasmids for recombinant protein expression*

Sequences were amplified from 3D7 gDNA and cloned into *BamH*I/*Xho*I-digested pET24a(+) (Novagen) for C-terminal 6xHIS-tagged proteins (SIP2-N-HIS_A, aa 1-390; SIP2-N-HIS_B, aa 171-390), or into *EcoR*I/*Xho*I-digested pET41a(+) (Novagen) for N-terminal GST-fusions (SIP2-AP2_1, aa 174-252; SIP2-AP2_2, aa 231-311; SIP2-AP2_12, aa 174-311).
