# Supplementary material for: A Major Role for the Plasmodium falciparum ApiAP2 Protein PfSIP2 in Chromosome End Biology
Source: PLoS Pathog. 2010 Feb 26;6(2):e1000784. doi: 10.1371/journal.ppat.1000784 (PMC2829057; doi:10.1371/journal.ppat.1000784)
Supplement: Protocol S3 — Quantitative reverse transcriptase PCR. (0.03 MB DOC) [file ppat.1000784.s012.doc]

**Protocol S3**

**Quantitative reverse transcriptase PCR**

Parasites were synchronised twice 34hrs apart to obtain a 10hr growth window. Total RNA was isolated at six timepoints across the IDC (6-16hpi; 12-22hpi; 18-28hpi; 24-34hpi; 32-42hpi; 38-48hpi) using TriReagent (Sigma-Aldrich) and further purified using the RNeasy Plus Mini Kit (Qiagen) for removal of gDNA. Residual gDNA was digested with TURBO DNA-*free*^TM^ (Ambion). All samples were tested negative for contaminating gDNA by qPCR. RNA was reverse transcribed using the RETROscript Kit® (Ambion). qPCR reactions for PF13_0170, *msp8*, *cam* and h*dhfr* absolute transcript quantification were performed at final primer concentrations of 0.4μM using SYBR® Green Master Mix (Applied Biosystems) on a StepOnePlus^TM^ Real-Time PCR System (Applied Biosystems) in a reaction volume of 12ul. Plasmid copy numbers were determined by qPCR on gDNA isolated from the same parasite samples and calculated by dividing the absolute copy numbers of h*dhfr* by the average value obtained for the *msp8*, PF13_0170 and *cam* loci. All reactions were run in triplicate yielding virtually identical Ct values. Serial dilutions of gDNA and plasmid DNA were used as standard for absolute quantification. Relative transcript profiles were calculated by normalisation against the house-keeping gene PF13_0170 (glutaminyl-tRNA synthetase). For h*dhfr*, this value was additionally adjusted for plasmid copy numbers.
