# Supplementary material for: A Major Role for the Plasmodium falciparum ApiAP2 Protein PfSIP2 in Chromosome End Biology
Source: PLoS Pathog. 2010 Feb 26;6(2):e1000784. doi: 10.1371/journal.ppat.1000784 (PMC2829057; doi:10.1371/journal.ppat.1000784)
Supplement: Protocol S4 — Oligonucleotide sequences used in this study. (0.04 MB DOC) [file ppat.1000784.s013.doc]

**Protocol S4**

**Oligonucleotide sequences used in this study**

**Cloning (restriction sites are in bold):**

SIP2-N-HA F gatc**ggatcc**aaacaatggaagataatttagttaaag

SIP2-N-HA R gatc**ccatgg**aactttatgattcaaagtgctac

2xTy F **catgg**gtgcttagg***gaagtacatactaaccaagatcctttggatgcaggtgct***

***gaggttcataccaatcaagacccacttgat***gagctctaa**g**

2xTy R **tcgac**tta**gagctc*atcaagtgggtcttgattggtatgaacctcagcacctgcat***

***ccaaaggatcttggttagtatgtacttc***cctaagcac**c**

SIP2Ty F ctag**ctgcag**aaatatggtgttaacatatacatg

SIP2Ty R ctag**ccatgg**tttgtctacatgttcatttatct

4A3ΔSPE2 F cgc**ggatcc**tttgtttatcgttcgtgactacattatgtc

4A3ΔSPE2 R cagt**ggatcc**aattttaggatctaggtgttgtc

SIP2-N-HIS_A F tata**ggatcc**atggaagataatttagttaaagaatc

SIP2-N-HIS_A/SIP2HIS_B R agag**ggatcc**aaaatgagagaacacataggtagcc

SIP2-N-HIS_B F cttct**ctcgag**acttcttgtaactttatgattc

SIP2-AP2_1/SIP2_AP2_12 F aaagg**gaattc**agagaacacataggtagcc

SIP2-AP2_1 R aata**ctcgag**ctaattttcaaatactcgtactatag

SIP2-AP2_2 F gaat**gaattc**tctggaataacctttgaac

SIP2-AP2_2/SIP2_AP2_12 R agtt**ctcgag**ctaattaaatataccttttatcatac

**Protein pull-down (binding motif in bold capital letters, mutations in italics)**

SPE2 F biotin-gactttat**TGTGCATAGTGGTGCG**atatag

SPE2 R ctatat**CGCACCACTATGCACA**ataaagtc

SPE2M F biotin-gactttat**T*T*T*T*CATAGTG*T*T*T*CG**atatag

SPE2M R ctatat**CG*A*A*A*CACTATG*A*A*A*A**ataaagtc

**qPCR**

PF13_0170 F tggctaggatatgattggaaagaaca

PF13_0170 R tacggttctatttctatatggtgaatca

msp8 F agtgctgtaacttctaatgtaggggatacaaat

msp8 R atcatcatcaccattatcatcattatcatcacg

hdhfr F cattcctgagaagaatcgaccttt

hdhfr R tctggttgttcagtaagttttaaggca

cam F atctttaggacaaaatccaactgaagcag

cam R cattaaggttagaaattcgggaaaatcgatc

**ChIP-qPCR**

**Primer Name Sequence 5'-3' Locus Pos. rel. ATG SPE2 sites**

PFL0005w -2.3 F catagtggtgcgaatttatact upsB var -2364-2220bp multiple

PFL0005w -2.3 R gacaacacctagatccta

PFL0005w -2.2 F ccacatatatagtgatacc upsB var -2226-2047bp multiple

PFL0005w -2.2 R cactatgcacaataaagtc

PFL0005w -1 F agtagaattaagagaagaag upsB var -1284-1143bp

PFL0005w -1 R attctcggttactgcatg

PFL0005w 2 F aaagaaaaggaatggaagg upsB var +1941-2101bp

PFL0005w 2 R attttcgctatgtgttgtg

PFL0005w 3 F agtggtaaccataatgttg upsB var +6775-6871

PFL0005w 3 R atgtatttgtatgttttgtatg

TARE catgtaaatttcgggcccca TARE multiple

TARE ttgagaaggtgcttaaacccctc

PFL0935c -1 F cctaaatactatattttgagac central upsB -2250bp two

PFL0935c -1 R cacatggaacacaactag

PFB0935w -1 F cttttcctttttgttgcac clag2 -500bp

PFB0935w -1 R tttcgacaaacgaatatgc

PFI1475w -1 F aaacatagtattagatttactc msp1 -1000bp

PFI1475w -1 R caattaacatttacaagtgatc

PFB0345c -1 F atgaaatttatattgtggtg sera4 -300bp

PFB0345c -1 R ctaatgaaatattatgagag

PFL1025c -1 F tccttcttgaaatatggtc hyp -700bp

PFL1025c -1 R catgaaaattataagatgcttttg

MAL7P1.119 -1 F cttataagaacgaaataagtg hyp -500bp

MAL7P1.119 -1 R ctcaatataaaataagcactg

PFI0265c -1 F cacattttgatatgtgtatac rhoph3 -1500bp single

PFI0265c -1 R gacacgctattaatttttcg

PFE0075c -1 F ccatttaaacaaaatcagtg rap3 -1000bp single

PFE0075c -1 R ttttatgcatgtgaagtgc

PFL2505c -1 F atggtatgtgtactataatc rnp3 -800bp single

PFL2505c -1 R ctaatgggtgtactgaaac

PFF0645c -1 F actatttccattcatttagg imp -800bp single

PFF0645c -1 R tgaaacaagagtaaattacc

PFL1090w -1 F ttgcaatgaaatatttgtgc gap45 -1050bp single

PFL1090w -1 R gaaattctcataataacaatagg

**Affinity purification of the SPE2-binding activity (SPE2 and SPEM motifs in bold)**

SPE2concat:

F P-gactttat**tgtgca**tagt**ggtgcg**aatttatacttt**ggtgca**acta**ggtgca**acattttacttt**tgtgca**acta**ggtgca**a

R a**acacgt**atca**ccacgc**ttaaatatgaaa**ccacgt**tgat**ccacgt**tgtaaaatgaaa**acacgt**tgat**ccacgt**tctgaaat-P

SPE2biotin:

F biotin-gtttatacttt**tgtgca**tagt**ggtgcg**aattt

R caaatatgaaa**acacgt**atca**ccacgc**ttaaactgaaat-P

SPE2Mconcat:

F P-gactttat**tcttca**tagt**gtttcg**aatttatacttt**gcttca**acta**gtttca**acattttacttt**tcttca**acta**gtttca**a

R a**agaagt**atca**caaagc**ttaaatatgaaa**cgaagt**tgat**caaagt**tgtaaaatgaaa**agaagt**tgat**caaagt**tctgaaat-P

SPE2Mbiotin:

F biotin-gtttatacttt**tcttca**tagt**gtttcg**aattt

R caaatatgaaa**agaagt**atca**caaagc**ttaaactgaaat-P

SSCOMPbiotin:

F biotin-agaaatgtggtagataatatagatagaaatgtggtagataatatagatagaaatgtggtagataatatagatagaaag
